# Ketoprofen-Based Ionic Liquids: Synthesis and Interactions with Bovine Serum Albumin

**DOI:** 10.3390/molecules25010090

**Published:** 2019-12-25

**Authors:** Paula Ossowicz, Proletina Kardaleva, Maya Guncheva, Joanna Klebeko, Ewelina Świątek, Ewa Janus, Denitsa Yancheva, Ivan Angelov

**Affiliations:** 1 Department of Chemical Organic Technology and Polymeric Materials, Faculty of Chemical Technology and Engineering, West Pomeranian University of Technology, Piastów Ave. 42, 71-065 Szczecin, Poland; paula.ossowicz@zut.edu.pl (P.O.); joanna.klebeko@gmail.com (J.K.); ewelinaswiatek94@gmail.com (E.Ś.); ejanus@zut.edu.pl (E.J.); 2 Institute of Organic Chemistry with Centre of Phytochemistry, Bulgarian Academy of Sciences, Acad. G. Bonchev Str. Bl. 9, 1113 Sofia, Bulgaria; pkardaleva@orgchm.bas.bg (P.K.); deni@orgchm.bas.bg (D.Y.); ipangelov@orgchm.bas.bg (I.A.)

**Keywords:** ketoprofen, ionic liquids, bovine serum albumin, binding constants, secondary structure

## Abstract

The development of ionic liquids based on active pharmaceutical ingredients (API-ILs) is a possible solution to some of the problems of solid and/or hydrophobic drugs such as low solubility and bioavailability, polymorphism and an alternative route of administration could be suggested as compared to the classical drug. Here, we report for the first time the synthesis and detailed characterization of a series of ILs containing a cation amino acid esters and anion ketoprofen (KETO-ILs). The affinity and the binding mode of the KETO-ILs to bovine serum albumin (BSA) were assessed using fluorescence spectroscopy. All compounds bind in a distance not longer than 6.14 nm to the BSA fluorophores. The estimated binding constants (KA) are in order of 10^5^ L mol^−1^, which is indicative of strong drug or IL-BSA interactions. With respect to the ketoprofen-BSA system, a stronger affinity of the ILs containing l-LeuOEt, l-ValOBu, and l-ValOEt cation towards BSA is clearly seen. Fourier transformed infrared spectroscopy experiments have shown that all studied compounds induced a rearrangement of the protein molecule upon binding, which is consistent with the suggested static mechanism of BSA fluorescence quenching and formation of complexes between BSA and the drugs. All tested compounds were safe for macrophages.

## 1. Introduction

Ionic liquids (ILs) are organic salts consisting of bulky organic cations and organic or inorganic anions [1]. In general, they are characterized by low melting temperature (<100 °C), low vapor pressure, low flammability, high thermal stability, etc. [1]. Their physicochemical properties can be easily tuned by appropriate selection of the cation and the anion or an introduction of substituents in the molecule of cation or anion. Therefore, in the last few decades, they have attracted great interest in view of their potential diverse applications. The application of ILs in pharmaceutics and medicine is a relatively new research area. Numerous studies have shown the potential of ILs as drug-delivery systems, biomedical analytics, sensors, excipients, solvents and stabilizers of biomolecules, [2,3,4,5,6]. On the other hand, some ILs exhibit biological activities, e.g., antibacterial, antitumor, and other [7,8,9,10]. Further, the development of ILs based on active pharmaceutical ingredients (API-ILs), single active liquid salts, is a possible solution of some of the problems of solid and/or hydrophobic drugs such as low solubility and bioavailability, polymorphism and an alternative route of administration could be suggested as compared to the classical drug [11,12]. In addition, ILs based on two biologically active ions have been also investigated, e.g., ILs containing a cation with anti-bacterial or pain-reliever properties and an anion anti-inflammatory, antibacterial, anti-pyretic activity, or wetting agents and emollients [13,14]. They have the advantage to retain the biological activities inherited from the two API-precursors [11,15].

ILs based on a non-steroidal anti-inflammatory drug such as ibuprofen (Ibu), naproxen, and ketoprofen (KETO) have been in the focus of recent investigations. Santos et al. proposed a novel formulation based on ammonium, imidazolium, or pyridinium (Pyr) cations and ibuprofen anion, which characterized with enhanced water solubility in comparison to that of the sodium ibuprofenate (control) [16]. Except for [C16Pyr][Ibu], all the other Ibu-based ILs exhibited less cytotoxic effect to normal human dermal fibroblasts or ovarian carcinoma (A2780) cell line in comparison to the parent sodium ibuprofenate [16]. An enhanced topical anti-inflammatory activity was reported for a series of ILs containing cations terpenes and anion Ibu [17]. Interestingly, Ibu- based ILs containing cations ranitidinium or diphenhydraminium exhibited activity against *Candida* sp., which was not inherited from the parent salt—however, they are shown to be more cytotoxic to human lymphocytes [14]. Based on IL_50_ values estimated for the inhibition of cytochrome C oxidase, Azevedo et al. have found that the cholinium ketoprofenate (IC_50_ ≈ 21.3 mM) produced a lower cytotoxic effect than sodium ketoprofenate (IC_50_ ≈ 0.0006 mM) and therefore it is considered safer [18]. On the contrary, cholinium naproxenate is more toxic than its parent sodium salt [18].

Serum albumins are the most abundant proteins in mammalian plasma responsible for the transport of both exogenous and endogenous molecules such as hormones, steroids, fatty acids, drugs, etc., as well as play an essential role in the maintenance of the blood oncotic pressure and pH [19]. The bovine serum albumin (BSA) is highly homologous (76%) to human serum albumin and is very often used as a model protein to study the mechanism of binding and interactions of small molecules with the protein [20]. BSA is a single-chain globular heart-shaped protein composed of three homologous domains (I, II, and III) each domain includes two subdomains (A and B). The subdomains IIA and IIIA are responsible for drug binding [19]. The geometry of the two binding pockets is quite different, the larger one is IIA subsite and it specifically binds molecules such as warfarin, salicylates, penicillin, while the IIIA subdomain is narrower one and it binds ibuprofen, ketoprofen, benzodiazepines, and some others [19]. To study the drug-serum albumins interactions is of importance to understand pharmacokinetics and pharmacodynamics of the drugs, i.e., to reveal the factors affecting their absorption, distribution, metabolism, and excretion of the drugs. 

To the best of our knowledge, besides the paper of Azevedo et al., no other reports on the KETO-based ILs have been published [18]. Here, we report the synthesis, identification, and characterization of a series of ILs containing as a cation the amino acid ester and an anion KETO. Considering the information from the literature on the low toxicity of ethyl esters of non-polar amino acids, we made the selection of the structure of L-valine and L-leucine esters as the cation [21]. Apart from the ethyl esters of these amino acids, for L-valine we have also obtained esters with longer chain alcohols. Our primary goal was to investigate the interaction of the KETO-ILs with BSA. We assessed the affinity and the binding mode of the KETO-ILs to BSA using fluorescence spectroscopy. The effect of the compounds on the conformation of BSA was monitored using Fourier transformed infrared spectroscopy (FTIR). The cytotoxicity of the compounds against the murine macrophage cell line (RAW 264.7) was evaluated. 

## 2. Results

### 2.1. Synthesis of the Amino-Acid Ester-Ketoprofenate

**Scheme 1:**
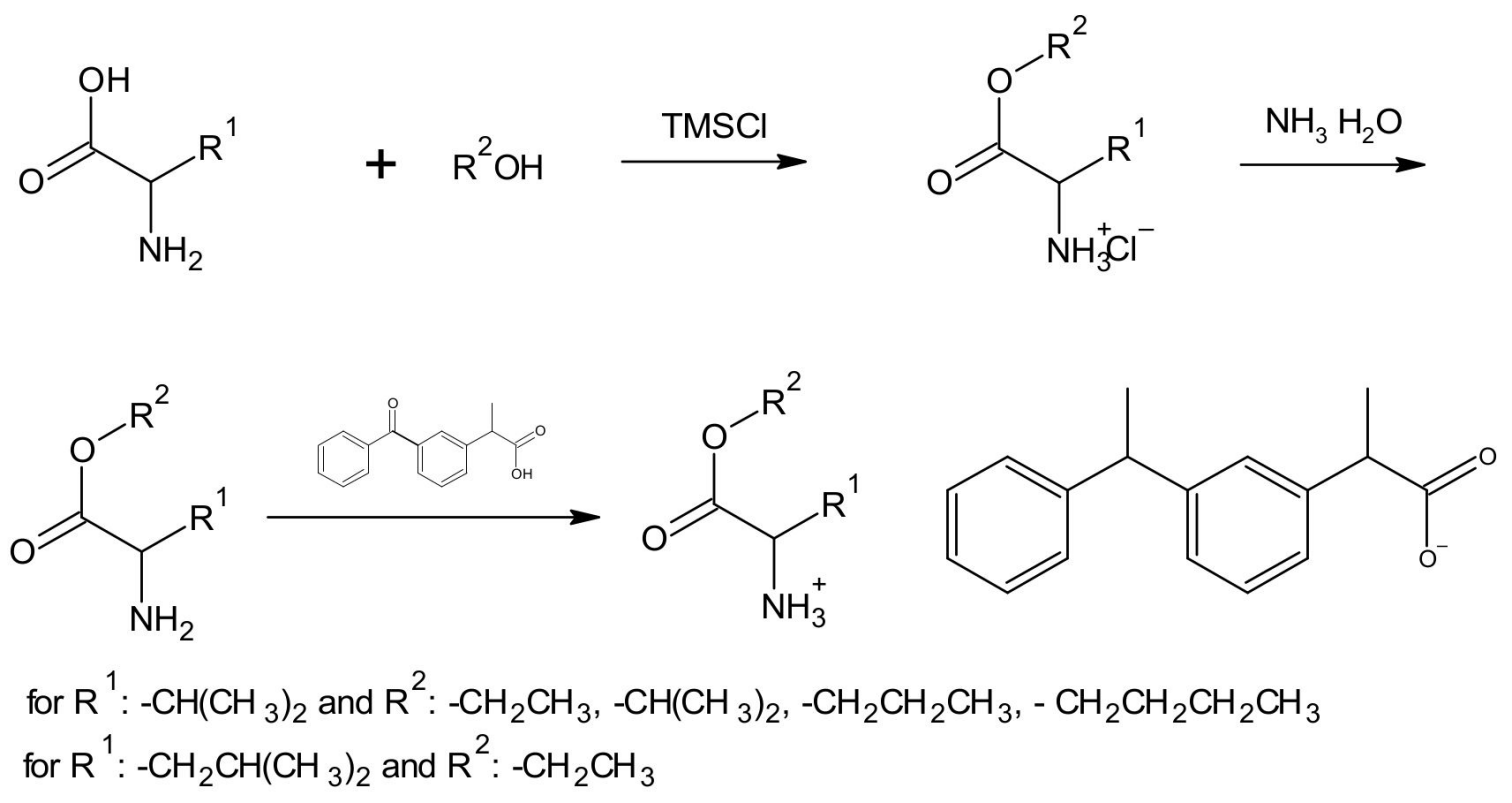
General synthetic procedure for [AAOR][KETO].

Using a three-step method, we synthesized five novel ketoprofen-based ILs containing cation amino acid esters (Scheme 1). 

To assess the impact of the chain-length of the alcohol on BSA-IL interactions, four of the selected for the synthesis compounds were esters of l-valine (Val) with various alcohols (ethanol, propanol, isopropanol, and butanol). The ethyl ester of l-leucine was also obtained in order to evaluate the effect of the size of the amino acid side chain on the biological activity and protein IL interactions. Besides, short-chain esters of non-polar amino acids were expected to be not cytotoxic to cells. This assumption is based on the reported in the literature low toxicity of salicylic acid-based ILs having cation l-alanine ethyl ester or l-leucine ethyl ester on human cervical cancer cells (HeLa) and murine fibroblasts (L929), for which the determined IC_50_ values were in the millimolar range [21]. The compounds were obtained in high yields (90–95%) and were identified in detail by ^1^H-NMR, ^13^C-NMR, FTIR and elemental analysis (See Section 3 and Figures S1–S32, Supplementary Materials). These analyses confirmed that synthesized compounds are organic salts with amino acid cation and ketoprofenate anion. The NMR spectra clearly show signals for the protonated NH_2_ group of the amino acid, with a chemical shift in the range between 4.84 for [L-ValOPr][KETO] to 6.67 for [l-LeuOEt][KETO], for which the integration corresponds to three protons. Another piece of evidence is the presence of strong bands at ca. 1570 and 1390 cm^−1^ in FTIR spectra, assigned to the symmetric and asymmetric stretching vibrations of carboxylate anion v(COO^−^)_sym._ and v(COO^−^)_as._, respectively [22,23]. In addition, the performed spectroscopic analyses completely exclude the formation of an imine between the ketoprofen carbonyl group and the amino group of amino acid, which literature has reported for ketoprofen and isoniazid [24]. On the ^13^C-NMR spectra of all obtained KETO-ILs, there is not observed the characteristic signal for imine with a chemical shift between 162 and 164 ppm. Moreover, in FTIR spectra there are not the characteristic bands for Schiff bases, neither the vibrational band of N–H at 3355 cm^−1^ nor C=N stretching vibration band at 1657 cm^−1^ [25]. 

KETO-ILs are solids with melting points below 100 °C, which within the series, decreases with increasing the alkyl chain-length in the ester group of the amino acid cation (Table 1). Among the synthesized ketoprofen salts, the derivative obtained by pairing with L-leucine ethyl ester had the highest melting point (m.p. 94.23 °C), which was higher than for the parent acid form. Melting points of ketoprofen salts with L-valine esters characterized melting points comparable to that for choline ketoprofenate (m.p. 62–65 °C) demonstrated by Ribeiro et al. [26]. Tetrabutylammonium salt of ketoprofen is also known from the literature [27], but authors had not given the melting point of that. According to the confirmed definition, the synthesized KETO-ILs can be qualified as the protic ionic liquids [28]. 

The thermal stabilities of the obtained KETO-ILs were investigated by TG. The onset temperature of the mass loss was determined (T_onset_, Table 1). The thermal degradation of received compounds takes place at the onset of the first stage. The onset temperature was in the range of 52.3–100.4 °C, whereas the value for ketoprofen was 265.4 °C. For this reason, the synthesized KETO-ILs showed lower thermal stability than parent ketoprofen. No direct relationship of thermal stability was found with the increasing length of the alkyl chain in the ester group of the amino acid cation. The highest onset temperature, close to 100 °C, was demonstrated for [l-LeuOEt][KETO] and [l-ValOBu][KETO], while the lowest was found for [l-ValOPr][KETO]. The structure of the amino acid side chain has an only small effect on stability KETO-ILs—slightly higher stability was established for the L-valine cation than for L-leucine. All obtained ketoprofen derivatives are chiral (two chiral center) with specific rotation listed in Table 1.

The solubility in water and conventional organic solvents have been investigated and summarized in Table 2. The solvents were ranked with a decreasing value of empirical polarity parameters (E_T_(30)) [29]. According to the used Vogel’s methodology for estimation of solubility, these compounds were classified as insoluble in water, because their solubility is below 33.3333 g L^−1^. All KETO-ILs were also insoluble in non-polar solvents such as *n*-hexane. Generally obtained KETO-ILs were soluble or partly soluble in ethanol and chloroform. Most of them were insoluble or partly soluble in diethyl ether and well soluble in toluene with the exception of [l-ValOEt][KETO]. 

### 2.2. Binding Constants and Binding Sites for the Interactions of Bovine Serum Albumin (BSA) with the Ketoprofen-Based Ionic Liquids (ILs)

According to the literature, ketoprofen binds in the site 1 of domain IIA of BSA [31]. This binding pocket is highly hydrophobic, and the binding is assisted by stacking interaction of the aromatic rings of ketoprofen molecules with the hydrophobic amino acid residues comprising this binding site, namely one tryptophane (Trp213), four leucines, two isoleucines, two alanines, and one phenylalanine [31]. Additionally, the carboxylic group of the ketoprofen is hydrogen-bonded with the hydroxyl group of Tyr149, the imidazole nitrogen atoms of His241, and the guanidine group of Arg256 which ensure strong interactions [31].

Fluorescence spectroscopy is a useful tool for the investigation of the interactions of proteins with small molecules. The method gives information on the binding mode, binding sites, and binding constants of small molecules to protein and can be applied for monitoring of the induced conformational changes. The fluorescence of BSA is due to tryptophan, tyrosine, and phenylalanine residues that present in the protein molecule. At excitation wavelength fixed at 280 nm, the fluorescence spectrum of BSA is characterized by a strong emission band with a maximum at 355 nm.

Upon the addition of small molecules to the solution, a decrease in the BSA fluorescence may occur. The process is typically ascribed either to the formation of a non-fluorescence-quencher complex in the ground state, a process referred to as static quenching, or the quenching could be due to collisions between the excited fluorophore and quencher (dynamic quenching) [32]. 

**Figure 1:**
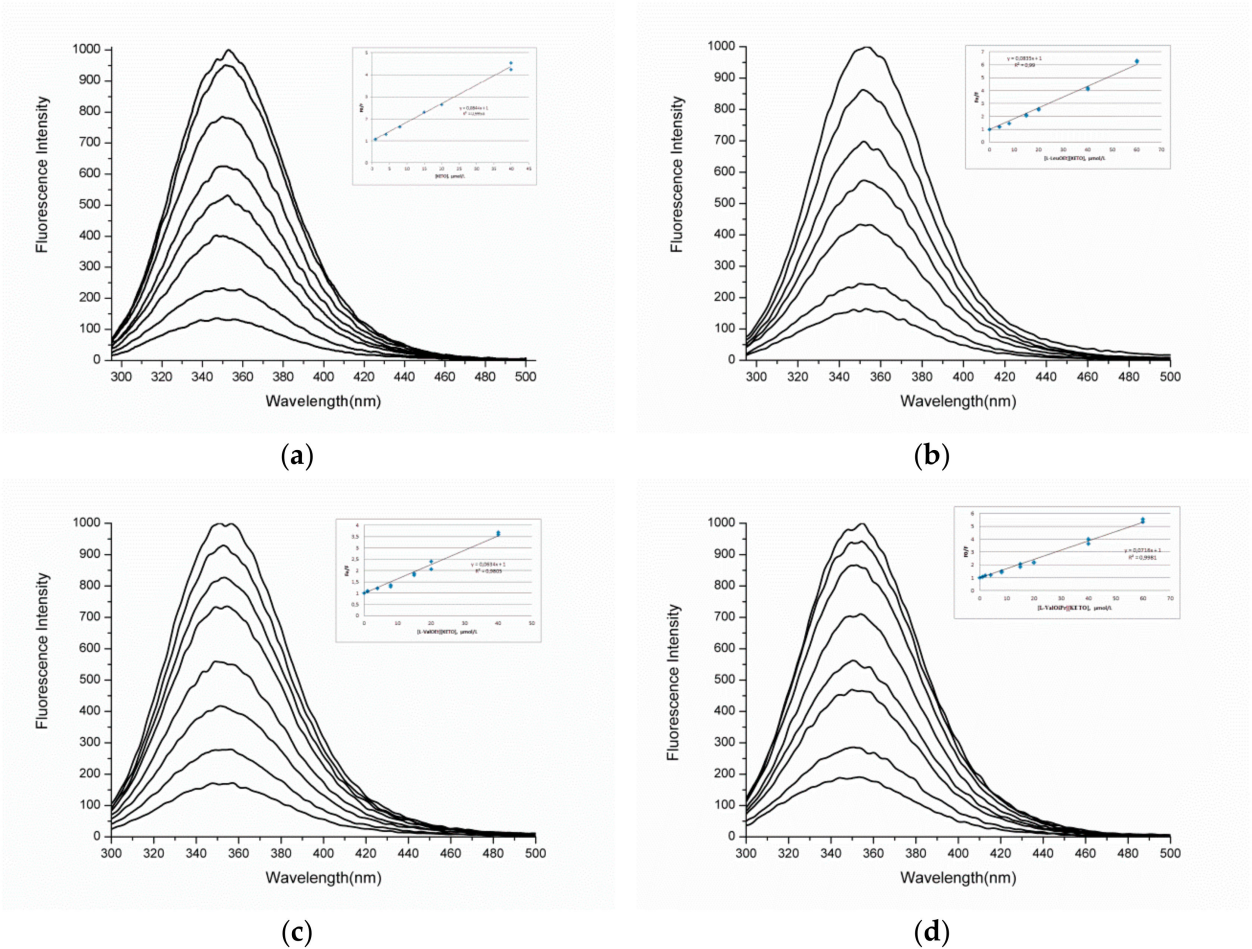

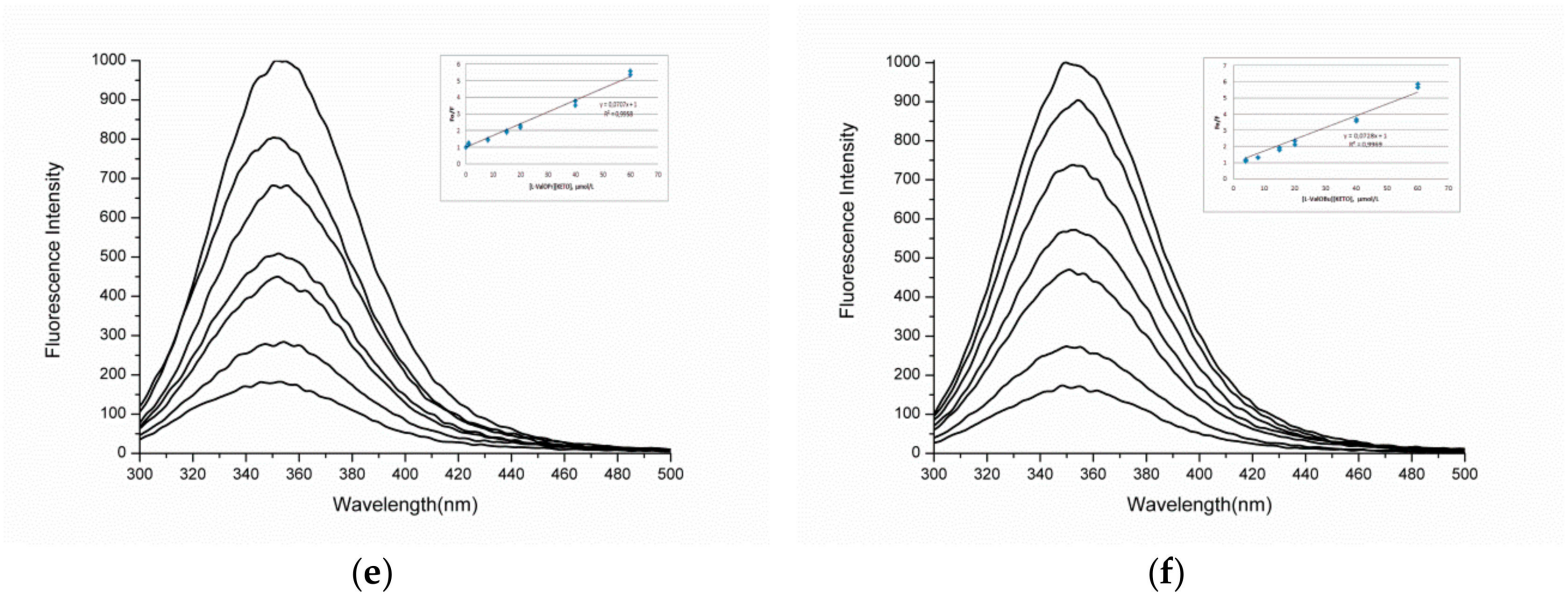
Change in the fluorescence emission spectra of BSA in the presence of various concentration of ketoprofen (**a**); [l-LeuOEt][KETO] (**b**); [l-ValOEt][KETO] (**c**); [l-ValOiPr][KETO] (**d**) [l-ValOPr][KETO] (**e**) and [l-ValOBu][KETO] (**f**) in sodium phosphate buffer (pH 7.4, 50 mM) at 25 °C. The insertions represent the Stern–Volmer plot.

The emission spectra of BSA in the presence of various concentrations of ketoprofen and KETO-ILs were recorded in the range from 300 to 500 nm (λ_ex_ = 280 nm). We have observed a significant decrease in the fluorescence intensities of the BSA fluorophores with increasing the concentration of the tested compounds added to the BSA solution (Figure 1), which suggests binding of the tested compounds in the protein molecule. The change in the local environment near the fluorophores was insignificant, which is evident from the slight blue shift of the emission maxima by up to 1 or 2 nm. The fluorescence quenching data are analyzed according to the Stern–Volmer Equation (1) [32]:

(1) $$\frac{Fo}{F}=1+k_{q}\tau_{q_{o}}[Q]=1+K_{sv}[Q],$$

where *F_o_* and *F* is the fluorescence peak intensities in the absence and in the presence of quencher. [*Q*] is quencher (KETO-IL or ketoprofen) concentration, KSV is the Stern–Volmer quenching constant, *k_q_* is the bimolecular quenching constant (quenching rate constant), and *τ_qo_* is the average life-time of the protein without quencher, generally taken to be 10^−8^ s [33].

The obtained *K_sv_* and *K_q_* values were summarized in Table 3. The maximum scatter quenching constant *K_q_* that is due to collisions between biomolecules is 2 × 10^10^ L mol^−1^ s^−1^ [33]. The rate constant of BSA quenching due to KETO-ILs is greater than *K_q_* of scatter procedure, therefore, we assume that a static quenching mechanism is more likely to occur. The conclusion agrees with the report of Bi et al., who demonstrated that the quenching mechanism of HSA by ketoprofen is static [33]. The *K_sv_* of binding of ketoprofen with BSA was in the same range as that reported for the ketoprofen with HSA [33].

The estimated *K_sv_* and *K_q_* values are of the same order of magnitude and the structure of the cation seems not to affect the binding properties of the active pharmaceutical ingredient (ketoprofen).

If the [AAOR][KETO] and ketoprofen bind independently to a set of equivalent sites on the BSA molecule, the effective binding constant (*K_A_*) and the number of binding sites (*n*) can be calculated using the following Equation (2):

(2) $$\log(\frac{Fo-F}{F})=\log K_{A}+n\log[Q],$$

where *F_o_*, *F*, and [*Q*] have the same meaning as Equation (1). The dependence of $\log(\frac{Fo-F}{F})$ vs. log[*Q*] is linear with a slope equal to the number of the binding sites, and y-intercept corresponds to the log of the effective binding constant (*K_A_*). The estimated values of *K_A_* and *n* are listed in Table 4 (Figure S33, Supplementary Materials).

The obtained binding constants (*K_A_*) are in order of 10^5^ L mol^−1^, which is indicative of strong drug or IL-BSA interactions. With respect to ketoprofen-BSA system, a stronger affinity of the ILs containing l-LeuOEt, l-ValOBu and l-ValOEt cation towards BSA is clearly seen. 

According to the Förster theory, the nonradiative energy transfer rate (FRET) between a donor (BSA fluorophores) and an acceptor (Ketoprofen or KETO-ILs) depends on the extent of the overlapping of the donor emission spectrum and the acceptor absorption spectrum, the relative orientation of the donor and acceptor transition dipoles, as well as from the distance between the donor and acceptor. The following equations are used to estimate the steady-state FRET parameters:

(3) $$E=1-\frac{F}{Fo}=\frac{Ro^{6}}{Ro^{6}+{r_{o}}^{6}},$$

(4) $$Ro^{6}=8.8x10^{-25}K^{2}n^{-4}\phi J,$$

(5) $$J=\frac{\sum F(\lambda )\varepsilon (\lambda )\lambda^{4}\Delta \lambda }{\sum F(\lambda )\Delta \lambda },$$

where *E* is energy transfer efficacy, *R_o_* is the characteristic distance called Förster distance or critical distance at which the energy transfer efficacy is 50%, *r_o_* is the distance between the quencher (drug) and the BSA fluorophores. *K*^2^ is the spatial orientation factor describing the relative orientation in the space of transition dipoles of the donor and the acceptor, *K*^2^ = 2/3, n is the refractive index of the medium, *φ* is the fluorescence quantum yield of the donor, n = 1.33, *φ* = 0.13 [34]. *F*(*λ*) is the fluorescence intensity of the fluorescence of the donor, *ε*(*λ*) (L mol^−1^ cm^−1^) is the molar absorption coefficient of the acceptor at wavelength *λ*. The estimated FRET values are listed in Table 5.

As seen, for the ketoprofen and all KETO-ILs the donor-acceptor distance is *r_o_* is less 8 nm [34], which is in accordance with Förster’s non-radiative energy transfer theory and is a proof of that the BSA fluorescence quenching is a result of the formation of a nonfluorescent ground state fluorophore-compound complex, i.e., static quenching mechanism.

### 2.3. Changes in the Secondary Structure of BSA upon Binding of the Ketoprofen-Based ILs

We applied FTIR spectroscopy to study the effect of KETO-ILs on the secondary structure of BSA. In the spectra of proteins, the Amide I band region (1700–1600 cm^−1^) is mainly due to the starching vibrations of the carbonyl group (80%) and is highly sensitive to the changes in the geometry and the environment of the amide bonds [34]. The original spectra, the second derivative and the deconvoluted spectra of the complexes of BSA with KETO-ILs are given in Supplementary (Figures S34–S39). The band assignments were done according to Jackson and Mantsch and the analysis of the structure was taken into consideration the data on the BSA available in the literature [35,36,37].

For each BSA-drug complex, are observed several bands having a center within 1610–1628, 1625–1640, 1640–1648, 1648–1660 and 1675–1698 cm^−1^, which are characteristic for aggregated strands, β-sheets (low frequency), distorted/random coils, α-helices, β-sheets (high frequency) or extended conformation [35]. The Amide I band components, their relative area and the assignment for the native BSA and its complexes with the studied compounds are given in Table 6.

As seen all studied compounds induced a rearrangement of the protein molecule upon binding, which is consistent with the suggested static mechanism of BSA fluorescence quenching and formation of complexes between BSA and the drugs. In the presence of ketoprofen the helical structure of the protein was preserved, however, we observed a complete loss of the band at 1943 cm^−1^, which is ascribed to the random coils and unordered structures, in favor of the β-structures. KETO-ILs stimulated the partial unfolding of BSA and we monitored a decrease in the α-helical content and an increase in antiparallel β-sheets and aggregates in this BSA-IL complex. No shift of the peak positions and very close to the native BSA conformation was observed for the BSA-[l-LeuOEt][KETO] system. In addition, [l-ValOiPr][KETO] caused the most dramatic changes in the secondary structure, namely a significant loss of helical structures and aggregation, which is consistent with the observed slight lowering of the estimated binding constant. It is noteworthy to be mentioned that the conformational changes seem not to affect the binding properties of the BSA. On the contrary, the ILs with [l-LeuOEt], [l-ValOEt] and [l-ValOBu] cations even exhibited slightly higher affinity to BSA.

### 2.4. Cytotoxicity of the Ketoprofen-Based ILs on Murine Macrophages

**Figure 2:**
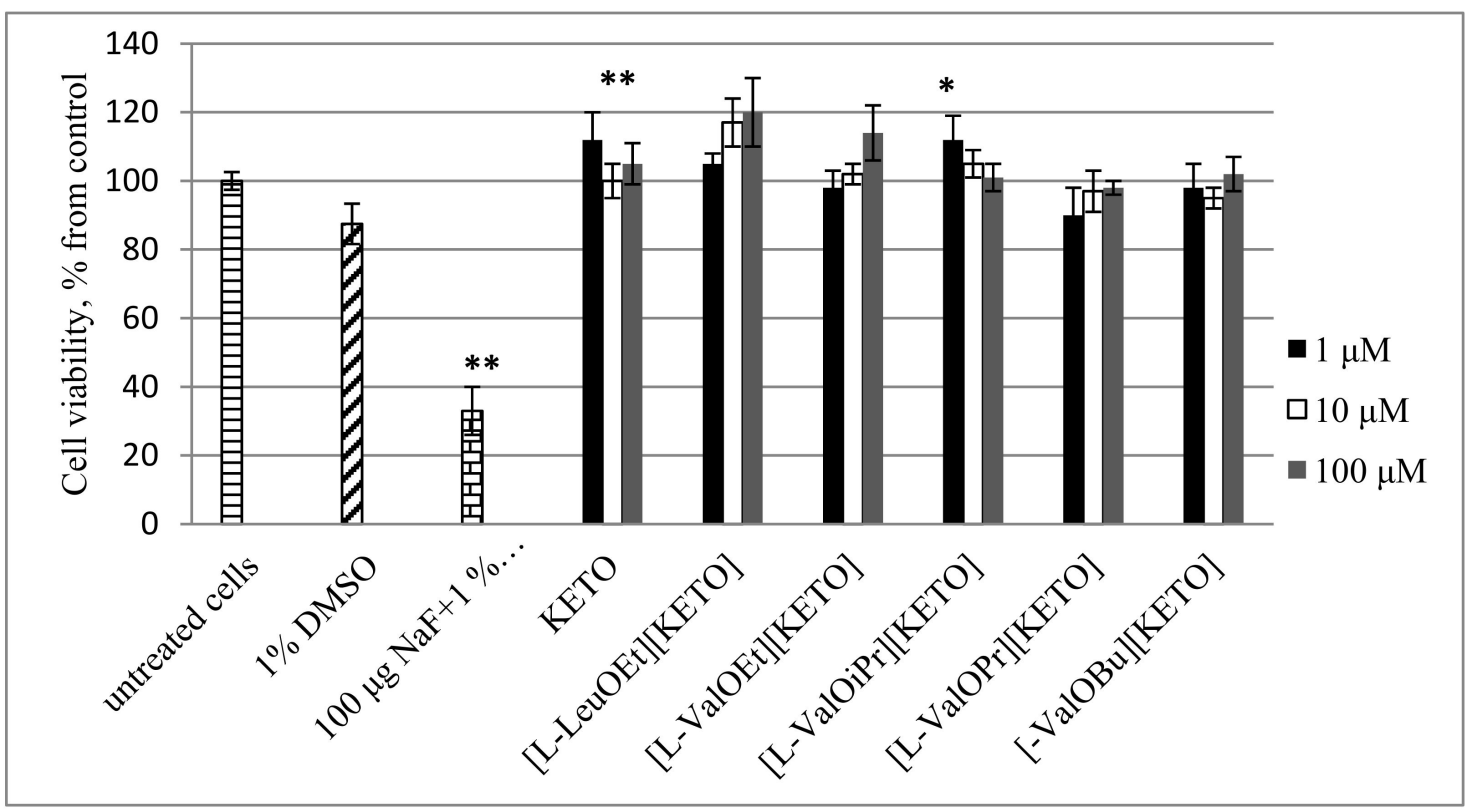
Effect of ketoprofen-based ionic liquids (ILs) on the percentage of cell viability in RAW 264.7 macrophages. Control cells were treated with media alone. Values are the mean ± standard error of the mean of three independent experiments. ** *p* < 0.01, * *p* < 0.05 indicates.

The effect of ketoprofen-based ILs was evaluated in RAW 264.7 macrophages after 24 h incubation with different concentrations of the compounds (1, 10 and 100 μM). As controls were used untreated cells, cells treated with 1% DMSO (the solvent in which the compounds are prepared) and 100 μg NaF and 1% DMSO. As seen in Figure 2 at the tested concentrations, the ILs were safe for macrophages. Moreover, the compounds even were able to stimulate cell proliferation and to minimize the toxic effect of the solvent.

## 3. Materials and Methods 

### 3.1. Materials

Ketoprofen was isolated from commercially available medicines in pills (Ketonal forte^®^ (100 mg) tablet manufactured by SANDOZ, Holzkirchen, Germany) by extraction with ethanol, then purification of the extract by filtration and then precipitation from extract with water. Identification of isolated compounds was performed by NMR analysis and purity was determined by acid-base (acidimetric) titration, according to the Pharmacopoeia and elementary analysis. l-valine and l-leucine with high purity (>99.0%) were purchased from Carl Roth (Karlsruhe, Germany). Trimethylsilyl chloride with high purity (≥99.0%) (TMSCl) was obtained from Sigma-Aldrich (Steinheim am Albuch, Germany). Ethanol (EtOH), propan-2-ol (*i*-PrOH), propan-1-ol (PrOH), butan-1-ol (BuOH), dimethyl sulfoxide, chloroform, ethyl acetate, diethyl ether, toluene and n-hexane and ammonia solution were high purity provided by Chempur (Gliwice, Poland). All reagents, solvents, and other materials were of analytical grade and were used without any further purification. Deuterated chloroform (CDCl_3_) (99.8%) (+0.03% TMSCl) was obtained from Eurisotop (Cheshire, England).

Bovine serum albumin (BSA), heat shock fraction, protease-free, fatty acid-free, essentially globulin free (pH 7, ≥98%), Dulbecco’s Modified Eagle’s Medium (DMEM) containing 4 mM L-glutamine, 4500 mg L^−1^ glucose, 1 mM sodium pyruvate, and 1500 mg L^−1^ sodium bicarbonate, trypsin-EDTA solution, and penicillin–streptomycin–neomycin stabilized solution were purchased from Merck (Darmstadt, Germany). 

Murine macrophages RAW 264.7 was obtained from the American Tissue Culture Collection (ATCC) (Manassas, VA, USA). Alamar Blue cell viability reagent was purchased from Thermo Scientific (Waltham, MA, USA).

### 3.2. Synthesis of the Ketoprofen Amino Acid Alkyl-Esters [AAOR][KETO]

**Scheme 2:**
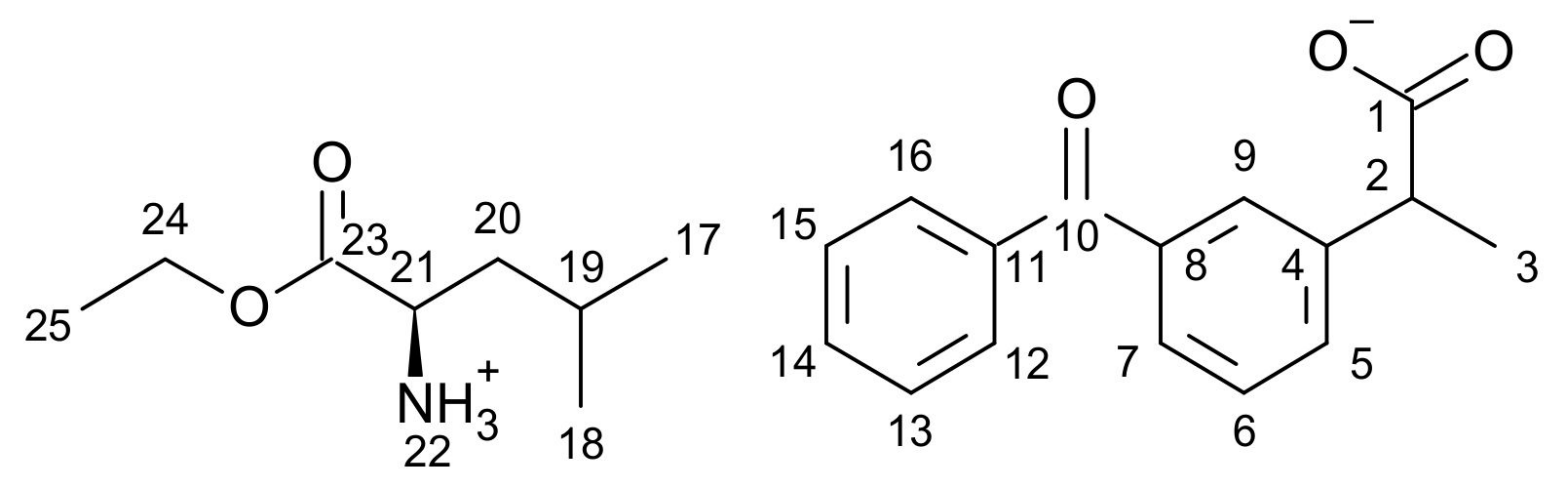
l-Leucine ethyl ester ketoprofenate.

**Scheme 3:**
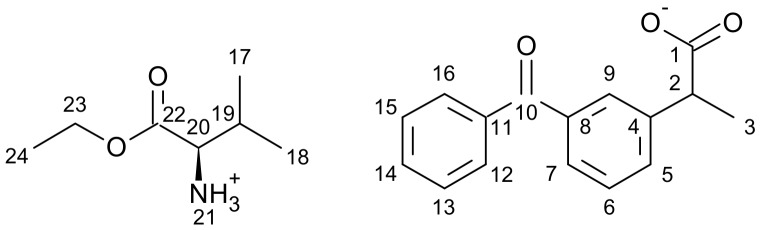
l-Valine ethyl ester ketoprofenate.

**Scheme 4:**
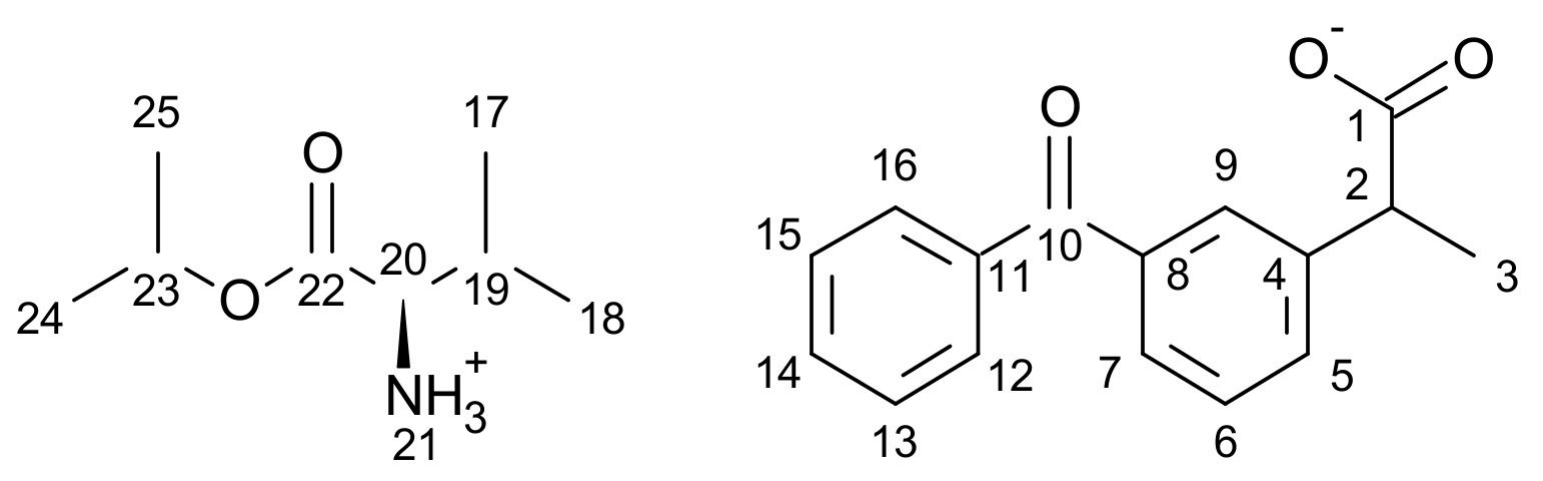
L-valine isopropyl ester ketoprofenate.

**Scheme 5:**
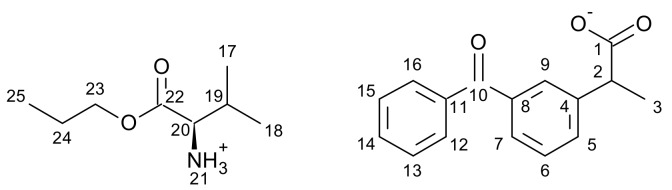
l-Valine propyl ester ketoprofenate.

**Scheme 6:**
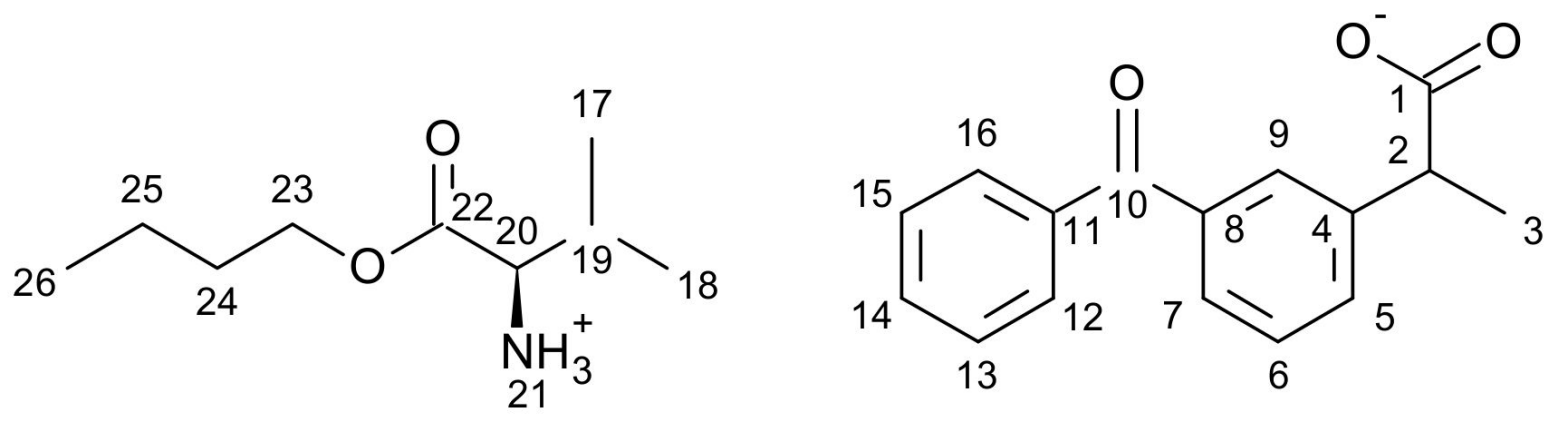
l-Valine butyl ester ketoprofenate.

**Scheme 7:**
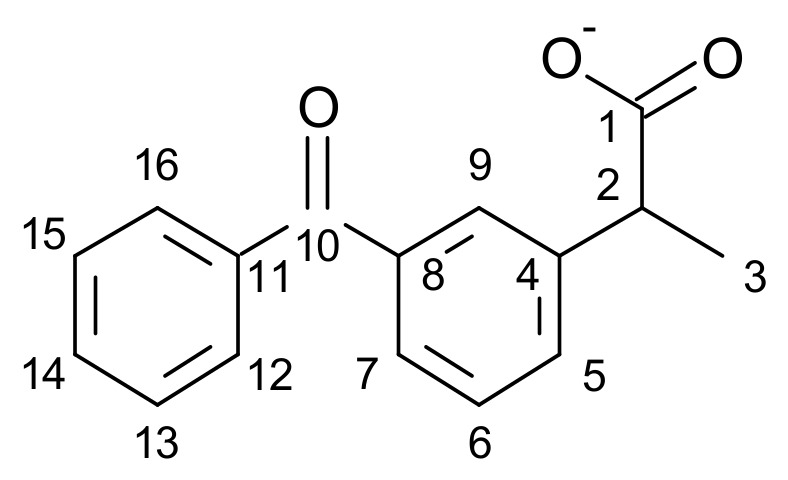
Ketoprofen.

The ketoprofen amino acid alkyl esters (Scheme 2, Scheme 3, Scheme 4, Scheme 5, Scheme 6 and Scheme 7) were synthesized using a modified three-step method (Scheme 1) [21,38]. Initially, a suspension of 2.0 g of an amino acid in 30 mL of alkyl alcohol (such as ethan-1-ol, propan-1-ol, propan-2-ol or butan-1-ol) was stirred vigorously at room temperature. Then, two molar equivalents of TMSCl were added into the reaction mixture, and the solution was stirred thoroughly at 60 °C for 24 h. The excess of TMSCl and alcohol and formed by-products were removed by evaporation at 60 °C under vacuum. The product was purified by washing with diethyl ether. As a result, the amino acid alkyl ester hydrochloride (l-AAOR·HCl) was obtained with good yield. Then, so obtained L-AAOR·HCl was reacted with the ammonia solution (mole ratio of ammonia:AAE salt = 3:1). To the reaction mixture water (5 mL) and diethyl ether (25 mL) were added, and the solution was intensively stirred for 15 min at room temperature. The organic layer was separated and dried using anhydrous Na_2_SO_4_ and then concentrated under vacuum to receive the l-AAOR. In the third step, an equimolar mixture of the synthesized l-AAOR and ketoprofen was dissolved in chloroform. The mixture was stirred thoroughly for 20 min at room temperature, and then solvents were removed by evaporation under reduced pressure. The obtained ketoprofen derivatives were white solid, and their ionization and purities were evaluated using ^1^H- and ^13^C-NMR spectroscopy.

### 3.3. Characterization of the Ketoprofen Amino Acid Alkyl-Esters [AAOR][KETO]

^1^H-NMR (400.13 MHz) and ^13^C-NMR (100.62 MHz) spectra were recorded in CDCl_3_ on a BRUKER DPX-400 Avance III HD spectrometer (Billerica, MA, USA). TMS was used as an internal standard. ATR–FTIR spectra were collected in a Thermo Fisher Scientific Nicolet FTIR 380 FTIR Spectrometer (Waltham, MA, USA) equipped with attenuated total reflectance (ATR) sampling accessory (diamond plate). Spectra were recorded in transmittance mode from 400 to 4000 cm^–1^, co–adding 16 interferograms at a resolution of 4 cm^–1^. The content of elements, i.e., hydrogen, nitrogen, carbon, and oxygen were determined by CHNS/O elemental analysis. The elemental analysis was performed using a Thermo Scientific™ FLASH 2000 CHNS/O Elemental Analyzer (Waltham, MA, USA). Thermogravimetric analysis was carried out on thermomicrobalance TG 209 F1 Libra^®^ from NETZSCH (Selb, Germany), in Al_2_O_3_ crucible. Samples between 5–10 mg were heated from 25 °C to 1000 °C with a heating rate of 10 °C min^−1^, under air atmosphere (flow rate: air—25 cm^3^ min^−1^, nitrogen (as protective gas)—10 cm^3^ min^−1^). Phase transformation temperatures were measured using a TA Instruments DSC analyzer model Q-100 (New Castle, DE, USA). Measurements were performed within the temperature range of 0 °C to 100–200 °C (depends on thermal stability), in a nitrogen atmosphere. The heating rate was 10 °C min^−1^. The sample was loaded on an aluminum pan sealed with a pinhole cap. The specific rotation [α]D_20_ measurements were determined with an AUTOPOL IV Polarimeter from Rudolph Research Analytical (Hackettstown, NJ, USA) for aqueous solutions of AAILS (c = 0.01 g cm^−3^). The measurements were performed at 20 °C.

#### 3.3.1. [l-LeuOEt][KETO]—l-Leucine Ethyl Ester Ketoprofenate

The compound was obtained according general procedure in 94.0% yield as white solid. ^1^H-NMR (400 MHz, CDCl_3_) δ in ppm: 7.71–7.81 (m, 3H, H7, H12, H16); 7.62 (d, J_9,8_ = 7.6 Hz, 1H, H9); 7.54–7.60 (m, 2H, H13, H14); 7.44–7.49 (m, 2H, H5, H15); 7.38 (t, J_6,5_ = 7.6 Hz, 1H, H6); 6.67 (s, 3H, H22); 4.12–4.18 (m, 2H, H24); 3.68–3.72 (m, 1H, H2); 3.59 (dd, J_20,19_ = 4.6 Hz, 1H, H21); 1.69–1.73 (m, 1H, H20); 1.58–1.62 (m, 1H, H19); 1.44–1.51 (m, 4H, H3, H20); 1.24 (t, J_25,24_ =7.2 Hz, 3H, H25); 0.85–0.89 (dd, J_17(18),19_ = 7.6 Hz, 6H, H17, H18); ^13^C-NMR (100 MHz, CDCl_3_) δ in ppm: 196.72 (C1); 178.79 (C23); 173.68 (C10); 142.37 (C8); 137.56 (C11); 132.43 (C4); 131.84 (C5); 130.09 (C14); 129.29 (C12); 128.57 (C16); 128.26 (C9); 128.23 (C6); 61.46 (C24); 51.79 (C21); 46.55 (C2); 42.09 (C20); 24.52 (C19); 22.58 (C18); 21.85 (C17); 18.72 (C3); 14.10 (C25); FTIR: ν (ATR): 2957; 2933; 2872; 1742; 1653; 1597; 1539; 1479; 1456; 1446; 1391; 1362; 1317; 1281; 1252; 1231; 1196; 1172; 1132; 1093; 1074; 1026; 995; 968; 932; 921; 880; 824; 780; 728; 706; 644; 607; 574; 523; 444 cm^−1^; Elemental analysis: Calc. (%) for C_24_H_31_NO_5_ (413.51 g mol^−1^) C (69.71), H (7.56), N (3.39), O (19.35), Found C (69.82), H (7.53), N (3.37), O (19.33).

#### 3.3.2. [l-ValOEt][KETO]—l-Valine Ethyl Ester Ketoprofenate

The compound was obtained according general procedure in 94.0% yield as white solid. ^1^H-NMR (400 MHz, CDCl_3_) δ in ppm: 7.75–7.81 (m, 3H, H7, H12, H16); 7.64 (d, J_9,8_ = 7.6 Hz, 1H, H9); 7.53–7.60 (m, 2H, H13, H14); 7.48 (t, J_6,5_ = 7.3 Hz, 2H, H5, H15); 7.40 (t, J_6,5_ = 7.7 Hz, 1H, H6); 5.75 (s, 3H, H21); 4.02–4.26 (m, 2H, H23); 3.68–3.72 (m, 1H, H2); 3.44 (d, J_20,19_ = 4.6 Hz, 1H, H20); 1.98–2.16 (m, 1H, H19); 1.49 (d, J_3,2_ = 7.1 Hz, 3H, H3); 1.25 (t, J_24,23_ = 7.2 Hz, 3H, H24); 0.88–0.95 (m, 6H, H17, H18); ^13^C-NMR (100 MHz, CDCl_3_) δ in ppm: 196.65 (C1); 178.49 (22); 173.58 (C10); 141.64 (C8); 137.72 (C11); 137.54 (C4); 132.45 (C5); 131.76 (C14); 130.10 (C12/C16); 129.31 (C9); 128.79 (C6); 128.37 (C7); 128.28 (C13); 61.17 (C23); 58.95 (C20); 46.00 (C2); 31.39 (C19); 18.72 (C18), 18.52 (C17); 17.29 (C3); 14.21 (C24); FTIR: ν (ATR): 2968; 2929; 2876; 2610; 1741; 1651; 1596; 1574; 1517; 1481; 1463; 1446; 1387; 1361; 1316; 1280; 1257; 1218; 1204; 1180; 1173; 1135; 1107; 1074; 1065; 1053; 1015; 996; 966; 952; 878; 859; 778; 718; 705; 642 cm^−1^; Elemental analysis: Calc. (%) for C_23_H_29_NO_5_ (399.48 g mol^−1^) C (69.15), H (7.32), N (3.51), O (20.03), Found C (69.20), H (7.30), N (3.55), O (20.10).

#### 3.3.3. [l-ValOiPr][KETO]—l-Valine Isopropyl Ester Ketoprofenate

The compound was obtained according general procedure in 90.0% yield as white solid. ^1^H-NMR (400 MHz, CDCl_3_) δ in ppm: 7.73–7.83 (m, 3H, H7, H12, H16); 7.61–7.67 (dt, 1H, H9); 7.52–7.60 (m, 2H, H13, H14); 7.47 (t, J_6,5_ = 7.6 Hz, 2H, H5, H15); 7.39 (t, J_6,5_ = 7.7 Hz, 1H, H6); 5.63 (s, 3H, H21); 4.95–5.12 (m, 2H, H23); 3.66–3.79 (m, 1H, H2); 3.40 (d, J_20,19_ = 4.6 Hz, 1H, H20); 2.03–2.12 (m, 1H, H19); 1.49 (d, J_3,2_ = 7.2 Hz, 3H, H3); 1.24 (d, J_24,23_ = 7.1 Hz, 6H, H24, 25); 0.87–0.95 (dd, 6H, H17, H18); ^13^C-NMR (100 MHz, CDCl_3_) δ in ppm: 196.70 (C1); 178.48 (C22); 141.95 (C10); 137.65 (C8); 137.54 (C11); 132.43 (C4); 131.79 (C5); 130.10 (C14); 129.33 (C12/C16); 129.31 (C9); 128.69 (C6); 128.32 (C7); 128.27(C13); 68.90 (C23); 58.90 (C20); 46.21 (C2); 31.30 (C19); 21.76 (C18); 18.65 (C17); 18.60 (C3); 17.26 (C24/C25); FTIR: ν (ATR): 2976; 2933; 2877; 1738; 1660; 1597; 1577; 1558; 1448; 1387; 1358; 1318; 1283; 1233; 1180; 1144; 1105; 1075; 999; 954; 915; 880; 822; 775; 721; 705; 643 cm^−1^; Elemental analysis: Calc. (%) for C_24_H_31_NO_5_ (413.51 g mol^−1^) C (69.71), H (7.57), N (3.39), O (19.35), Found C (69.75), H (7.56), N (3.38), O (19.34).

#### 3.3.4. [l-ValOPr][KETO]—l-Valine Propyl Ester Ketoprofenate

The compound was obtained according general procedure in 93.0% yield as white solid. ^1^H-NMR (400 MHz, CDCl_3_) δ in ppm: 7.74–7.82 (m, 3H, H7, H12, H16); 7.63–7.68 (dt, 1H, H9); 7.52–7.60 (m, 2H, H13, H14); 7.47 (t, J_6,5_ = 7.6 Hz, 2H, H5, H15); 7.41 (t, J_6,5_ = 7.7 Hz, 1H, H6); 4.85 (s, 3H, H21); 4.03–4.12 (m, 2H, H23); 3.72–3.79 (m, 1H, H2); 3.42 (d, J_20,19_ = 4.7 Hz, 1H, H20); 2.03–2.13 (m, 1H, H19); 1.61–1.69 (m, 2H, H24); 1.51 (d, J_3,2_ = 7.1 Hz, 3H, H3); 0.89–0.97 (m, 6H, H17, H18, H25); ^13^C-NMR (100 MHz, CDCl_3_) δ in ppm: 196.64 (C1); 178.35 (C10); 174.20 (C22); 141.41 (C8); 137.75 (C11); 137.52 (C4); 132.47 (C5); 131.73 (C14); 130.10 (C12); 129.32 (C9); 128.88 (C6); 128.42 (C7); 128.29 (C13/C15); 66.71 (C23); 59.14 (C20); 45.81 (C2); 31.59 (C19); 21.95 (C24); 18.88 (C18); 18.46 (C17); 17.23 (C3); 10.40 (C25); FTIR: ν (ATR): 2969; 2935; 2879; 1742; 1659; 1597; 1578; 1462; 1448; 1389; 1358; 1318; 1283; 1222; 1179; 1140; 1059; 999; 955; 929; 910; 881; 777; 721; 705; 643; cm^−1^; Elemental analysis: Calc. (%) for C_24_H_31_NO_5_ (413.51 g mol^−1^) C (69.71), H (7.57), N (3.39), O (19.35), Found C (69.74), H (7.55), N (3.37), O (19.33).

#### 3.3.5. [l-ValOBu][KETO]—l-Valine Butyl Ester Ketoprofenate

Compound was obtained according general procedure in 95.0% yield as white solid. ^1^H-NMR (400 MHz, CDCl_3_) δ in ppm: 7.75–7.82 (m, 3H, H7,H12, H16); 7.61–7.66 (dt, 1H, H9); 7.53–7.60 (m, 2H, H13, H14); 7.47 (t, J_6,5_ = 7.6 Hz, 2H, H5, H15); 7.39 (t, J_6,5_ = 7.7 Hz, 1H, H6); 5.62 (s, 3H, H21); 4.05–4.16 (m, 2H, H23); 3.68–3.76 (m, 1H, H2); 3.44 (d, J_20,19_ = 4.5 Hz, 1H, H20); 2.04–2.12 (m, 1H, H19); 1.56–1.64 (m, 2H, H24); 1.48 (d, J_3,2_ = 7.2 Hz, 3H, H3); 0.87–0.95 (m, 9H, H17, H18, H25, H26); ^13^C-NMR (100 MHz, CDCl_3_) δ in ppm: 196.70 (C1); 178.52 (C10); 173.51 (C22); 141.94 (C8); 137.65 (C11); 137.53 (C4); 132.45 (C5); 131.80 (C14); 130.10 (C12); 129.31 (C9); 128.70 (C6); 128.32 (C7); 128.27 (C13/C15); 65.10 (C23); 58.91 (C20); 46.21 (C2); 31.31 (C19); 30.56 (C24); 19.09 (C25); 18.60 (C18); 18.60 (C17); 17.32 (C3); 13.65 (C26); FTIR: ν (ATR): 2964; 2934; 2875; 1742; 1660; 1597; 1578; 1462; 1448; 1389; 1358; 1318; 1283; 1242; 1219; 1179; 1140; 1062; 1021; 999; 954; 881; 779; 721; 705; 643 cm^−1^; Elemental analysis: Calc. (%) for C_25_H_33_NO_5_ (427.53 g mol^−1^) C (70.23), H (7.78), N (3.27), O (18.71), Found C (70.25), H (7.80), N (3.25), O (19.69).

#### 3.3.6. Ketoprofen

^1^H-NMR (400 MHz, CDCl3) δ in ppm: 7.67–7.74 (m, 3H, H9, H16, H12); 7.59–7.61 (dt, 1H, H7); 7.44–7.54 (m, 2H, H4, H5); 7.30–7.42; (m, 3H, H); 3.69–3.79 (m, 1H, H); 1.46 (d, J_2,1_ = 7.1 Hz, 3H, H1); ^13^C-NMR (100 MHz, CDCl_3_) δ in ppm: 196.56 (C10); 180.24 (1); 140.12 (C8); 137.93 (C11); 137.41 (C4); 132.60 (C5); 131.71 (C14); 130.14 (C12/C16); 129.39 (C9); 129.31 (C6); 128.63 (C7); 128,34 (C13/C15); 45.25 (C2); 18.13 (C3); FTIR: ν (ATR): 2978; 2926; 1697; 1655; 1598; 1576; 1457; 1419; 1370; 1319; 1308; 1285; 1227; 1195; 1175; 1134; 1178; 1063; 1061; 968; 928; 916; 866; 717; 703; 691; 643; 614 cm^−1^; Elemental analysis: Calc. (%) for C_16_H_14_O_3_ (254.28 g mol^−1^) C (75.58), H (5.55), N (0.00), O (18.88), Found C (75.56), H (5.56), N (0.00), O (18.88).

### 3.4. Fluorescence Quenching Measurements

All fluorescence measurements were carried out on Perkin Elmer LS 55 fluorimeter equipped with a 1.0 cm quartz cuvette. 

The experiments were carried out in sodium phosphate buffer (pH 7.4, 50 mM) at 25 °C, the BSA concentration was kept constant (2 μM) and the concentration of the ketoprofen and [AAOR][KETO] were varied from 2 to 60 μM. The fluorescence spectra were recorded in the range from 295 to 550 nm by exciting BSA at 280 nm using a slit width of 10/10 nm. BSA-ILs incubated 1 hour prior to the measurements.

The measurements of UV-spectrum of ketoprofen and ILs at fixed concentrations were performed on Evolution 300 Thermo Scientific equipped with a Peltier temperature control accessory with the highest resolution (1 nm) using matched 1 cm path length quartz cuvettes.

### 3.5. Fourier Transformed Infrared Spectroscopy

FTIR spectra of the BSA (20 g L^−1^, 0.3 mM) dissolved in sodium phosphate buffer (pH 7.4, 50 mM) and its complexes with 0.3 mM ketoprofen or ILs (molar ratio 1:1) were recorded on Bruker Tensor 27 spectrometer, equipped with a detector of deuterated triglycine sulphate (DTGS). The FTIR spectra were collected by direct deposition of the samples on attenuated total reflectance (ATR) element (diamond crystal) in frequency region 4000–600 cm^−1^ (ATR) with 128 scannings and at a resolution of 1 cm^−1^. The spectra of the proteins were referenced to the respective spectra of 50 mM sodium phosphate buffer, pH 7.4 containing the same amount of the corresponding IL. 

Then the ATR-FTIR spectra were Fourier deconvoluted by Opus software version 5.5 using a bandwidth of 12 cm^−1^, 2.9 resolution enhancement factor, and Lorentzian lineshape. Second derivative spectra were obtained using the Savitzky-Golay algorithm based on 17 smoothing points. Then, the relative contribution of each band component of the Amide I band was determined by curve fitting following the procedure of the OPUS program. In the fitting, the number of components and the initial values of their position were set as determined from the second derivative spectra. The initial bandwidth of all components was set to 12 cm^−1^ and the components were approximated by mixed Lorentzian/Gaussian functions. The curve-fitting was performed according to the Local Least Squares algorithm. The assignment of the Amide I band positions to the secondary structure was done according to [36].

### 3.6. Cell Cultures Conditions

Murine macrophages RAW 264.7 were cultured in DMEM high glucose medium containing L-Glutamine, antibiotic mixture B and 10% fetal bovine serum. The cells were cultivated at a humified atmosphere, 37 °C, and 5% CO_2_.

For the experimental procedures, cells were seeded in a sterile 96-well plate at 1 × 10^4^ cells per well and incubated for 24 h at 37 °C and 5% CO_2_ for obtaining adherent cell cultures and good cell spreading. Then, the cells were incubated for an additional 24 h with ketoprofen or [AAOR][KETO] at concentrations 1, 10, and 100 μM. Control experiments with non-treated cells, treated with 1% DMSO (the solvent used to prepare the stock solution of the samples) and 100 μg NaF and 1% DMSO were performed. 

### 3.7. Cell Viability Assay

The cell viability of RAW 264.7 cells in the presence of the ILs and ketoprofen was applied a colorimetric assay using an Alamar Blue reagent. The reagent was diluted with DMEM media (1:10), an aliquot of 10 μL of the solution was added to each well and incubated for 3h at 37 °C under a flow of 5% CO_2_. 

The measurements were carried out at an excitation wavelength of 544 nm and an emission wavelength of 590 nm with a 96-well plate reader FLUOstar Galaxy (Tecan BMG Labtechnologies, Ortenberg, Germany). The survival of the cells, treated with different concentrations of the tested compounds, was presented in percentages from the control (non-treated cells). 

The values are presented as mean ± standard deviation of three independent experiments. The values were significantly different if the *p*-value was < 0.05.

## 4. Conclusions

Five novel ionic liquids containing ketoprofen anion were synthesized. The compounds were safe for the immune cells in the tested concentration range. The affinity of the ILs to serum albumin is within the range of the estimated binding constants (KA) are in order of 10^5^ L mol^−1^, which is indicative of strong drug or IL-BSA interactions. The compounds have the potential to be the basis of novel formulations.

## Figures and Tables

**Table 1:** The melting points and thermal stability of ketoprofen and its amino acid ester derivatives.

Compound	Colour	T_m_ (°C)	T_onset_ (°C)	[α]_λ_^T^	[M]_λ_^T^
[l-LeuOEt][KETO]	white	94.23	95.7	+7.739	32.002
[l-ValOEt][KETO]	white	63.55	100.4	+4.679	+18.690
[l-ValOiPr][KETO]	white	58.59	69.4	+8.083	+33.426
[l-ValOPr][KETO]	white	60.09	52.3	+5.455	+22.559
[l-ValOBu][KETO]	white	58.75	101.1	+4.167	+17.819
KETO	white	92.17	265.4	0	0

T_m_—melting point; T_onset_—the onset of the thermal degradation; [α]_λ_^T^—specific rotation; [M]_λ_^T^—molar specific rotation.

**Table 2:** Solubility in water and organic solvents of ketoprofen and its amino acids derivatives at 25 °C.

Compound	Water (63.1)	Ethanol (51.9)	Chloroform (39.1)	Ethyl-Acetate (38.1)	Diethyl-Ether (34.5)	Toluene (33.9)	*n*-Hexane (31.0)
[l-LeuOEt][KETO]	−	+/−	+/−	−	−	+	−
[l-ValOEt][KETO]	−	+	+	−	+/−	−	−
[l-ValOiPr][KETO]	−	+	+/−	−	+	+	−
[l-ValOPr][KETO]	−	+	+	+	+/−	+	−
[l-ValOBu][KETO]	−	+	+	−	−	+	−
KETO	−	+	+	+/−	+	−	−

Solvents were ranked with decreasing value of empirical solvent polarityarameters, E_T_(30) [23] (“+”: soluble >100 g L^−1^; “+/−”: partially soluble 33–100 g L^−1^; “−”: insoluble <33 g L^−1^) at the temperature 25 °C by modified Vogel’s method [30].

**Table 3:** Stern–Volmer quenching constants (*K_sv_*) and bimolecular quenching rate constant (*K_q_*) of binding of ketoprofen and the ketoprofen-based ILs with BSA at 25 °C.

Compound	*K_sv_* (10^4^, L mol^−1^)	*k_q_* (10^12^, L mol^−1^ s^−1^)	R
[l-LeuOEt][KETO]	8.3	8.3	0.9900
[l-ValOEt][KETO]	6.3	6.3	0.9948
[l-ValOiPr][KETO]	7.1	7.1	0.9981
[l-ValOPr][KETO]	7.1	7.1	0.9958
[l-ValOBu][KETO]	7.3	7.3	0.9969
KETO	8.4	8.4	0.9953

R—correlation coefficient.

**Table 4:** Binding constants (*K_A_*) and binding sites (*n*) of binding of ketoprofen and the ketoprofen-based ILs with BSA at 25 °C.

Compound	*K_A_* (10^5^, L mol^−1^)	N	R
[l-LeuOEt][KETO]	7.1	1.2	0.9972
[l-ValOEt][KETO]	8.2	1.2	0.9934
[l-ValOiPr][KETO]	1.5	1.1	0.9985
[l-ValOPr][KETO]	2.3	1.1	0.9924
[l-ValOBu][KETO]	7.5	1.2	0.9988
KETO	2.6	1.1	0.9954

R—correlation coefficient.

**Table 5:** Steady-state FRET parameters of the BSA-[AAOR] [KETO] interactions.

System	*R_o_* (nm)	E_FRET_	*r_o_* (nm)
BSA-[l-LeuOEt][KETO]	3.30	0.0246	6.14
BSA-[l-ValOEt][KETO]	3.43	0.1830	4.68
BSA-[l-ValOiPr][KETO]	3.38	0.0625	5.42
BSA-[l-ValOPr][KETO]	3.46	0.1770	4.74
BSA-[l-ValOBu][KETO]	3.32	0.0520	5.48
BSA-KETO	3.37	0.0630	5.40

[BSA] = [Quencher] = 2 μM; T = 25 °C.

**Table 6:** FTIR analysis of the Amide I band of BSA-[KETO][AAOR] complexes in phosphate buffer (pH 7.4, 50 mM).

Assignment of the Secondary Structure Components [36]	α-Helices		β-Structures		Unordered Structures		Aromatic Residues/Aggregated Strands/Antiparallel β-Sheets	
	Band Position (cm^−1^)	Relative Area (%)	Band Position (cm^−1^)	Relative Area (%)	Band Position (cm^−1^)	Relative Area (%)	Band Position (cm^−1^)	Relative Area (%)
native BSA	1655	47.3	1629; 1674	11.6; 12.9	1642	21	1617	7.1
BSA-KETO	1655	46.2	1636; 1673	36.2; 12.4	-	-	1618	5.2
BSA-[l-LeuOEt][KETO]	1655	36.7	1628; 1673	13.5; 13.7	1642	21.5	1615; 1684; 1696	11.6; 1.9; 1.0
BSA-[l-ValOEt][KETO]	1656	28.8	1628; 1674	22.4; 15.0	1643	17.25	1607; 1698	14.1; 2.4
BSA-[l-ValOiPr][KETO]	1658	23.8	1626; 1638; 1674	15.5; 11.2; 11.8	1648	11.7	1604; 1605; 1687	2.0; 23.3; 1.0
BSA-[l-ValOPr][KETO]	1658	26.9	1631; 1673	19.5; 13.6	1646	29.2	1616	11.0
BSA-[l-ValOBu][KETO]	1656	27.8	1640; 1675	31.0; 11.8	-	-	1620; 1693	26.3; 1.0

## Supplementary Materials

The following are available online, Figure S1: ^1^H-NMR spectra of ketoprofen and its derivatives (in the red square, the protonated amino group is marked)—from the top: [l-LeuOEt][KETO], [l-ValOEt][KETO], [l-ValOiPr][KETO], [l-ValOPr][KETO], [l-ValOBu][KETO] and [KETO], Figure S2: 13C-NMR spectra of ketoprofen and its derivatives—from the top: [l-LeuOEt][KETO], [l-ValOEt][KETO], [l-ValOiPr][KETO], [l-ValOPr][KETO], [l-ValOBu][KETO] and [KETO], Figure S3: ^1^H-NMR spectra of l-leucine ethyl ester ketoprofenate, Figure S4: ^13^C-NMR spectra of l-leucine ethyl ester ketoprofenate, Figure S5: ^1^H-NMR spectra of l-valine ethyl ester ketoprofenate, Figure S6: ^13^C-NMR spectra of l-valine ethyl ester ketoprofenate, Figure S7: ^1^H-NMR spectra of l-valine isopropyl ester ketoprofenate, Figure S8: ^13^C-NMR spectra of l-valine isopropyl ester ketoprofenate, Figure S9: ^1^H-NMR spectra of l-valine propyl ester ketoprofenate, Figure S10: ^13^C-NMR spectra of l-valine propyl ester ketoprofenate, Figure S11: ^1^H-NMR spectra of l-valine butyl ester ketoprofenate, Figure S12: ^13^C-NMR spectra of l-valine butyl ester ketoprofenate, Figure S13: ^1^H-NMR spectra of ketoprofen, Figure S14: ^13^C-NMR spectra of ketoprofen, Figure S15: FTIR spectra of [l-LeuOEt][KETO], Figure S16: FTIR spectra of [l-ValOEt][KETO], Figure S17: FTIR spectra of [l-ValOiPr][KETO]; Figure S18: FTIR spectra of [l-ValOPr][KETO], Figure S19: FTIR spectra of [l-ValOBu][KETO]; Figure S20: FTIR spectra of [KETO], Figure S21:The TG, DTG and c-DTA curves of [l-LeuOEt][KETO], Figure S22:The TG, DTG and c-DTA curves of [l-ValOEt][KETO]; Figure S23: The TG, DTG and c-DTA curves of [l-ValOiPr][KETO], Figure S24:The TG, DTG and c-DTA curves of [l-ValOPr][KETO], Figure S25: The TG, DTG and c-DTA curves of [l-ValOBu][KETO], Figure S26:The TG, DTG and c-DTA curves of [KETO], Figure S27: The DSC curves of [l-LeuOEt][KETO], Figure S28: The DSC curves of [l-ValOEt][KETO], Figure S29: The DSC curves of [l-ValOiPr][KETO], Figure S30: The DSC curves of [l-ValOPr][KETO], Figure S31: The DSC curves of [l-ValOBu][KETO], Figure S32: The DSC curves of [KETO], Figure S33: The plot of log (Fo − F)/F vs log. [Q] at 25 °C, where Q is ketoprofen (A); [l-LeuOEt][KETO] (B); [l-ValOEt][KETO] (C); [l-ValOiPr][KETO] (D) [l-ValOPr][KETO] (E) and [l-ValOBu][KETO] (F), Figure S34: The original (a), second derivative and deconvoluted ATR-FTIR spectra in Amide I region of native BSA in PBS buffer (pH 7.4, 50 mM) at concentration of 20 mg L^−1^, Figure S35: The original (a), second derivative and deconvoluted ATR-FTIR spectra in Amide I and Amide II region of native BSA-ketoprofen (1:1) in PBS buffer (pH 7.4, 50 mM), Figure S36: The original (a), second derivative and deconvoluted ATR-FTIR spectra in Amide I and Amide II region of native BSA-[l-LeuOEt][KETO] (1:1) in PBS buffer (pH 7.4, 50 mM), Figure S37: The original (a), second derivative and deconvoluted ATR-FTIR spectra in Amide I and Amide II region of native BSA-[l-ValOEt][KETO] (1:1) in PBS buffer (pH 7.4, 50 mM), Figure S38: The original (a), second derivative and deconvoluted ATR-FTIR spectra in Amide I and Amide II region of native BSA-[l-ValOiPr][KETO] (1:1) in PBS buffer (pH 7.4, 50 mM), Figure S39: The original (a), second derivative and deconvoluted ATR-FTIR spectra in Amide I and Amide II region of native BSA-[l-ValOPr][KETO] (1:1) in PBS buffer (pH 7.4, 50 mM), Figure S40: The original (a), second derivative and deconvoluted ATR-FTIR spectra in Amide I and Amide II region of native BSA-[l-ValOBu][KETO] (1:1) in PBS buffer (pH 7.4, 50 mM).
