# Supplementary material for: Ketoprofen-Based Ionic Liquids: Synthesis and Interactions with Bovine Serum Albumin
Source: Molecules. 2019 Dec 25;25(1):90. doi: 10.3390/molecules25010090 (PMC6983093; doi:10.3390/molecules25010090)
Supplement: Supplementary file 1 [file molecules-25-00090-s001.pdf]

Supplementary materials

Ketoprofen-based ionic liquids: synthesis and interactions with bovine serum albumin

Paula Ossowicz[1], Proletina Kardaleva[2], Maya Guncheva[2,*], Joanna Klebeko[1], Ewelina Świątek[1], Ewa Janus[1], Denitsa Yancheva[2], Ivan Angelov[2]

[1] West Pomeranian University of Technology, Faculty of Chemical Technology and Engineering, Department of Chemical Organic Technology and Polymeric Materials, Piastów Ave. 42, 71-065 Szczecin, Poland

[2]Institute of Organic Chemistry with Centre of Phytochemistry, Bulgarian Academy of Sciences, Acad. G. Bonchev Str. Bl. 9, 1113 Sofia, Bulgaria

* corresponding author: maiag@orgchm.bas.bg (M. Guncheva)

CHARACTERIZATION OF KETOPROFEN AMINO ACID ESTER DERIVATIVES

Table of Contents:

1. NMR spectra of ketoprofen and its derivatives S2
2. FT IR spectra of ketoprofen and its derivatives S16
3. Thermogravimetric analysis of ketoprofen and its derivatives S22
4. DSC analysis of ketoprofen and its derivatives S28
5. The plot of log (Fo-F)/F vs log. [Q] at 25ºC of ketoprofen and its derivatives S34
6. The original, second derivative and deconvoluted ATR-FTIR spectra in Amide I and Amide II region of native BSA, native BSA-ketoprofen and its derivatives S36

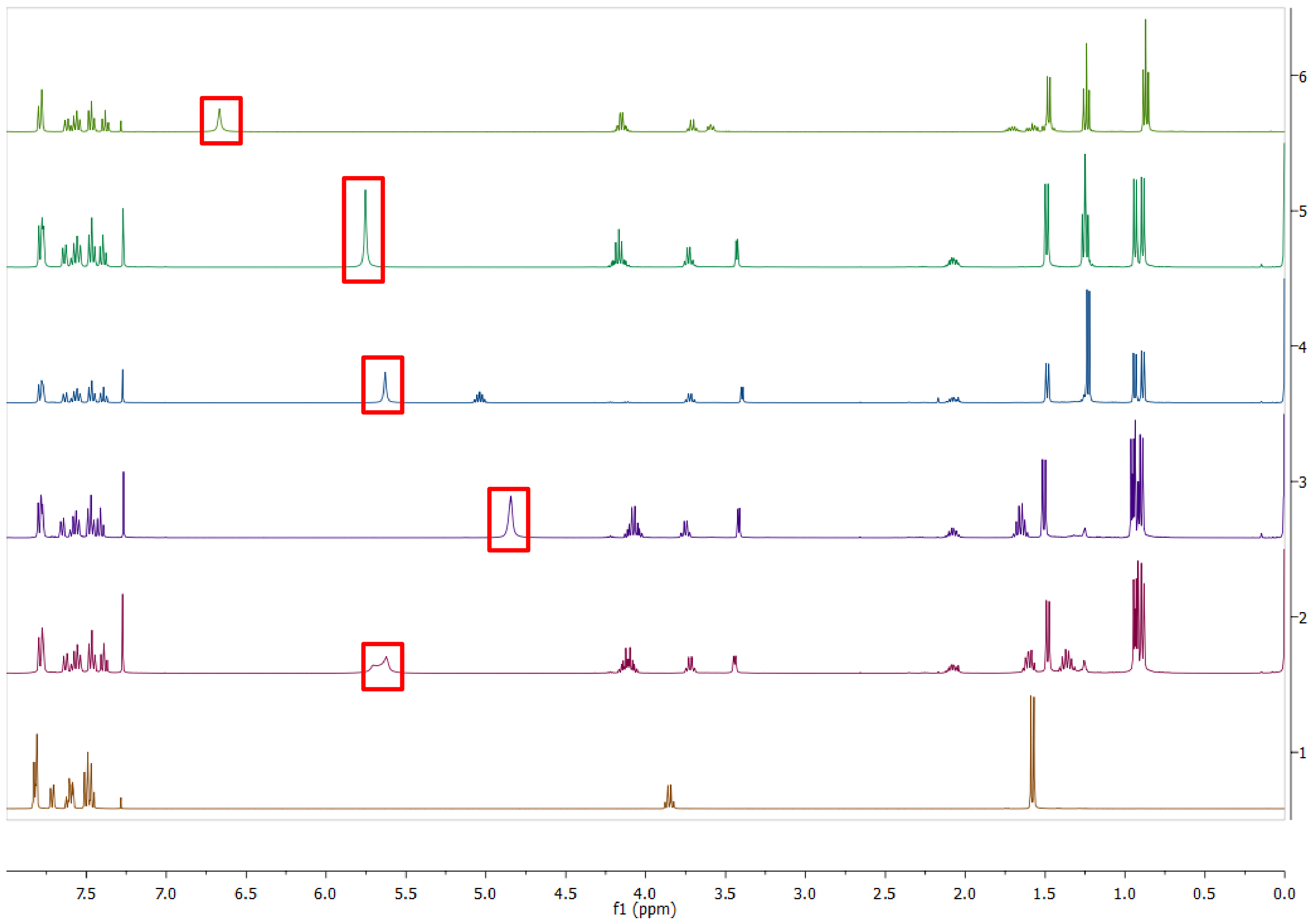

Fig. S1. ^{1}H NMR spectra of ketoprofen and its derivatives (in the red square, the protonated amino group is marked) – from the top: [L-LeuOEt][KETO], [L-ValOEt][KETO], [L-ValOiPr][KETO], [L-ValOPr][KETO], [L-ValOBu][KETO] and [KETO]

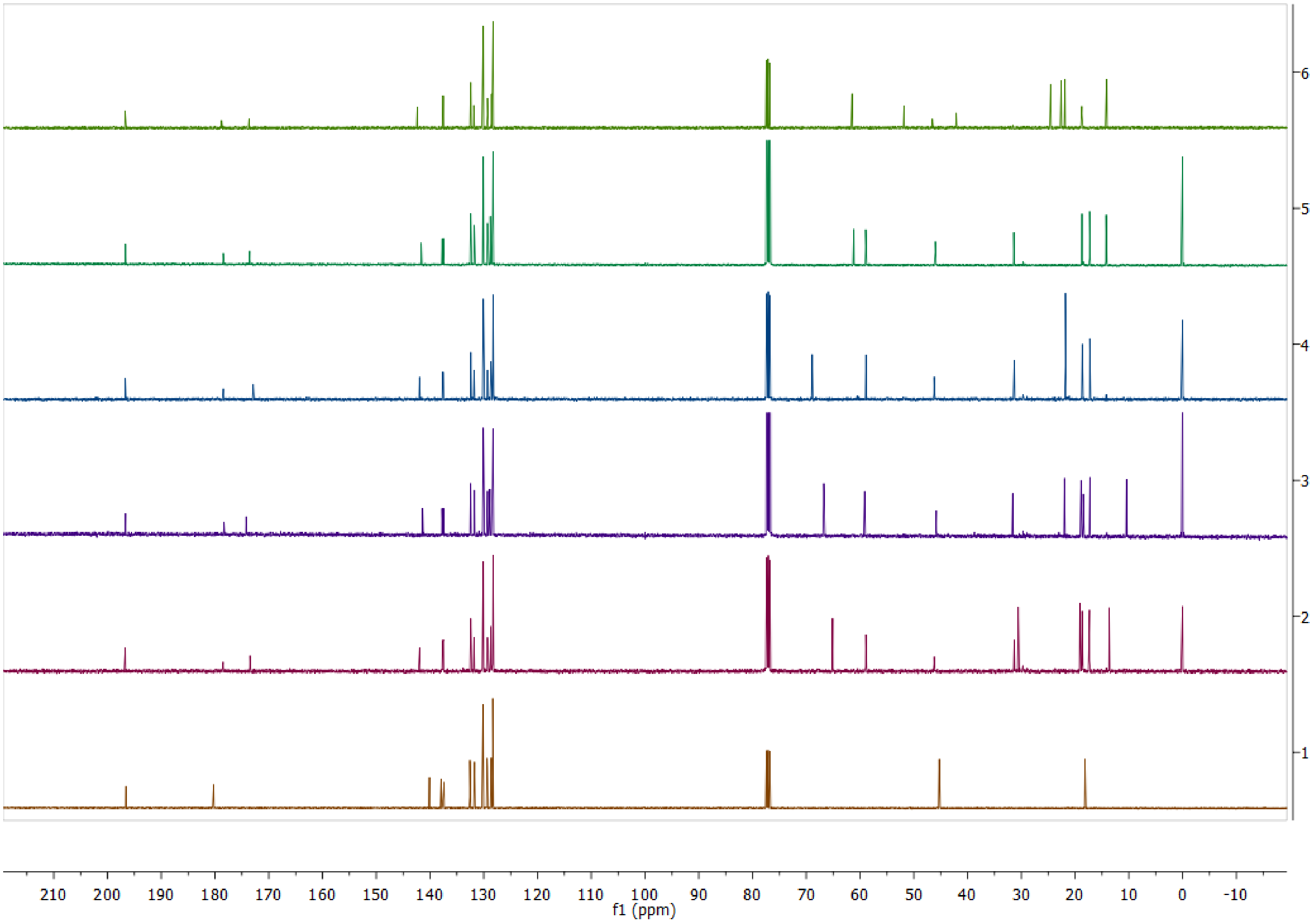

Fig. S2. ^{13}C NMR spectra of ketoprofen and its derivatives – from the top: [L-LeuOEt][KETO], [L-ValOEt][KETO], [L-ValOiPr][KETO], [L-ValOPr][KETO], [L-ValOBu][KETO] and [KETO]

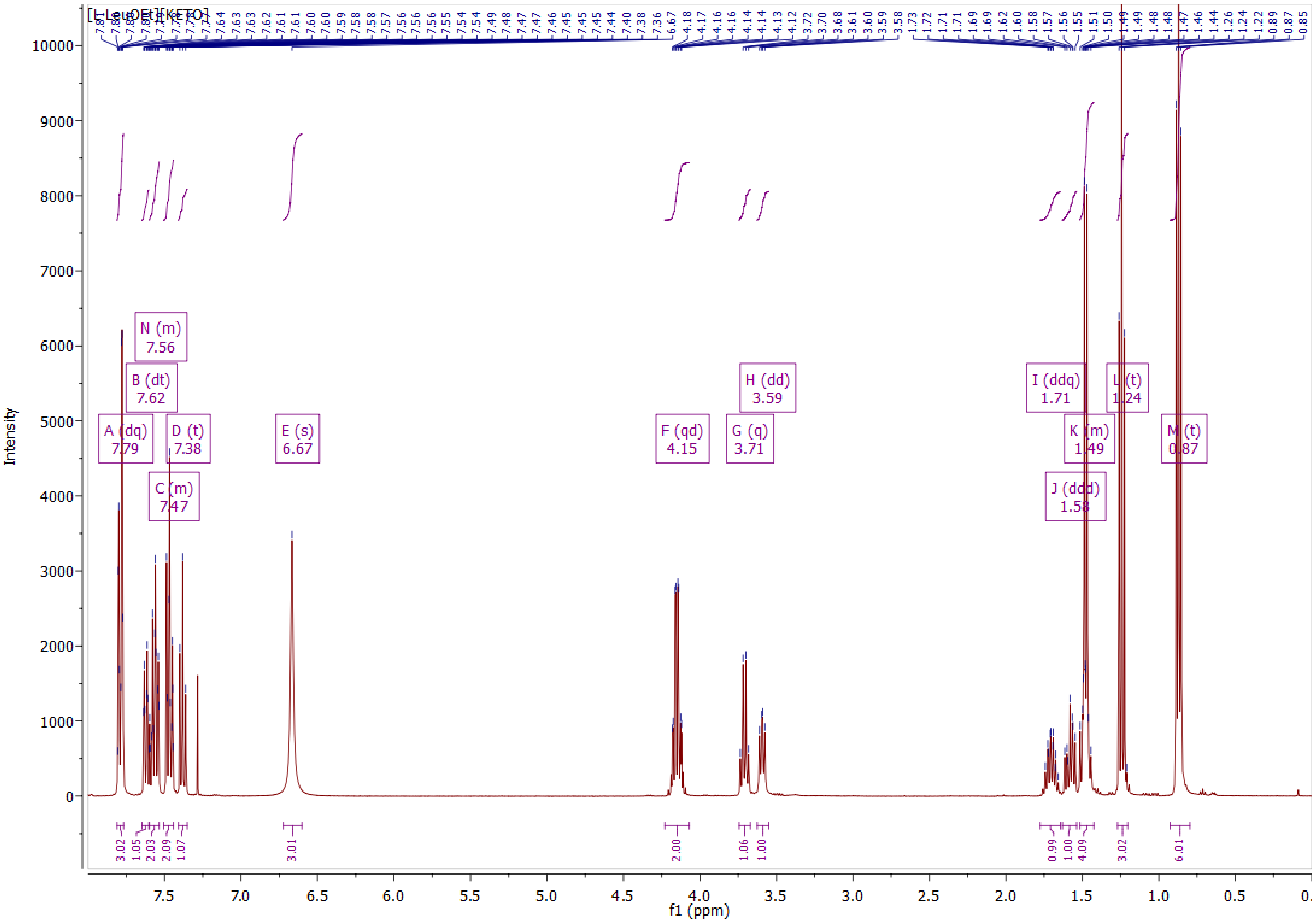

Fig.S3. ^{1}H NMR spectra of L-leucine ethyl ester ketoprofenate

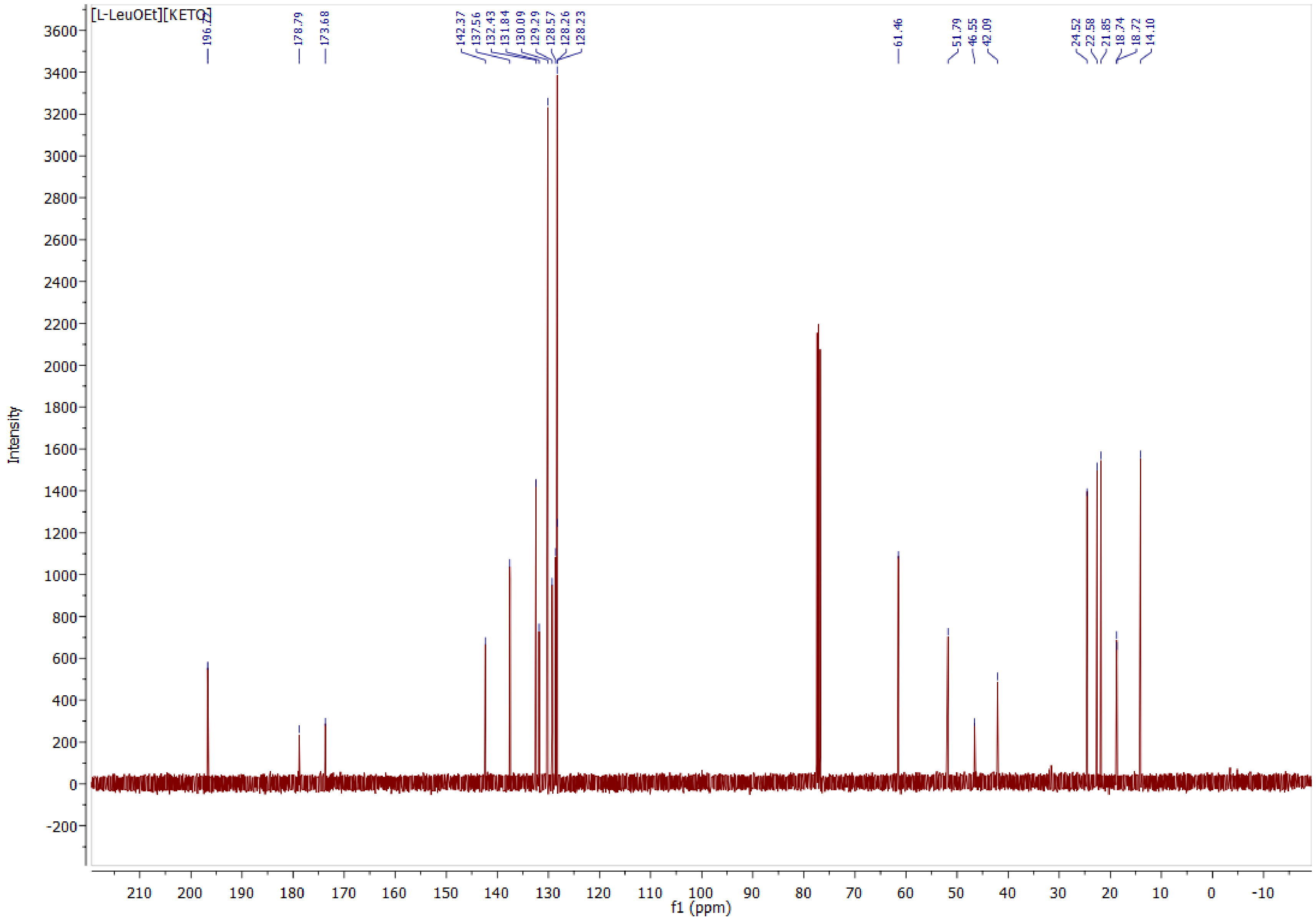

Fig. S4. ^{13}C NMR spectra of L-leucine ethyl ester ketoprofenate.

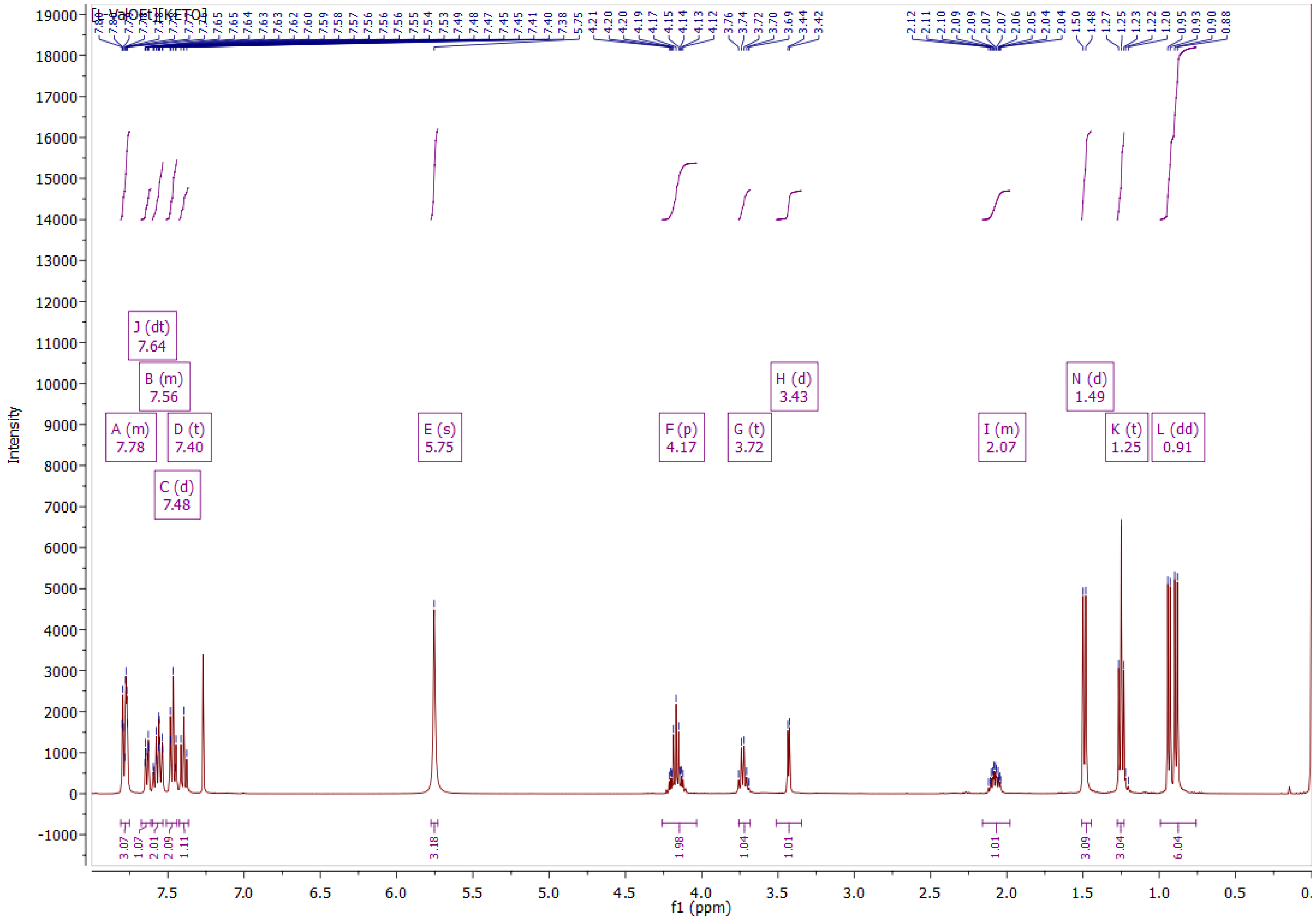

Fig.S5. 1H NMR spectra of L-valine ethyl ester ketoprofenate

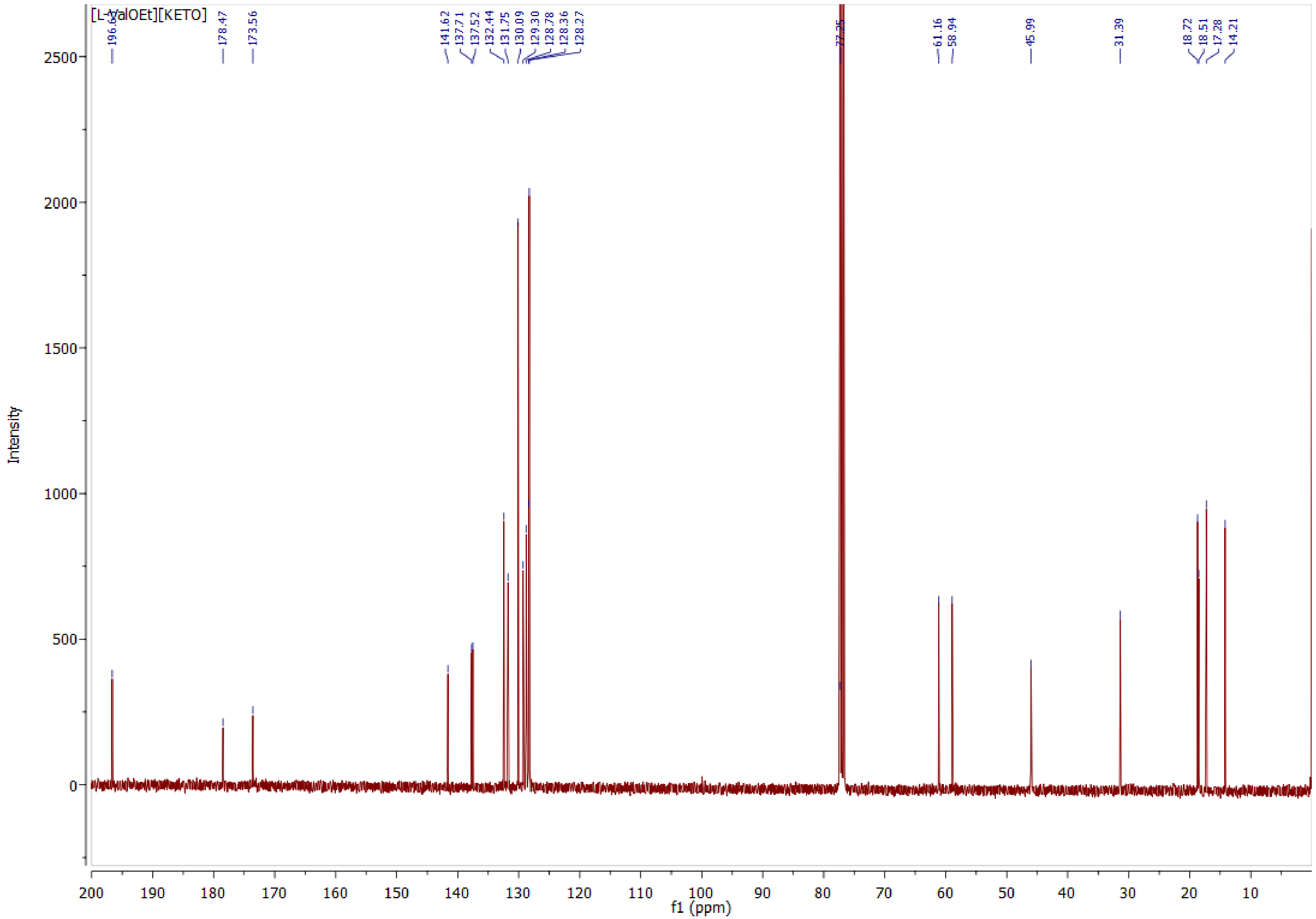

Fig. S6. ^{13}C NMR spectra of L-valine ethyl ester ketoprofenate

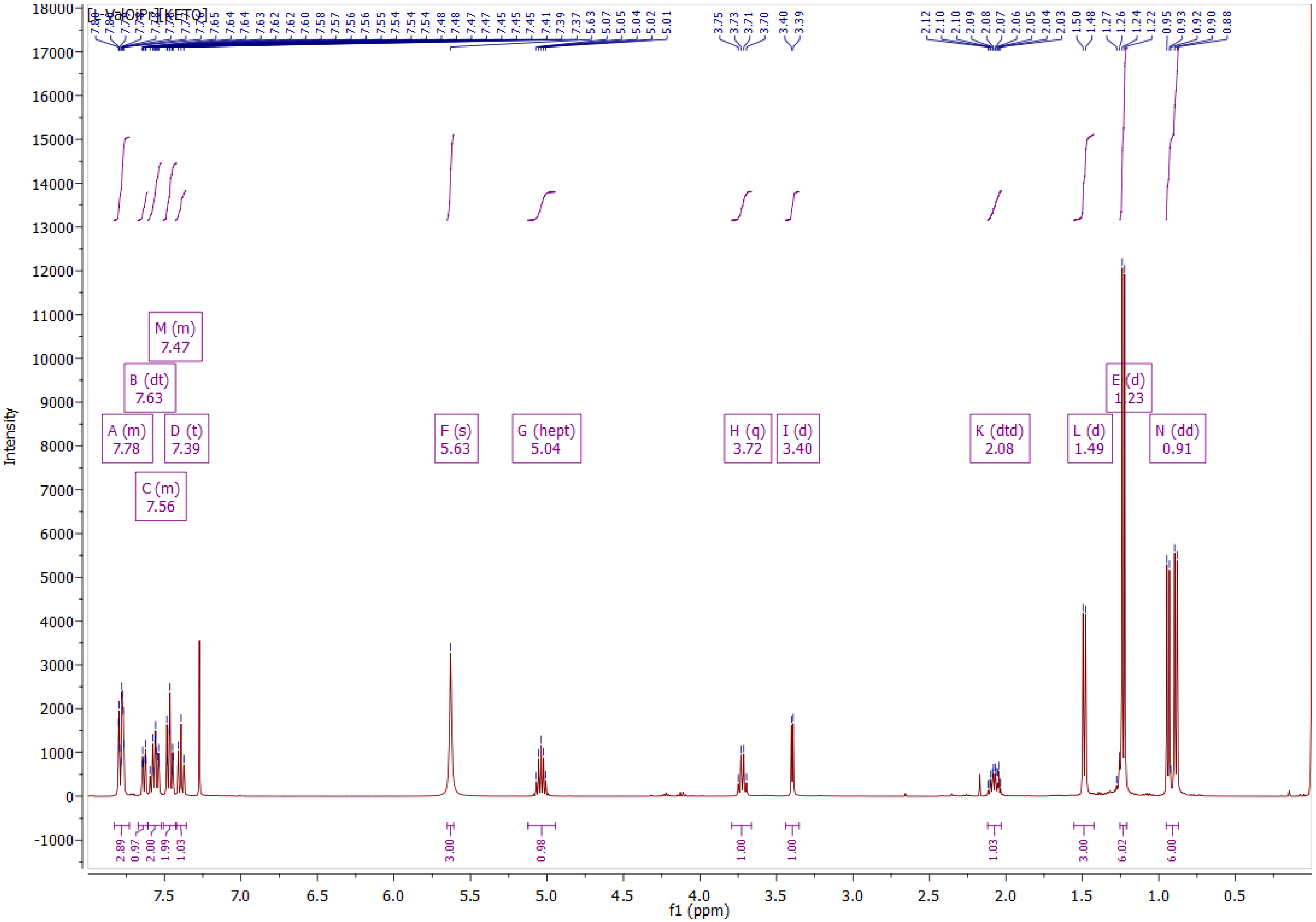

Fig. S7. ¹H NMR spectra of L-valine isopropyl ester ketoprofenate

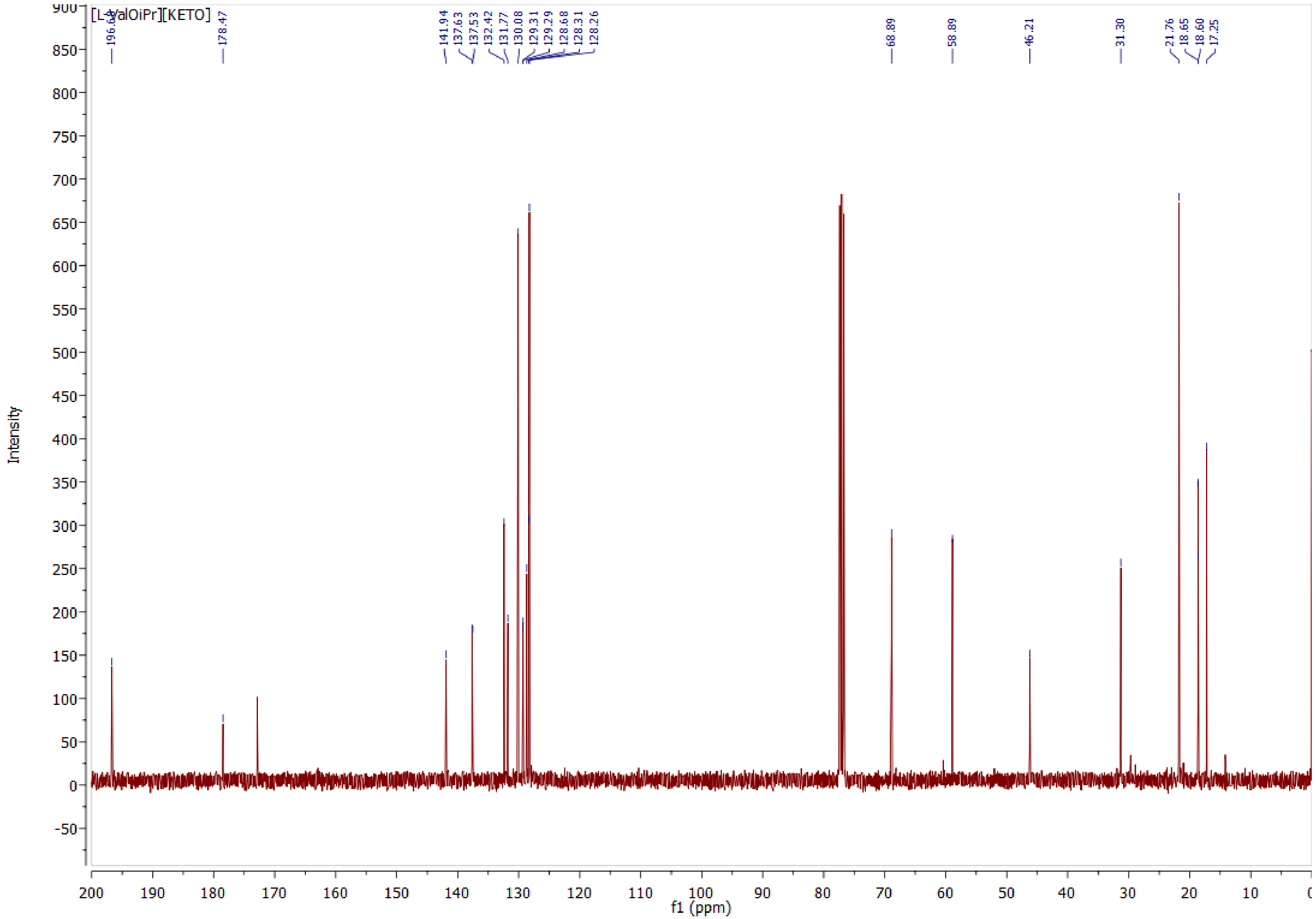

Fig. S8. ^{13}C NMR spectra of L-valine isopropyl ester ketoprofenate

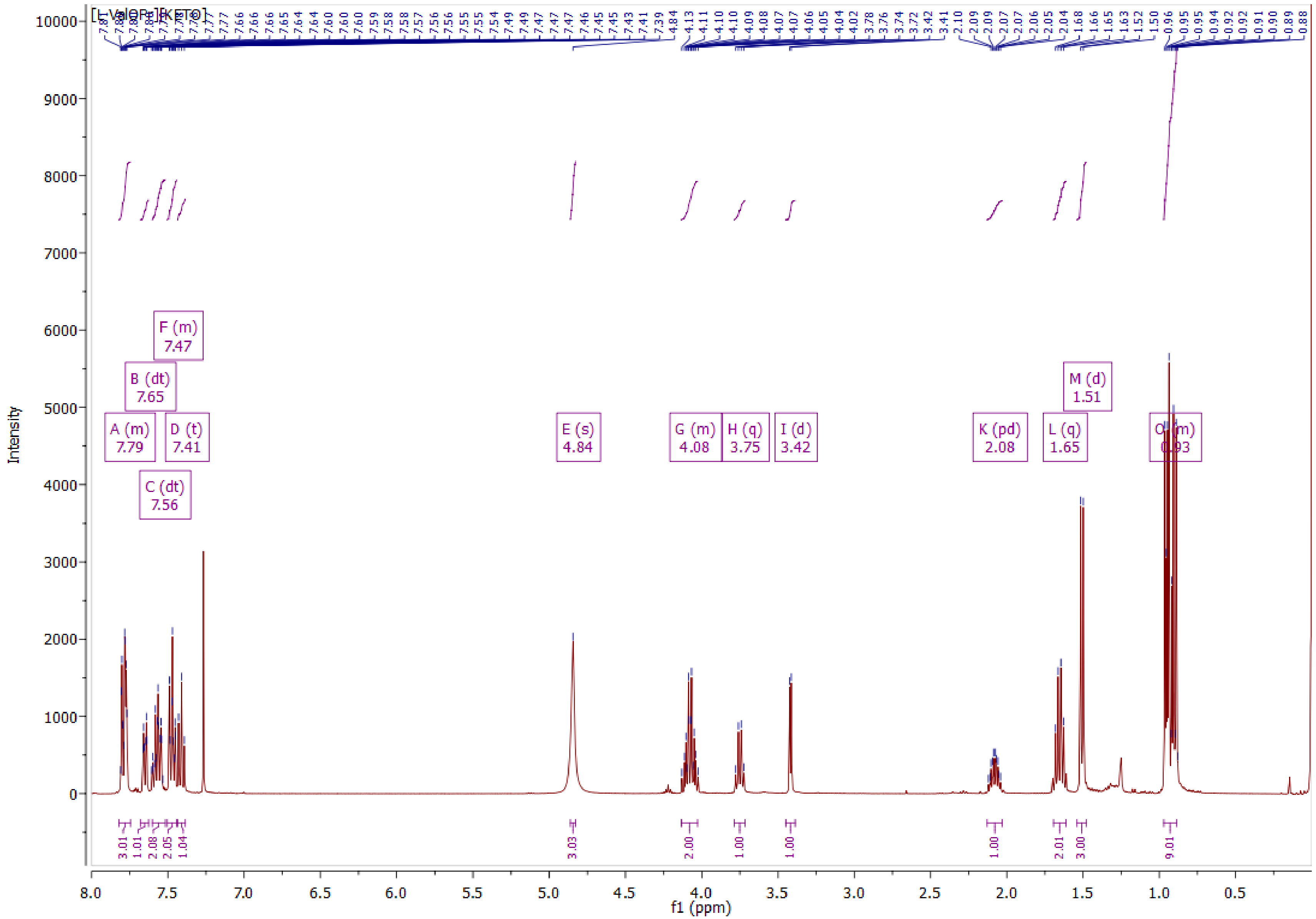

Fig. S9. 1H NMR spectra of L-valine propyl ester ketoprofenate

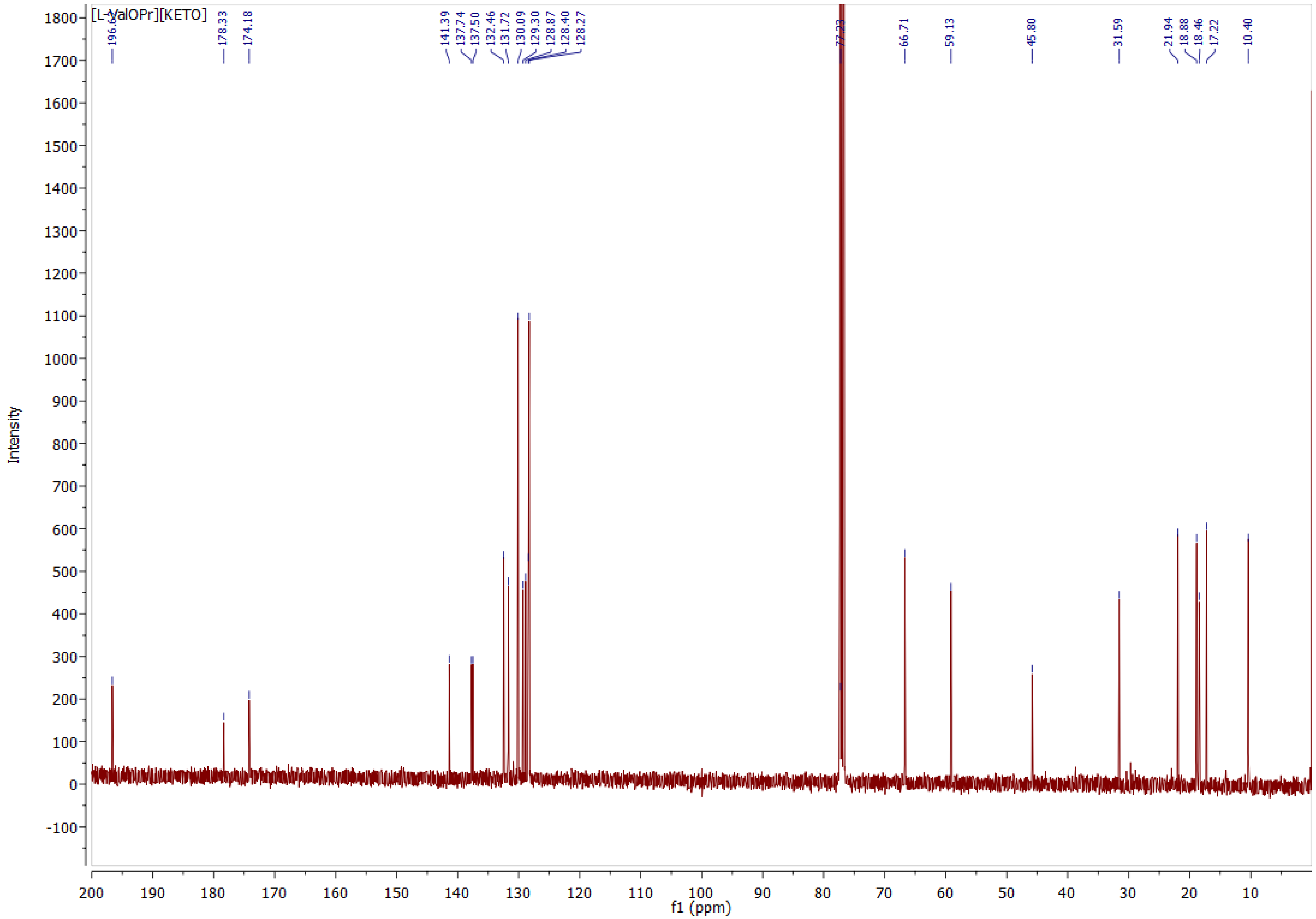

Fig. S10. ^{13}C NMR spectra of L-valine propyl ester ketoprofenate

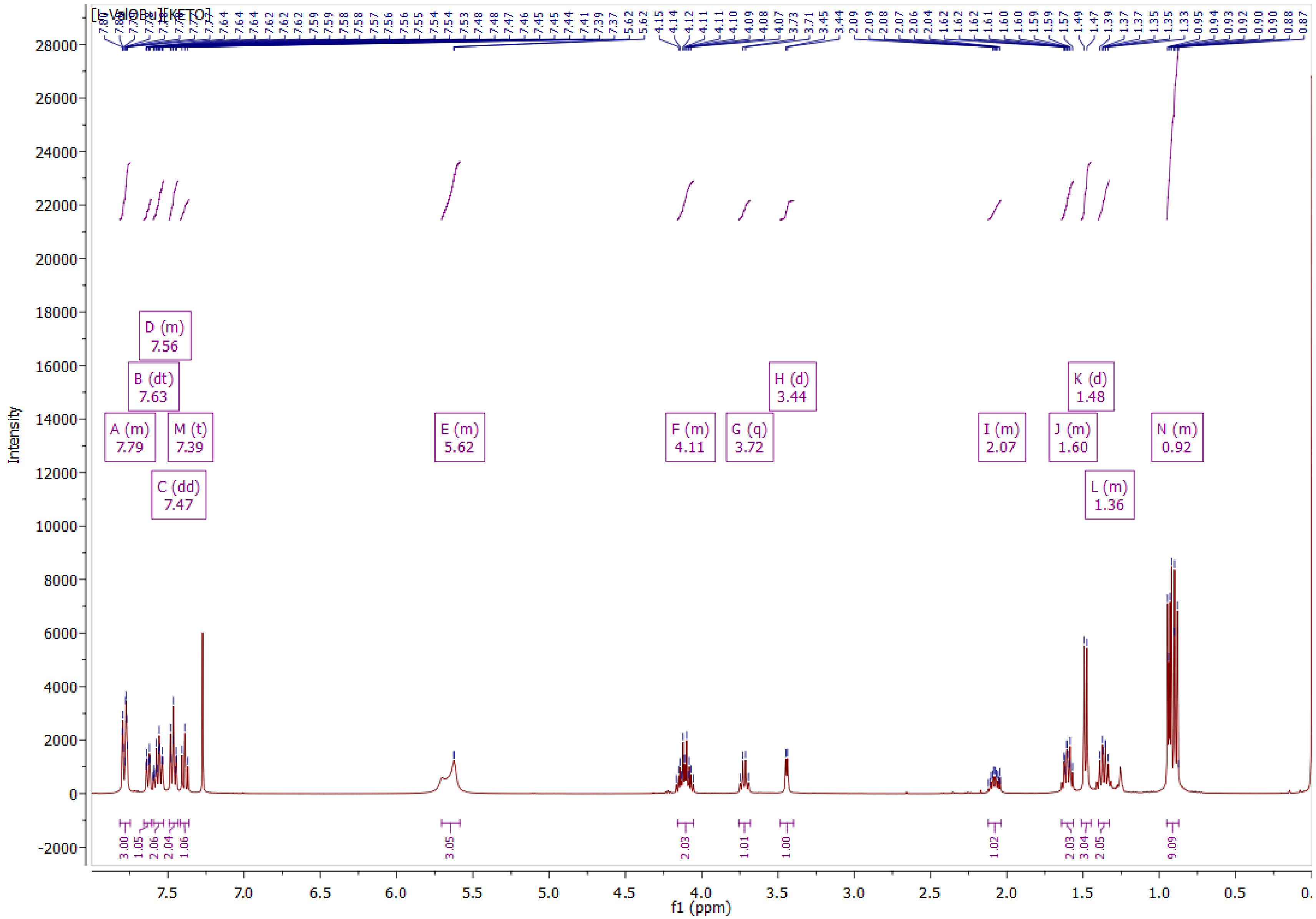

Fig. S11. ^{1}H NMR spectra of L-valine butyl ester ketoprofenate

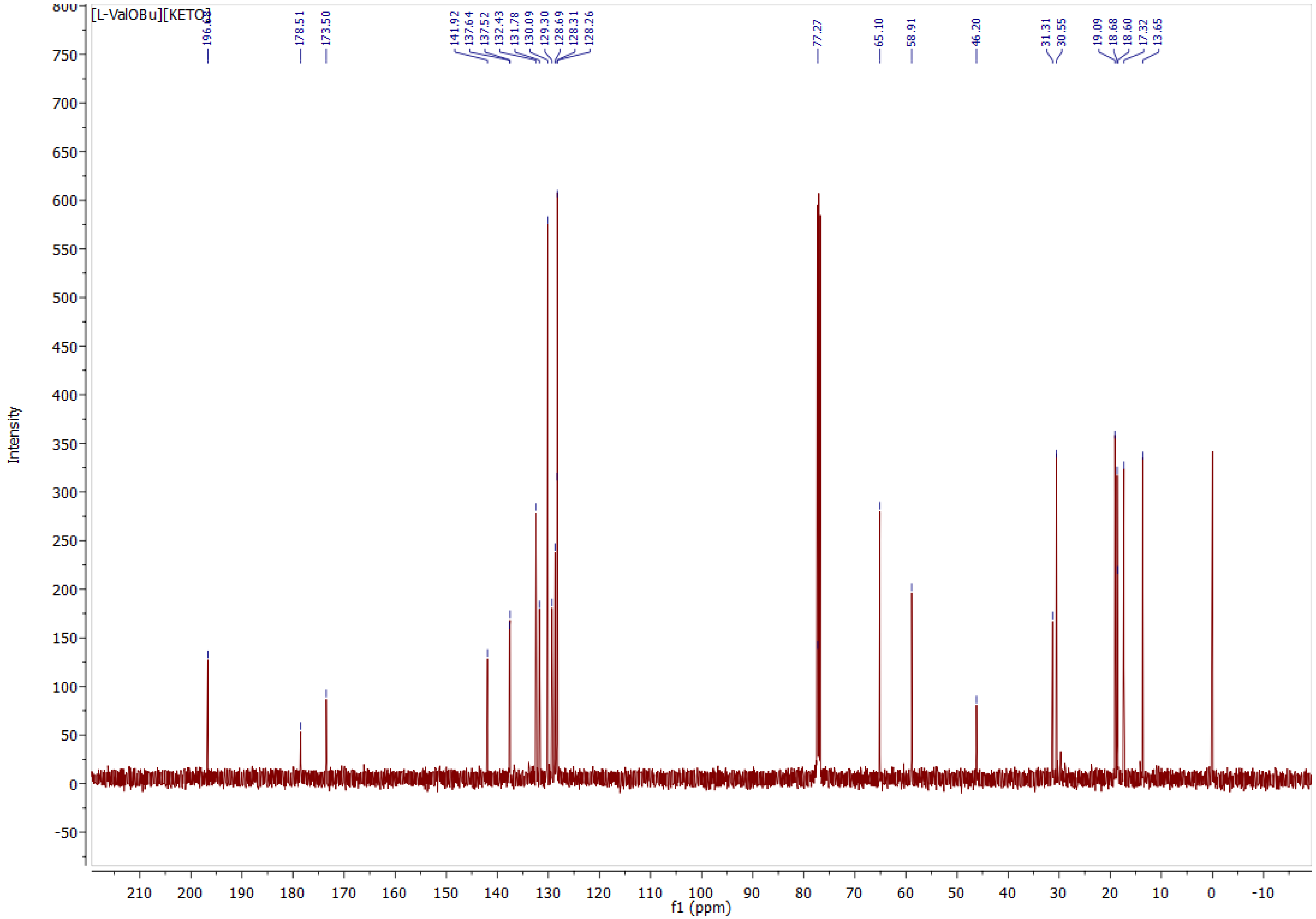

Fig. 12. ^{13}C NMR spectra of L-valine butyl ester ketoprofenate

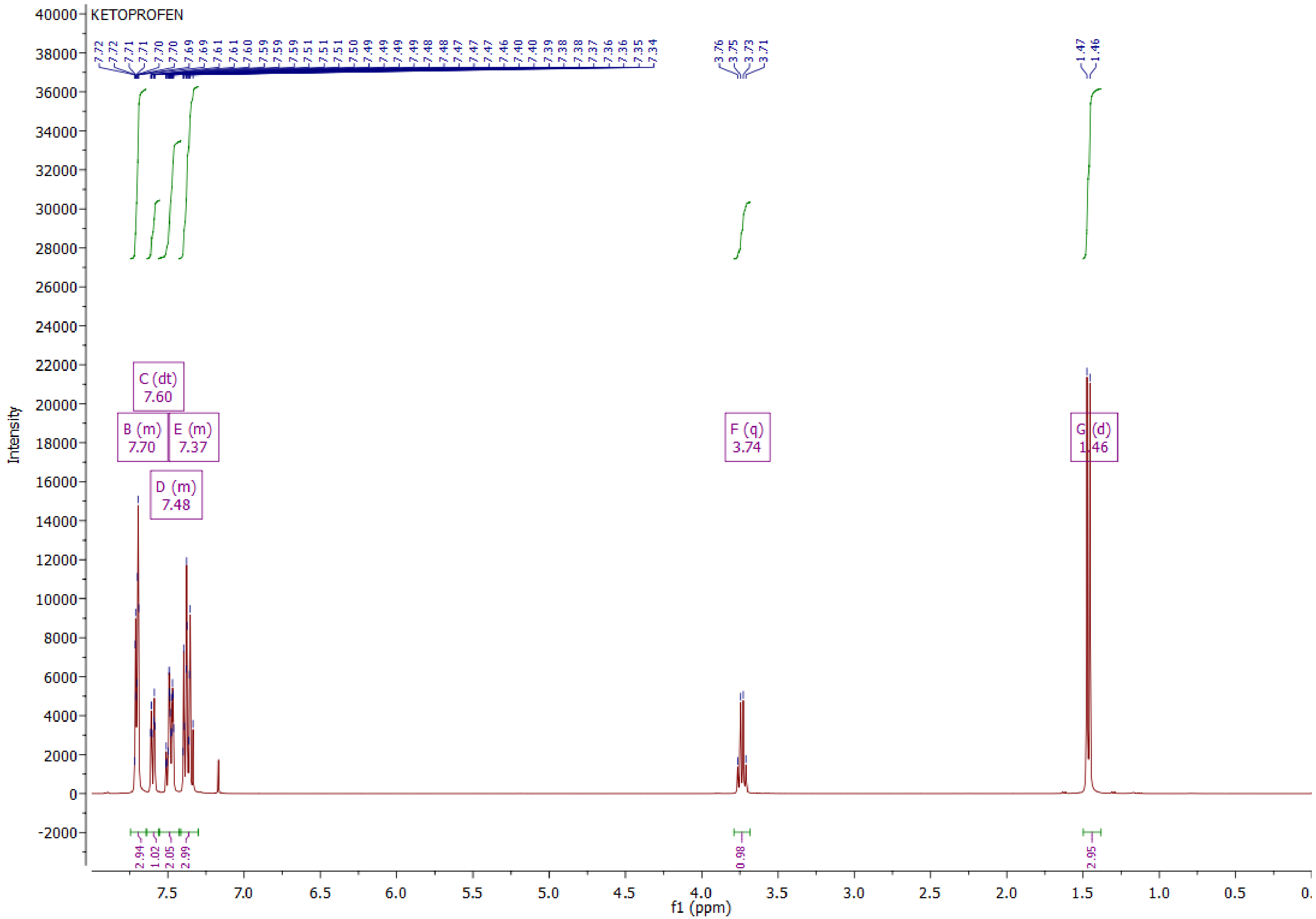

Fig.S13. ^{1}H NMR spectra of ketoprofen

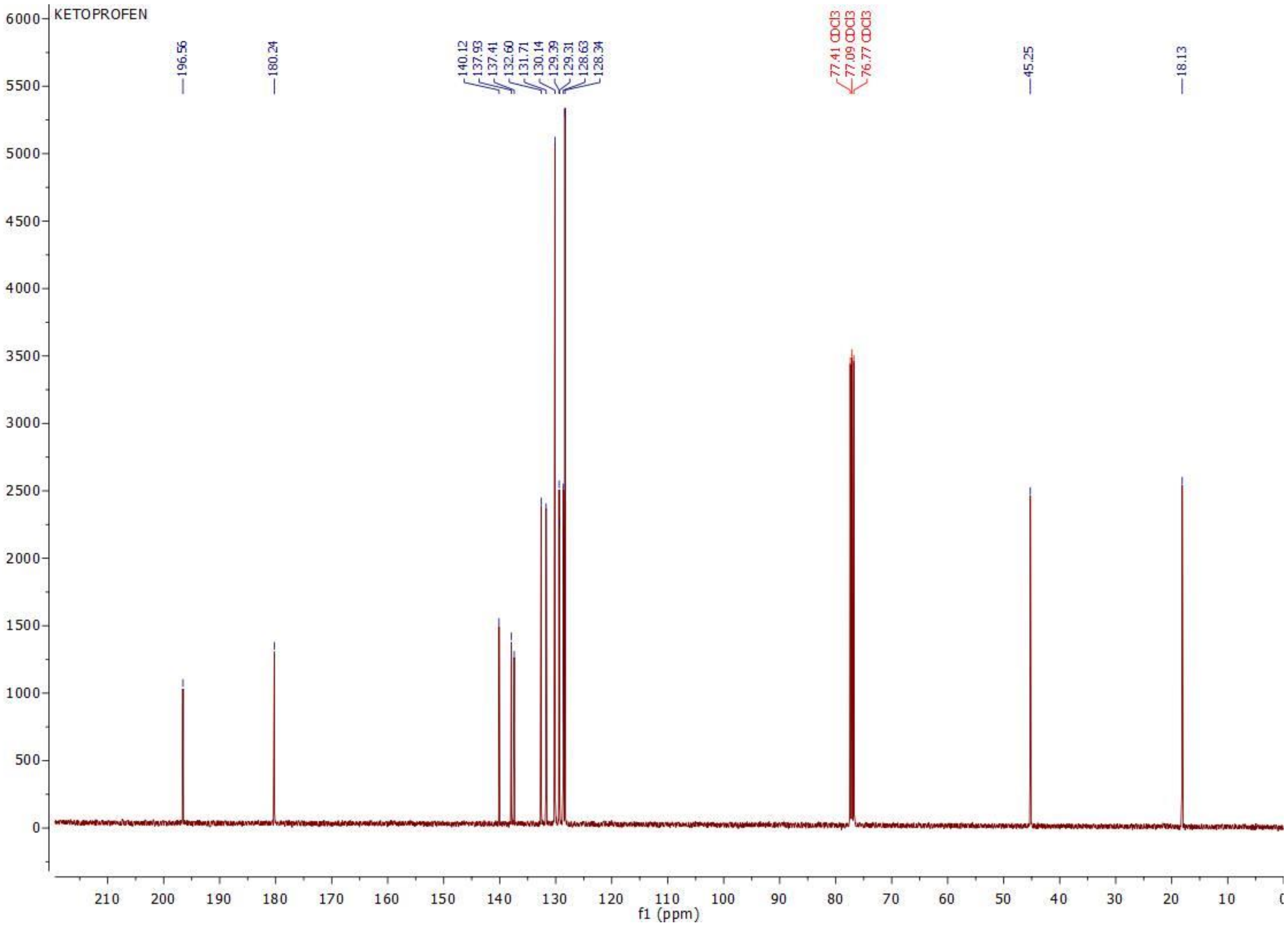

Fig. S14. ^{13}C NMR spectra of ketoprofen

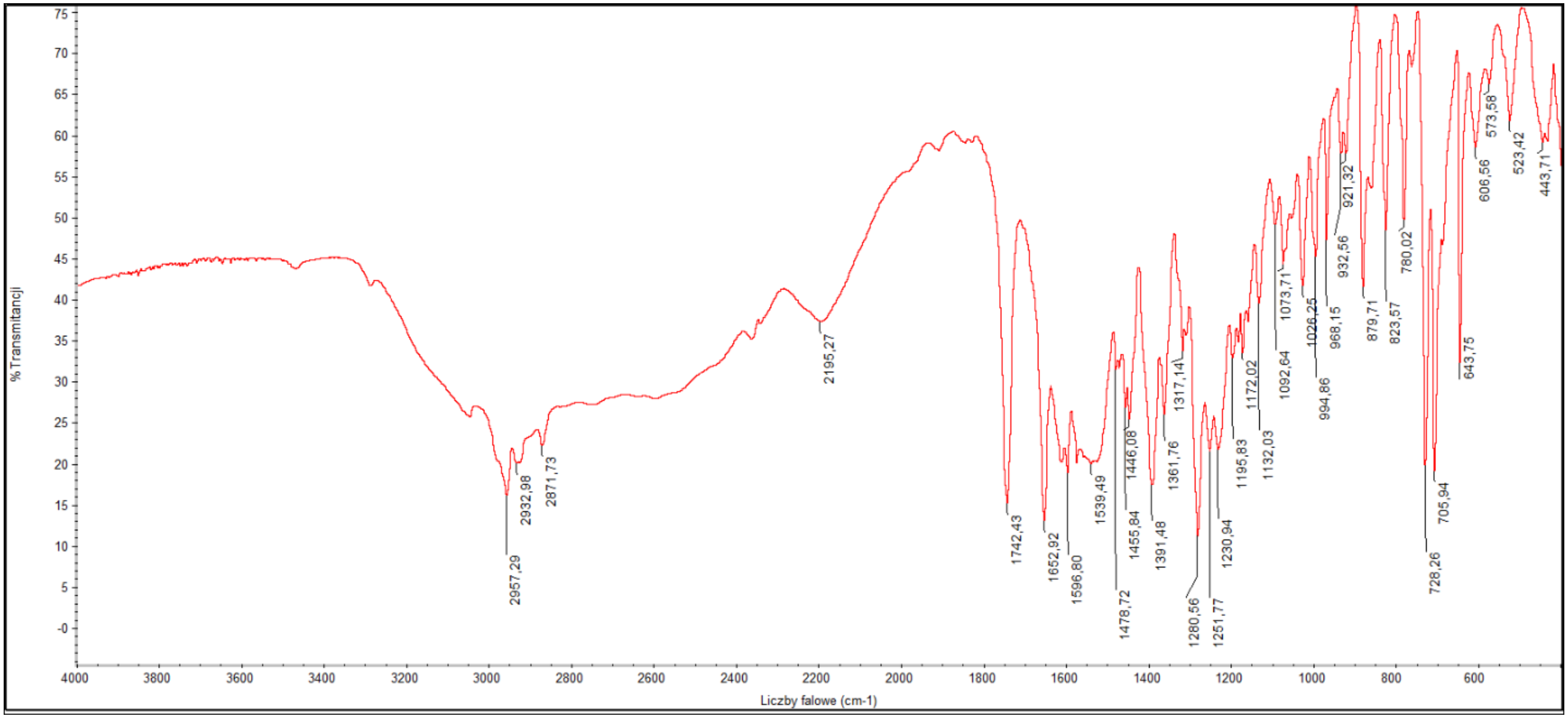

Fig. S15. FT-IR spectra of [L-LeuOEt][KETO]

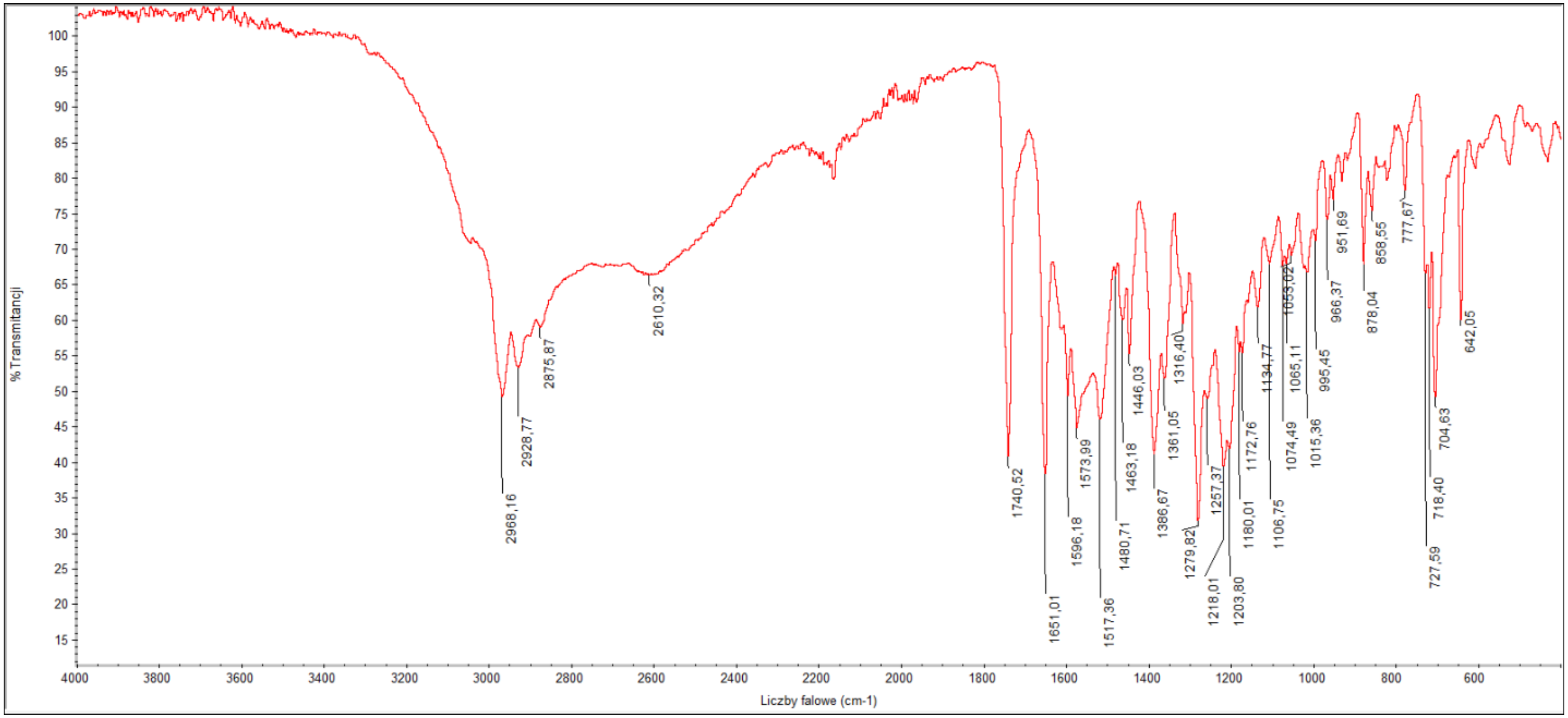

Fig. S16. FT-IR spectra of [L-ValOEt][KETO]

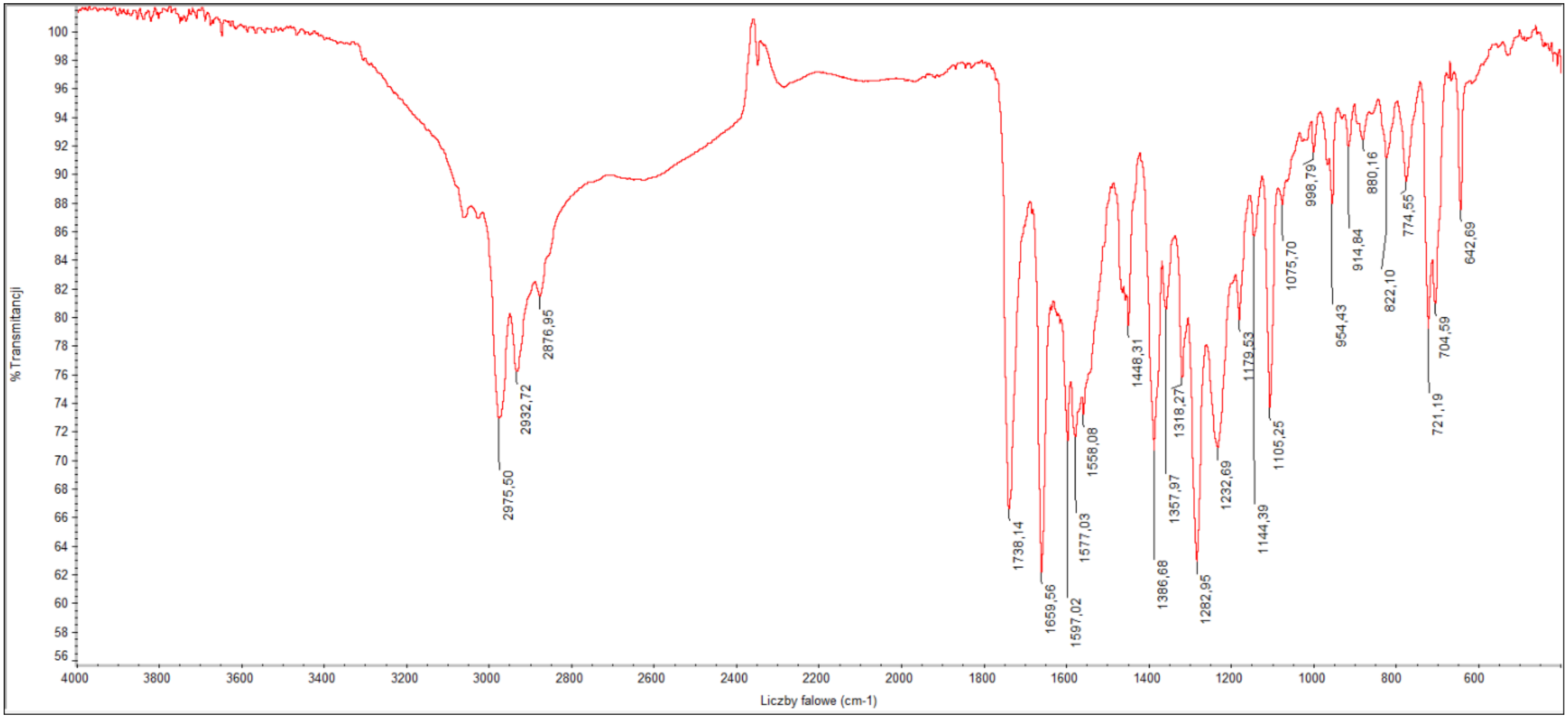

Fig. S17. FT-IR spectra of [L-ValOiPr][KETO]

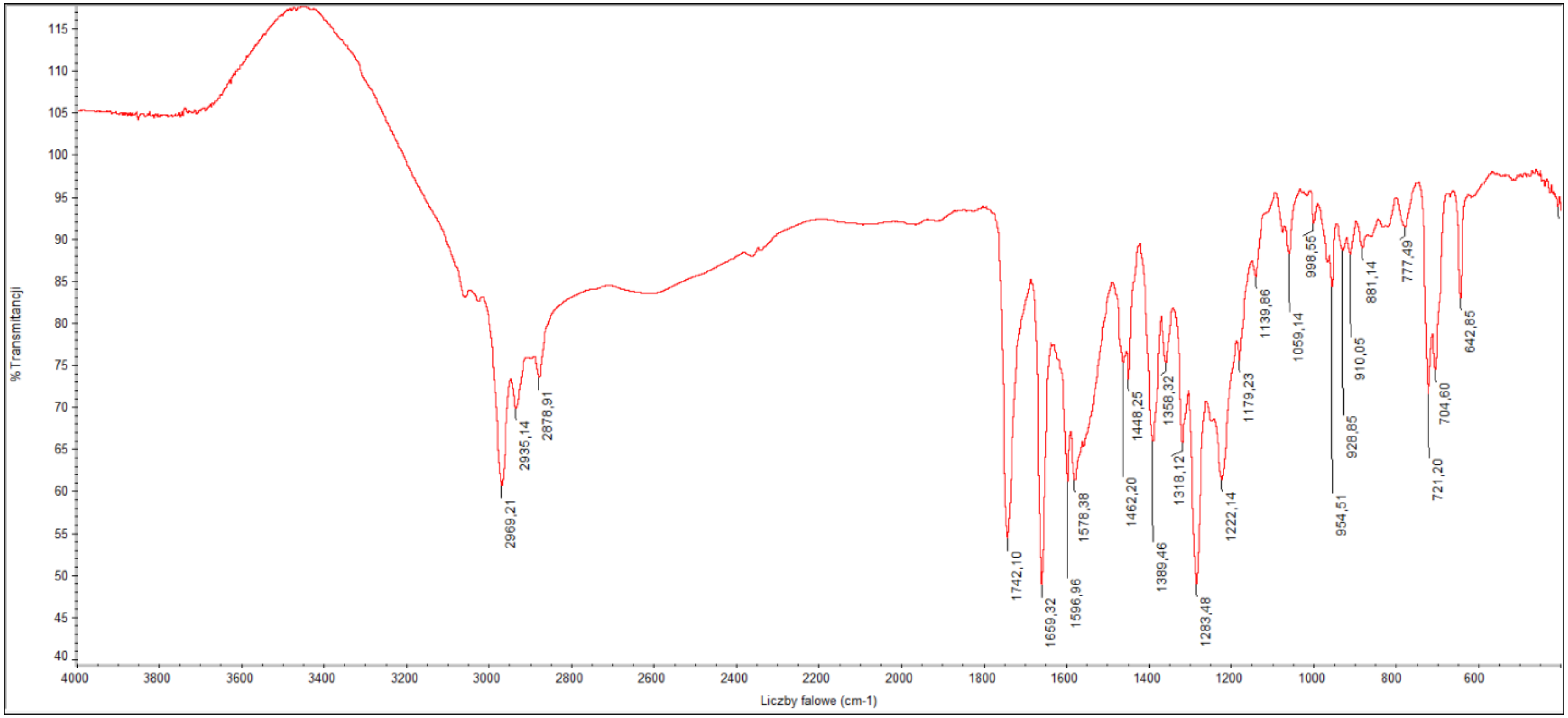

Fig. S18. FT-IR spectra of [L-ValOPr][KETO]

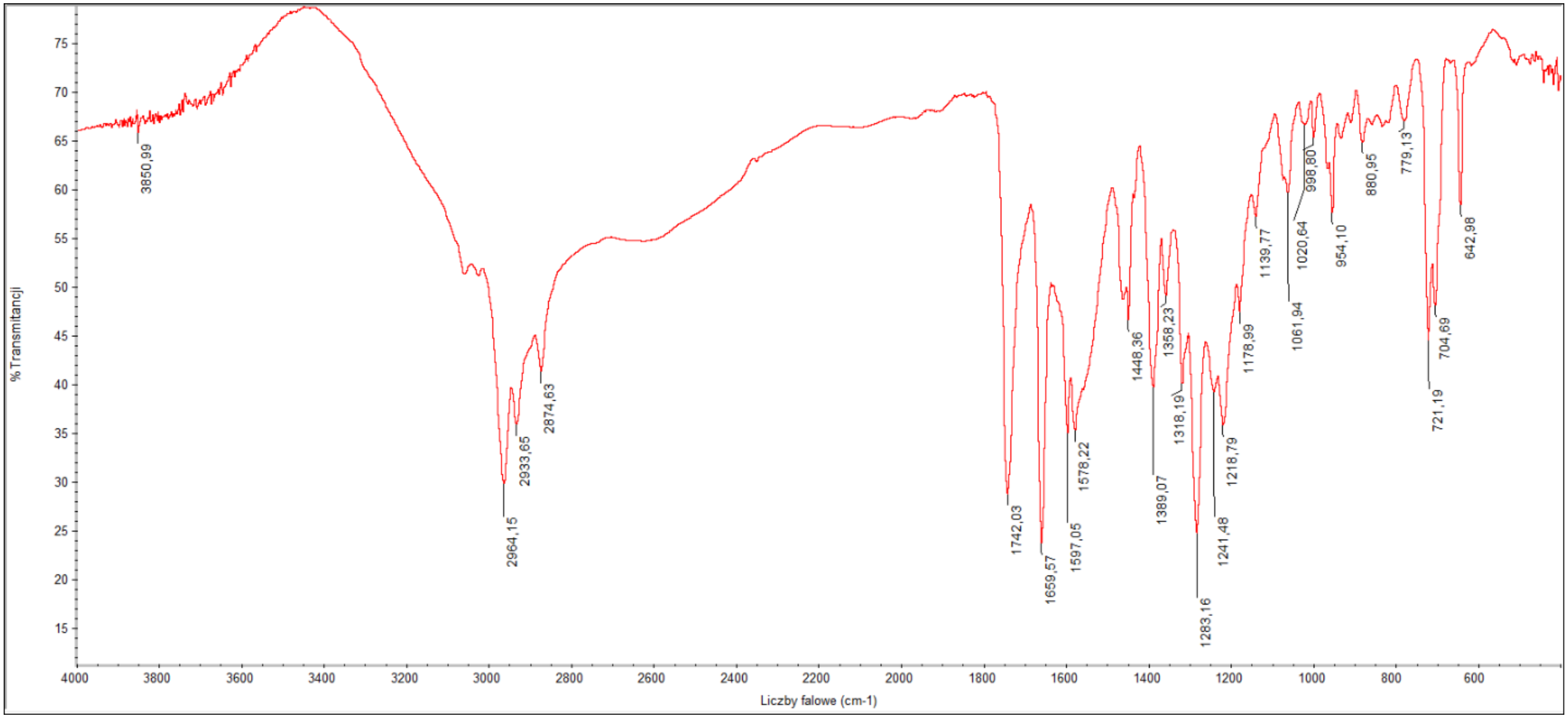

Fig. S19. FT-IR spectra of [L-ValOBu][KETO]

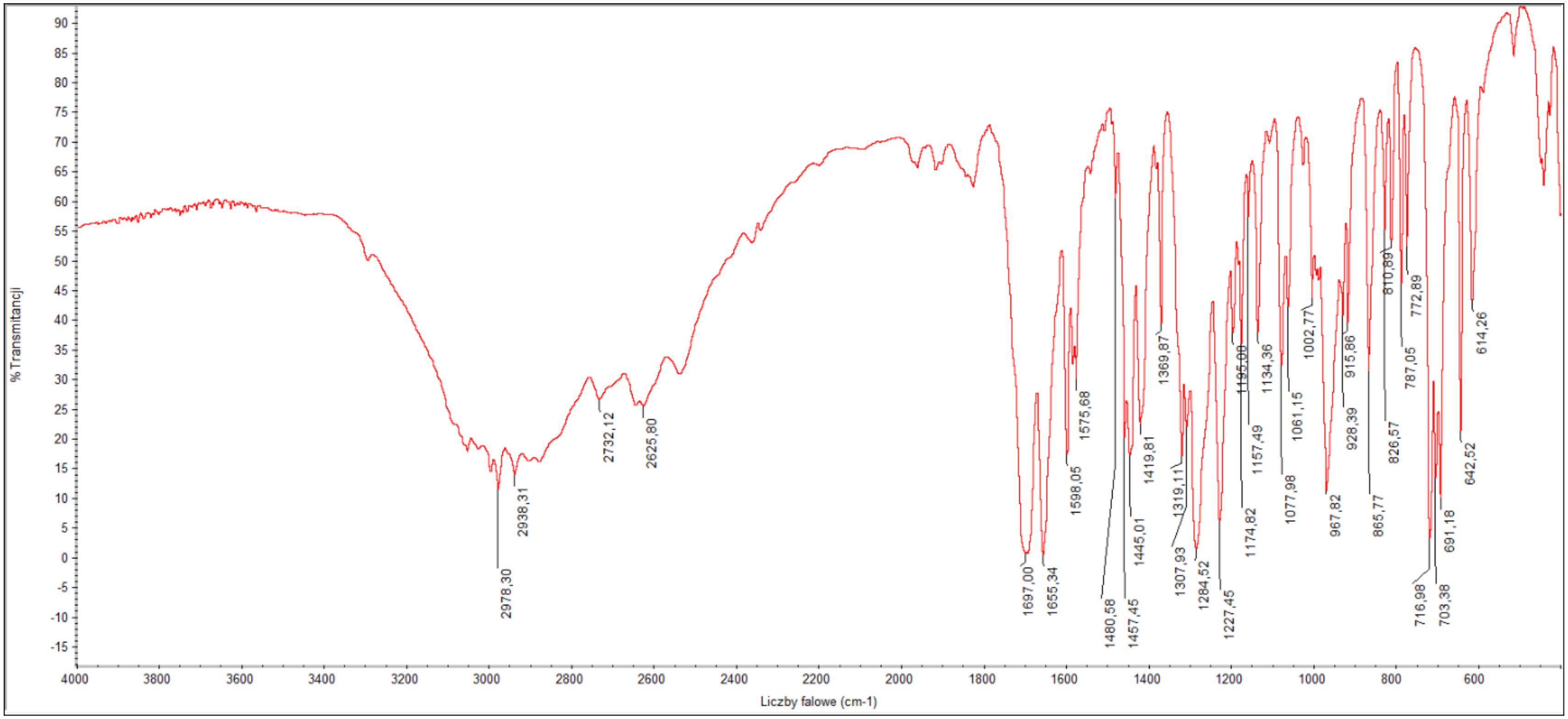

Fig. S20. FT-IR spectra of [KETO]

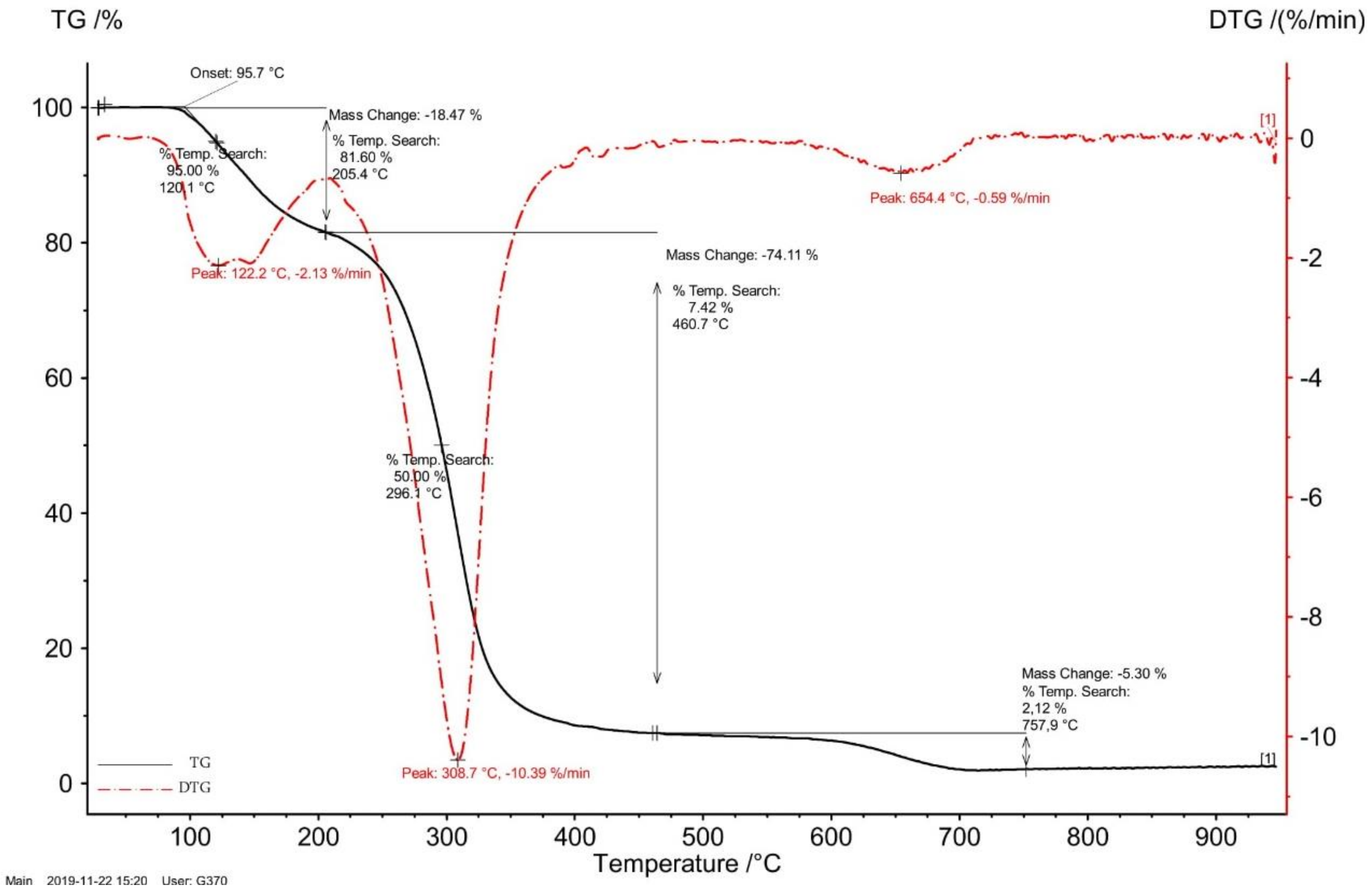

Fig. S21. The TG and DTG curves of [L-LeuOEt][KETO]

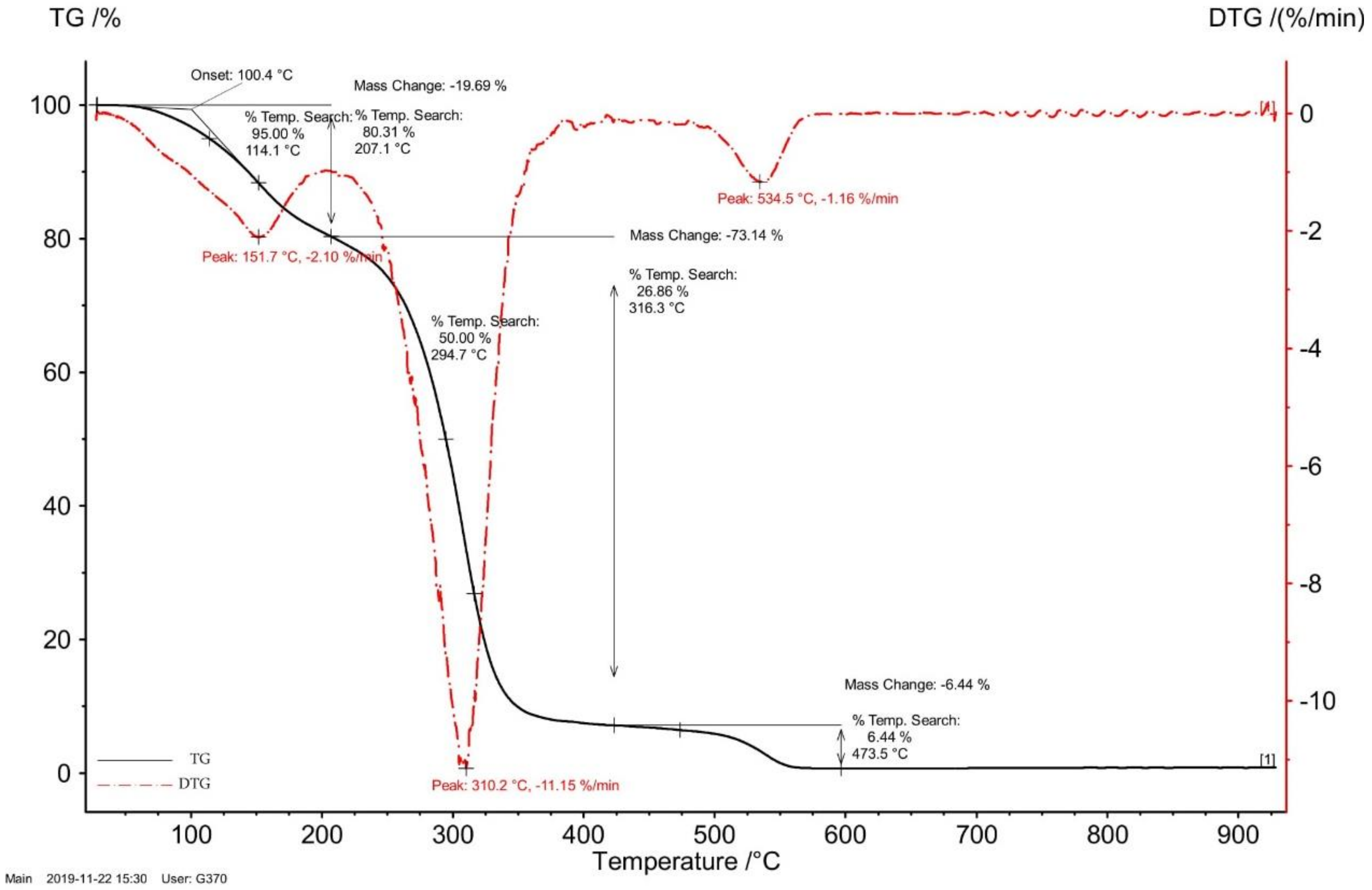

Fig. S22. The TG and DTG curves of [L-ValOEt][KETO]

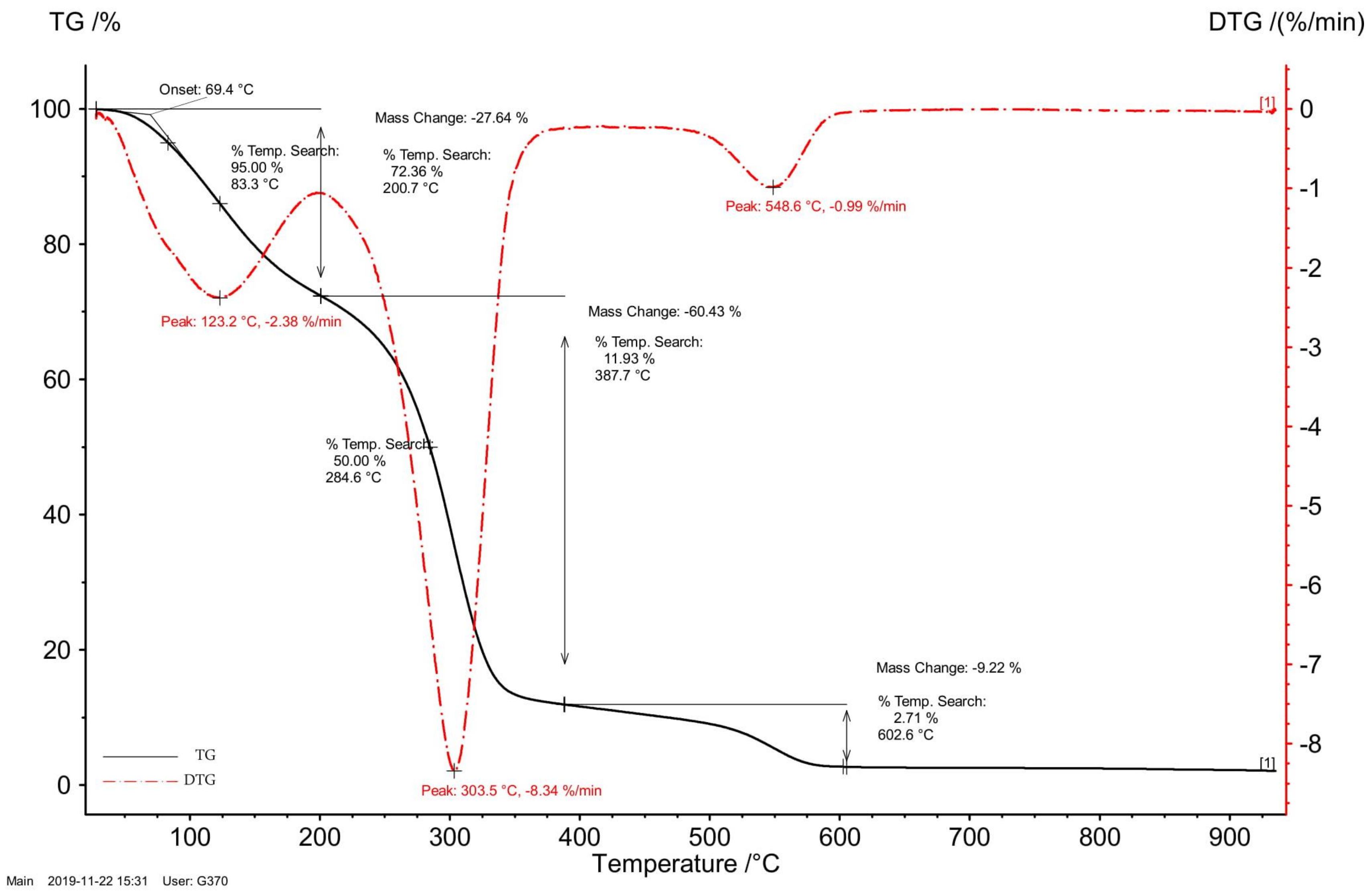

Fig. S23. The TG and DTG curves of [L-ValOiPr][KETO]

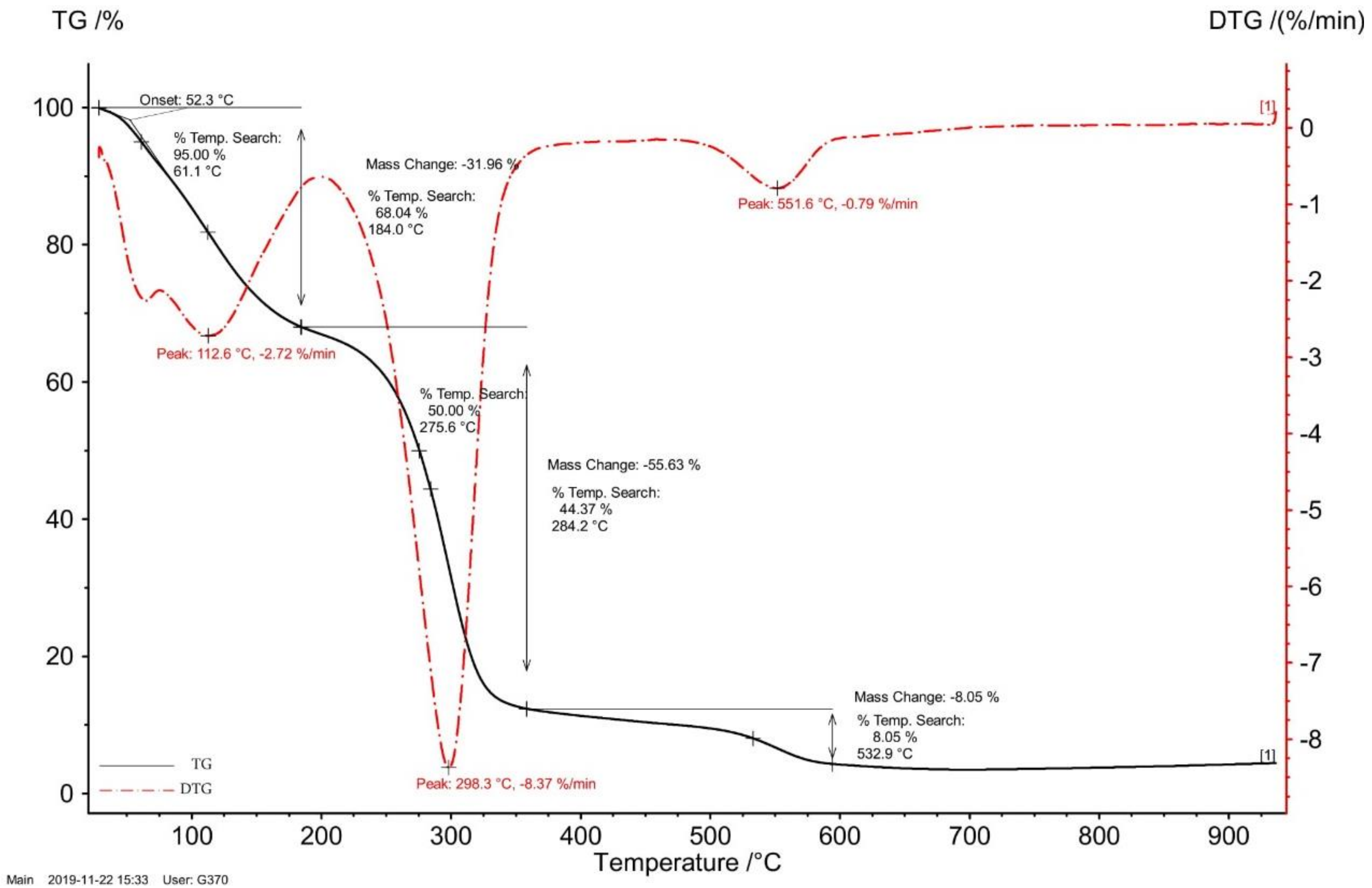

Fig. S24. The TG and DTG curves of [L-ValOPr][KETO]

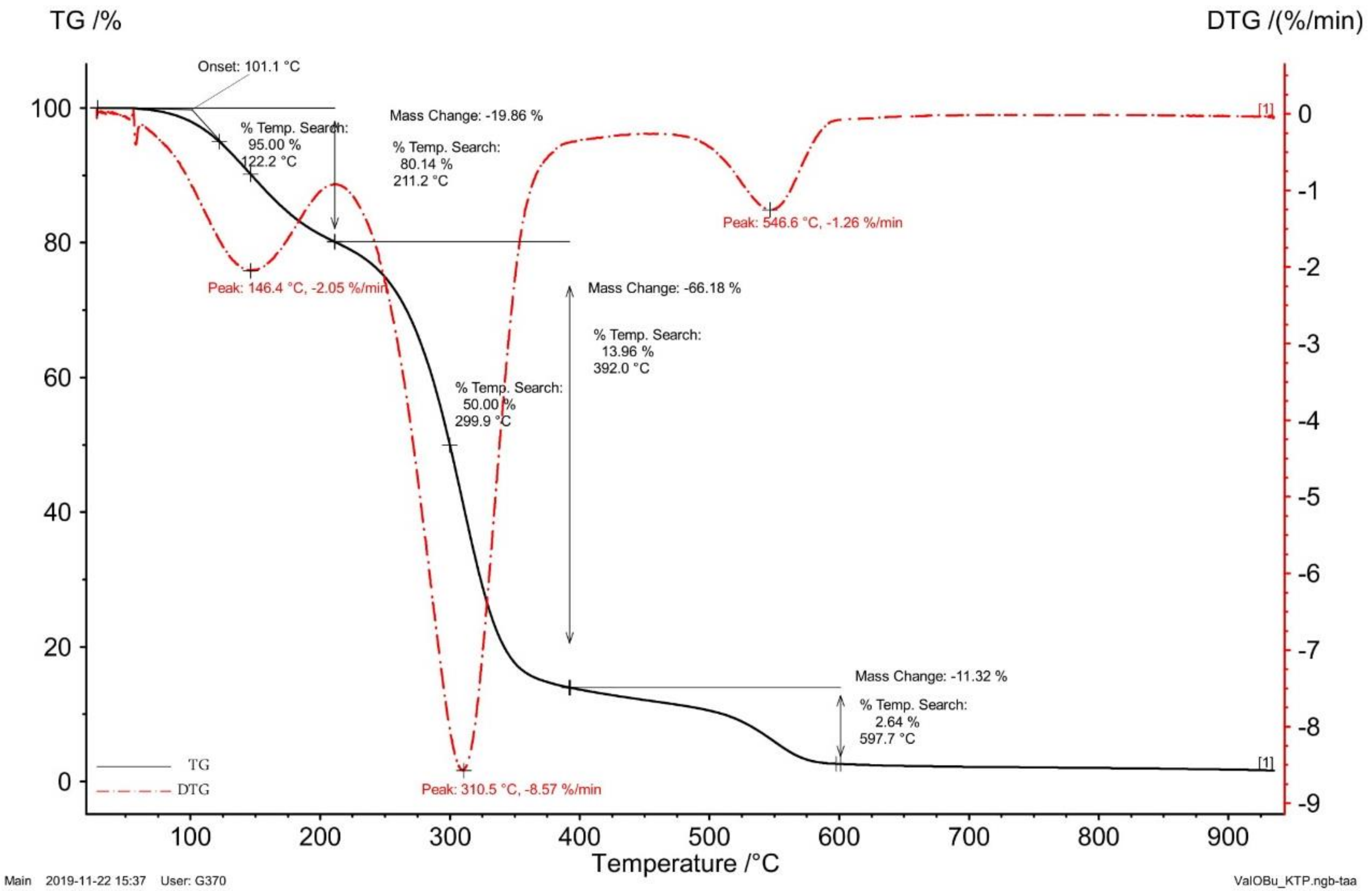

Fig. S25. The TG and DTG and curves of [L-ValOBu][KETO]

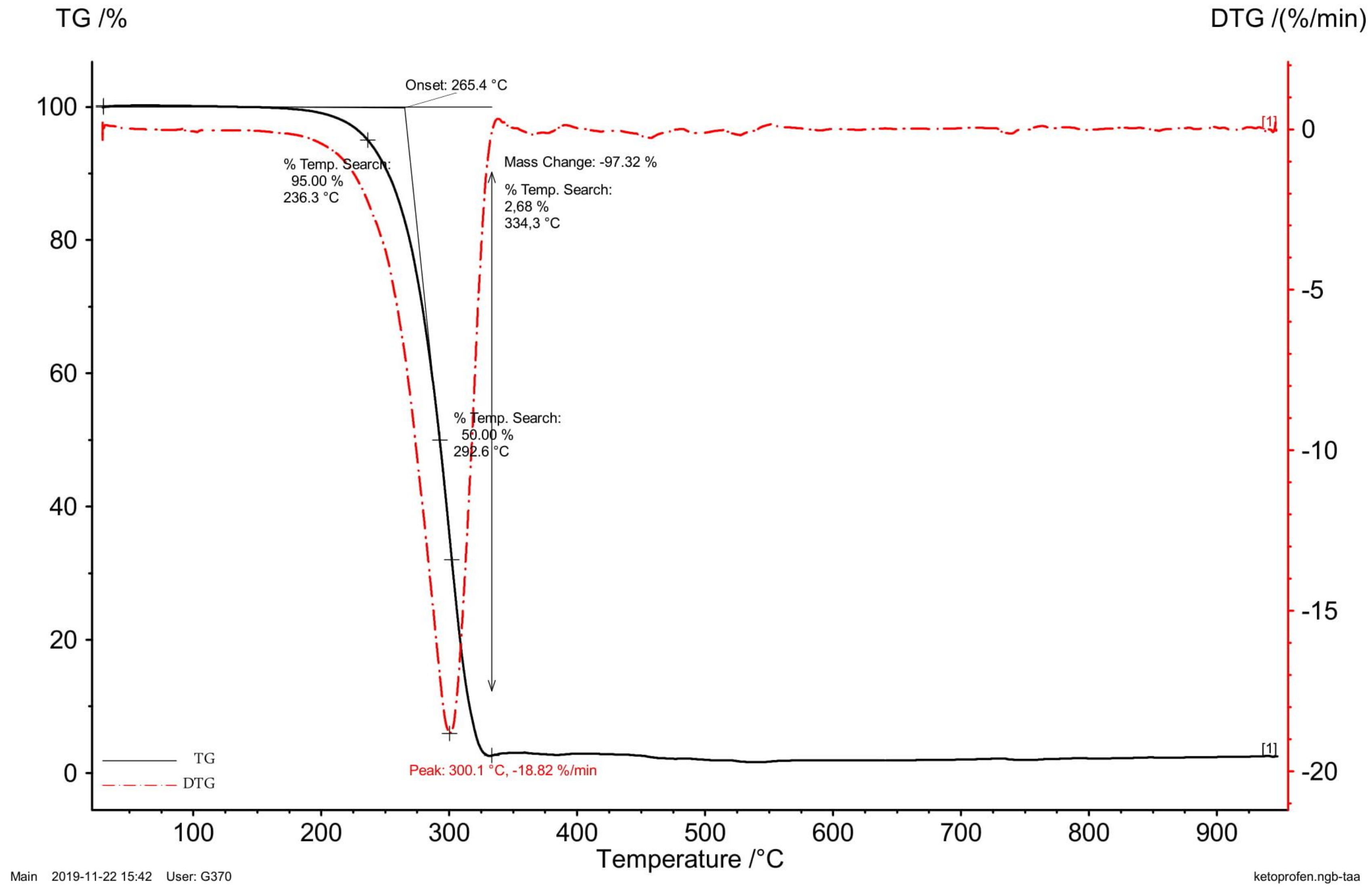

Fig. S26. The TG and DTG curves of [KETO]

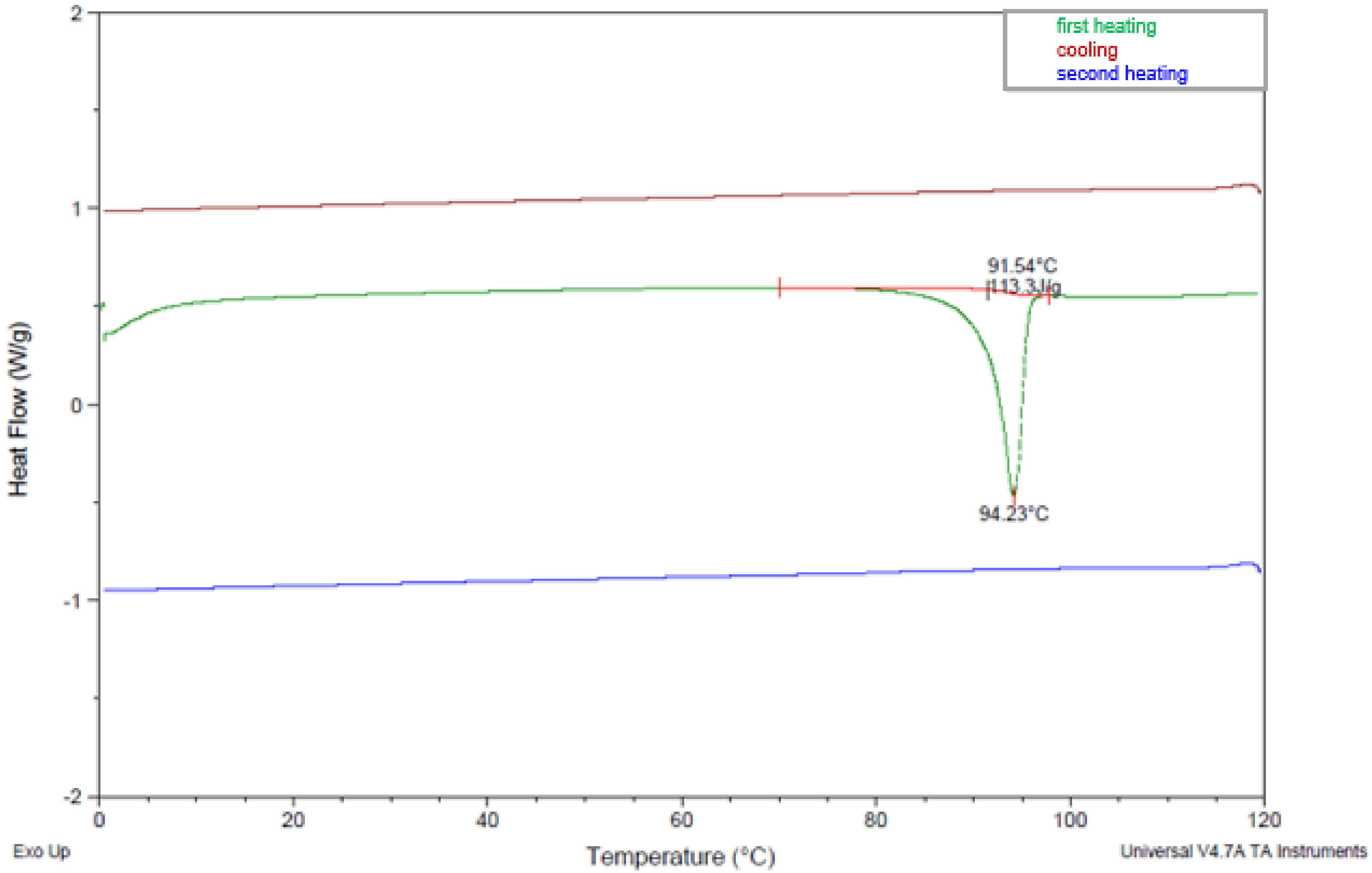

Figure S27. The DSC curves of [L-LeuOEt][KETO]

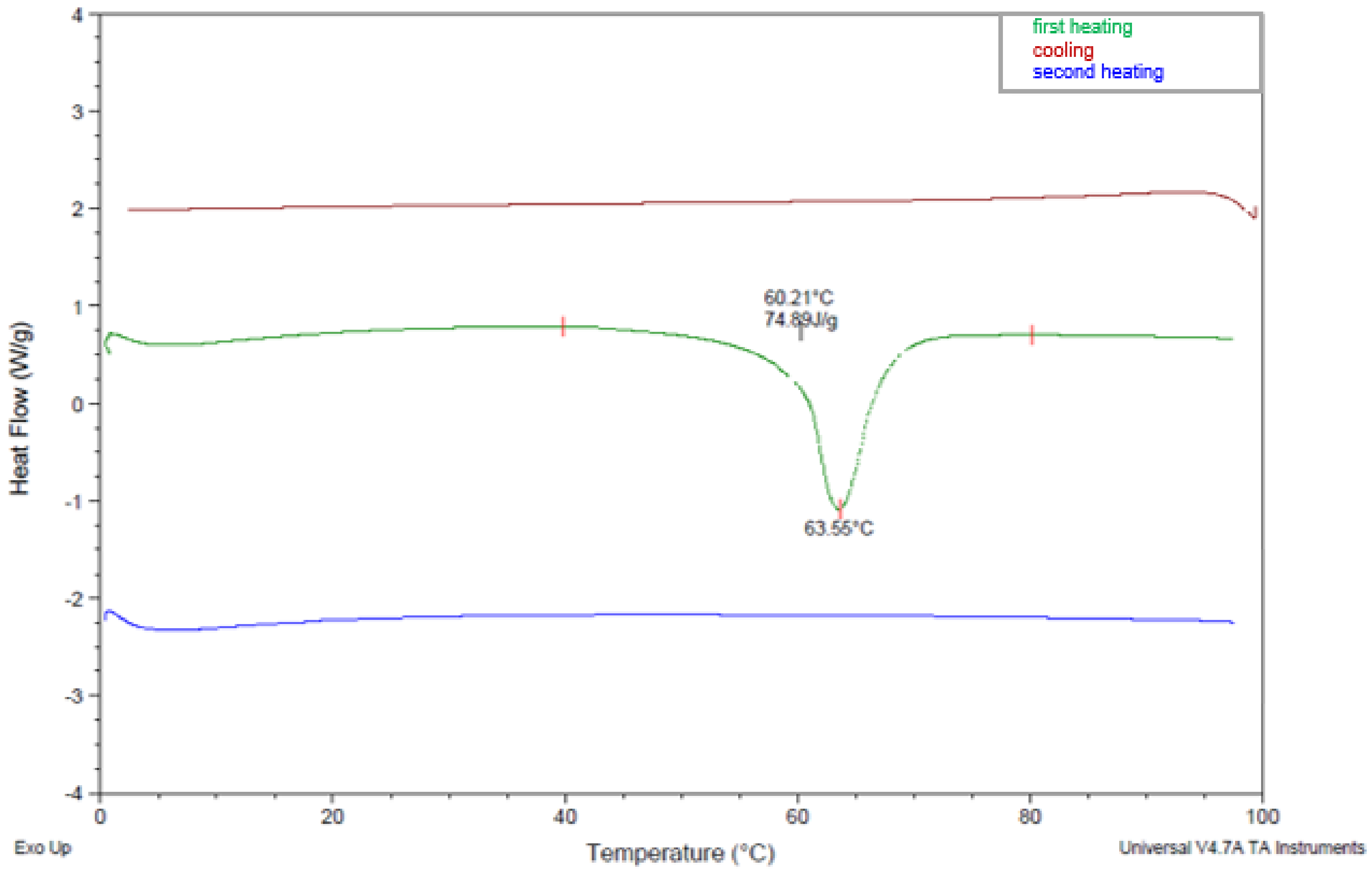

Figure S28. The DSC curves of [L-ValOEt][KETO]

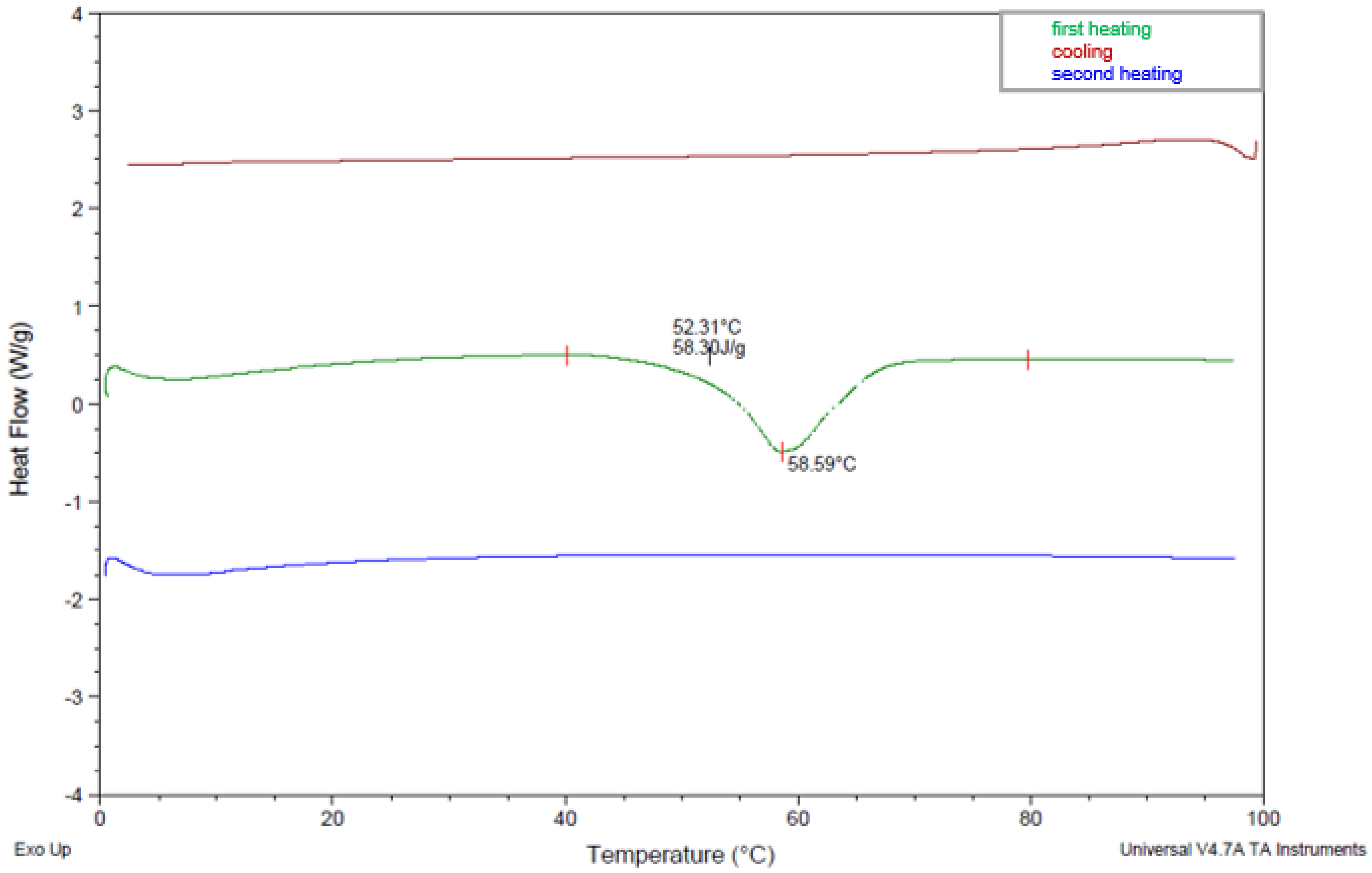

Figure S29. The DSC curves of [L-ValOiPr][KETO]

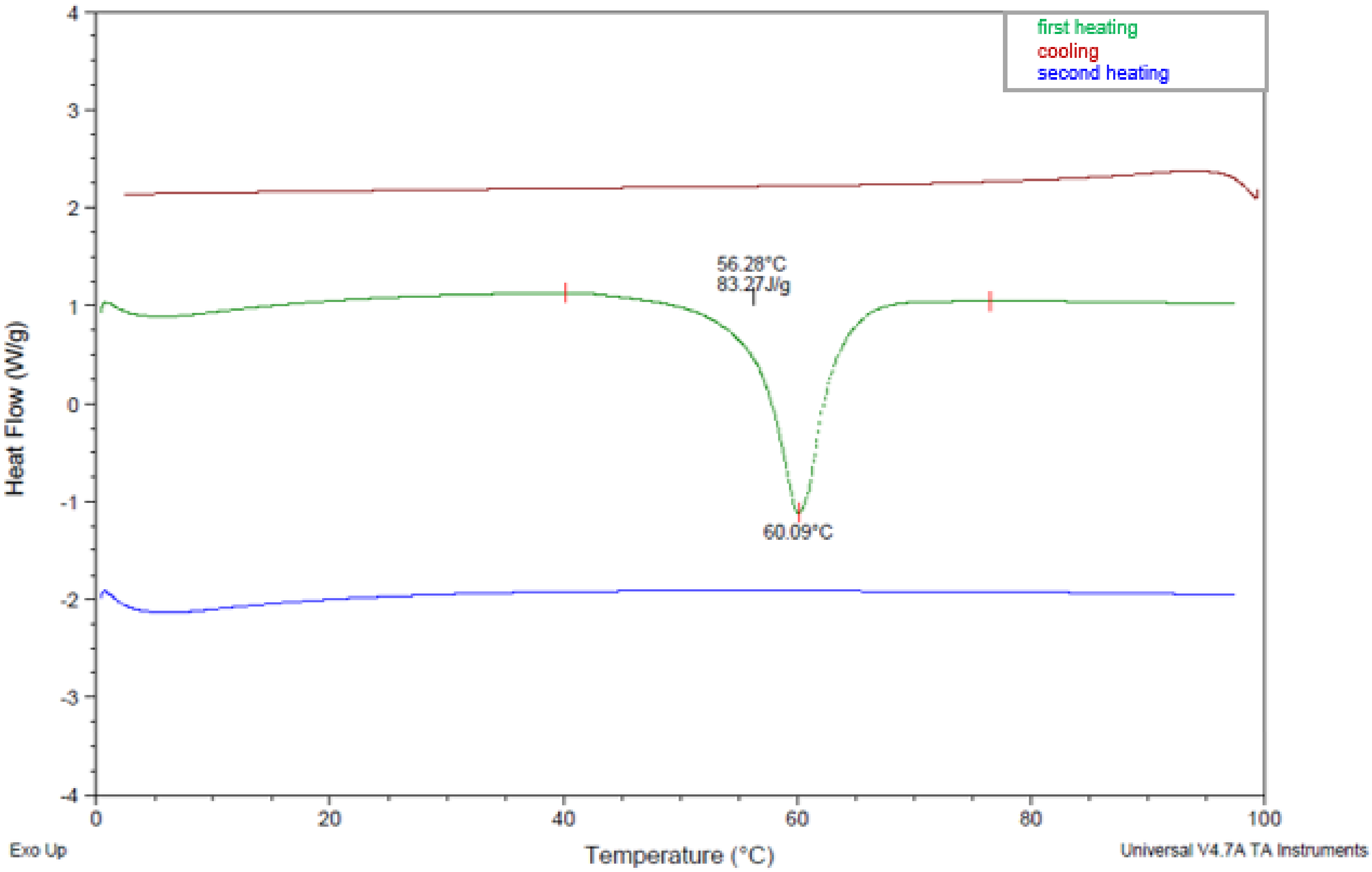

Figure S30. The DSC curves of [L-ValOPr][KETO]

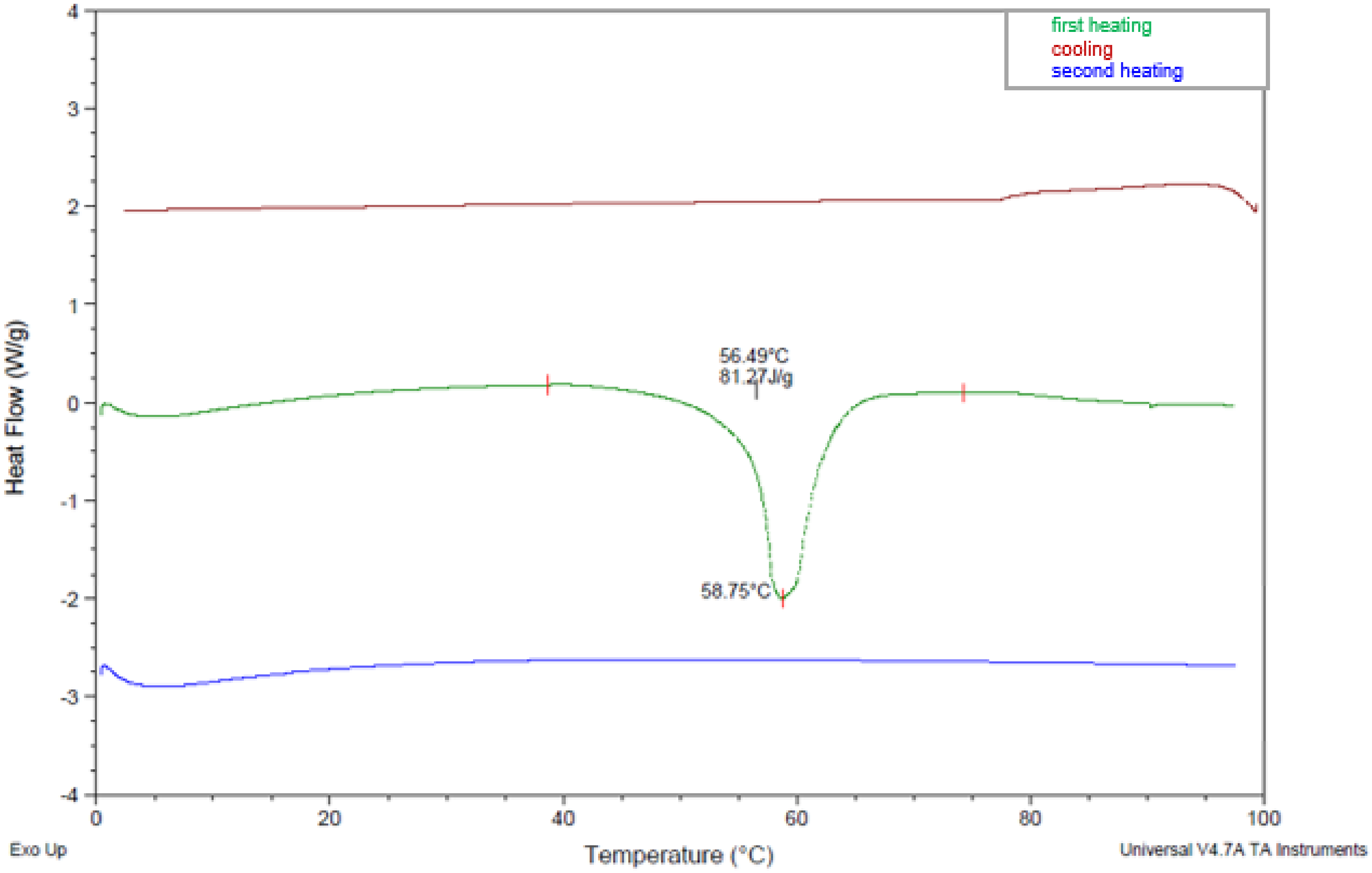

Figure S31. The DSC curves of [L-ValOBu][KETO]

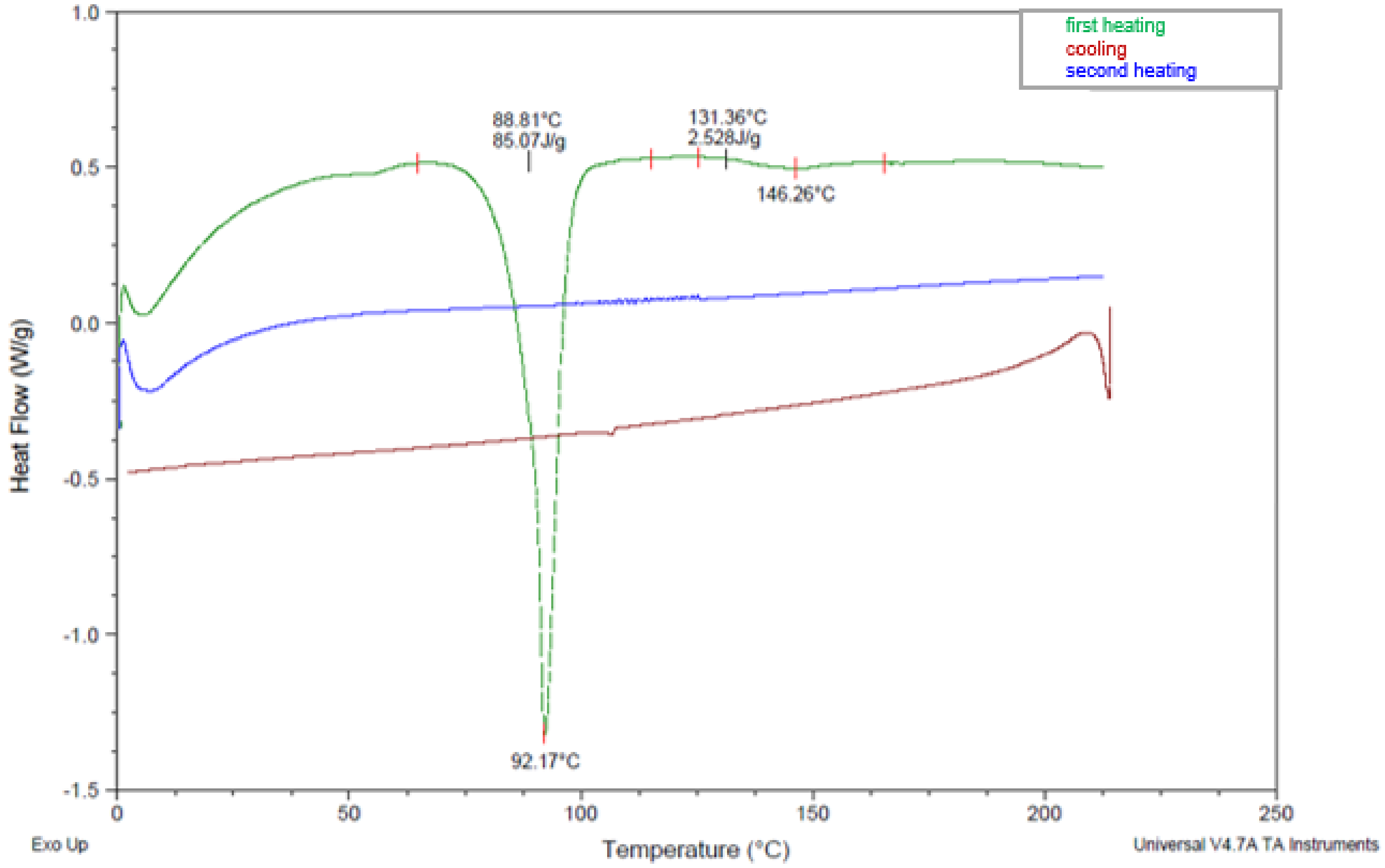

Figure S32. The DSC curves of [KETO]

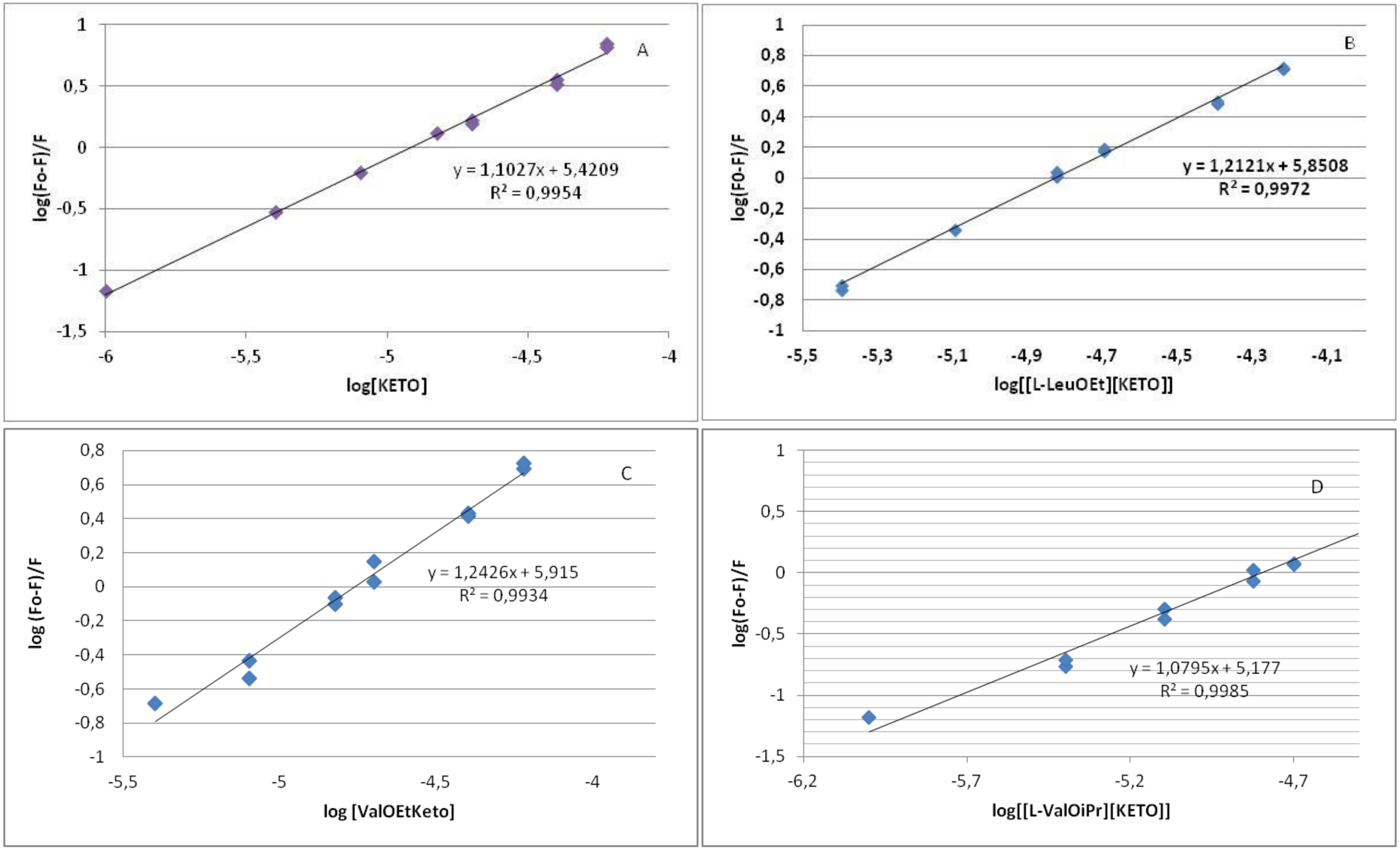
A
log(Fo-F)/F
log[KETO]
y = 1,1027x + 5,4209
R² = 0,9954
B
log(F0-F)/F
log[[L-LeuOEt][KETO]]
y = 1,2121x + 5,8508
R² = 0,9972
C
log (Fo-F)/F
log [ValOEtKeto]
y = 1,2426x + 5,915
R² = 0,9934
D
log(Fo-F)/F
log[[L-ValOiPr][KETO]]
y = 1,0795x + 5,177
R² = 0,9985

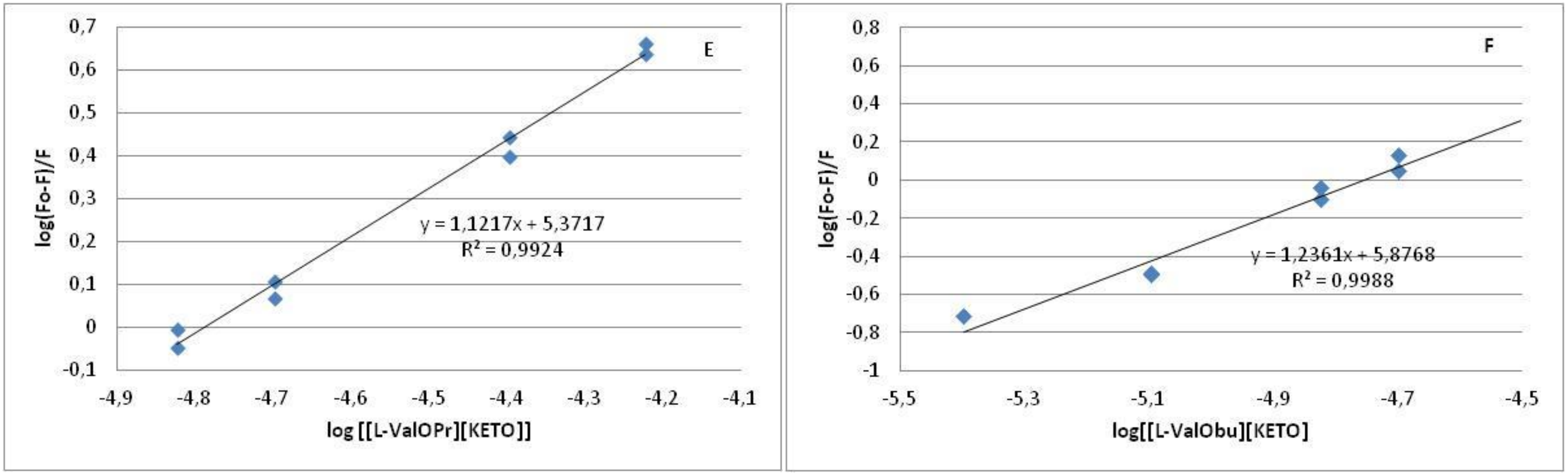

Fig. S33. The plot of log (Fo-F)/F vs log. [Q] at 25ºC, where Q is ketoprofen (A); [L-LeuOEt][KETO] (B); [L-ValOEt][KETO] (C); [L-ValOiPr][KETO] (D) [L-ValOPr][KETO] (E) and [L-ValOBu][KETO] (F).

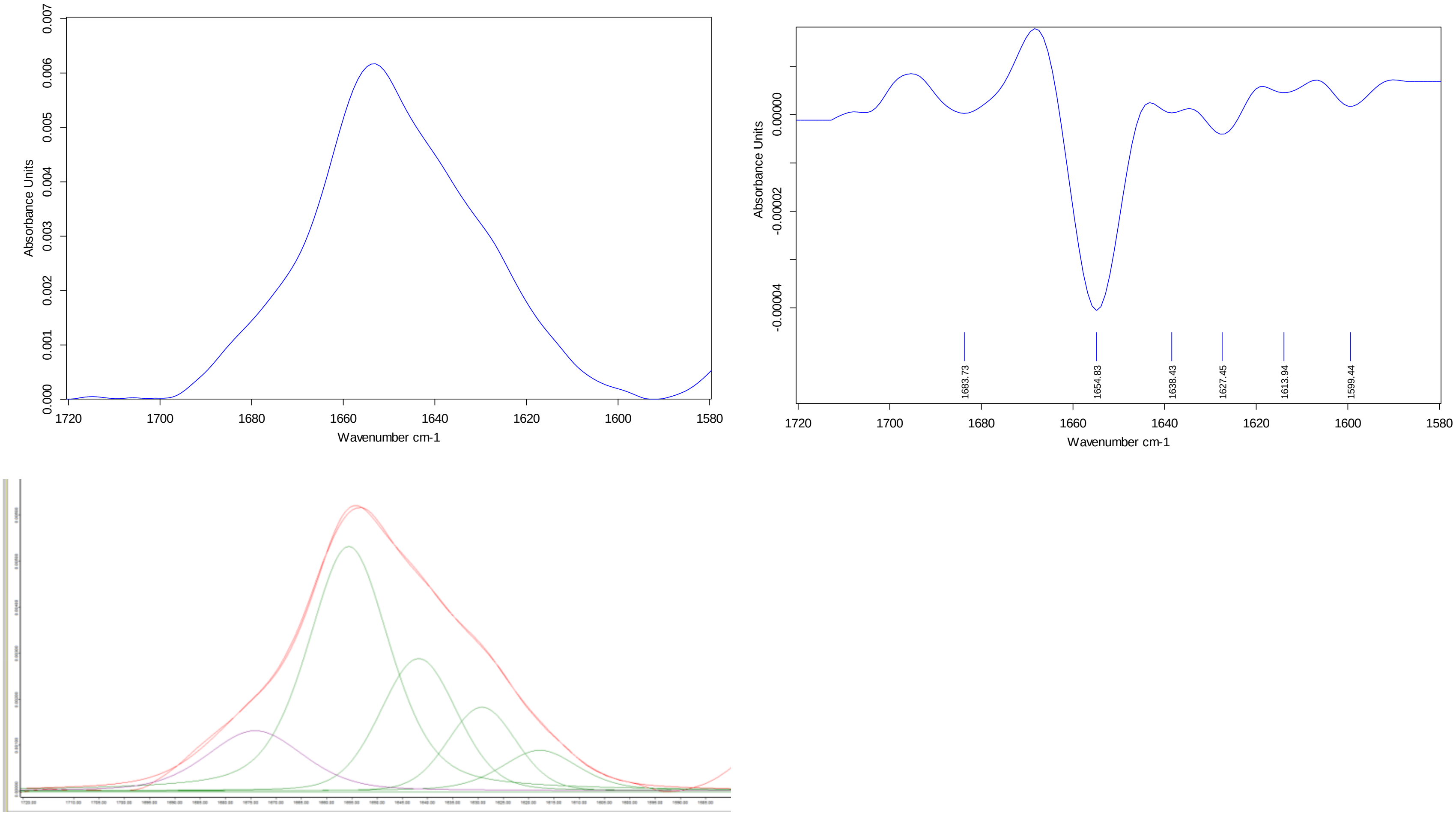

Fig. S34. The original (a), second derivative and deconvoluted ATR-FTIR spectra in Amide I region of native BSA in PBS buffer (pH 7.4, 50 mM) at concentration of 20 mg/mL.

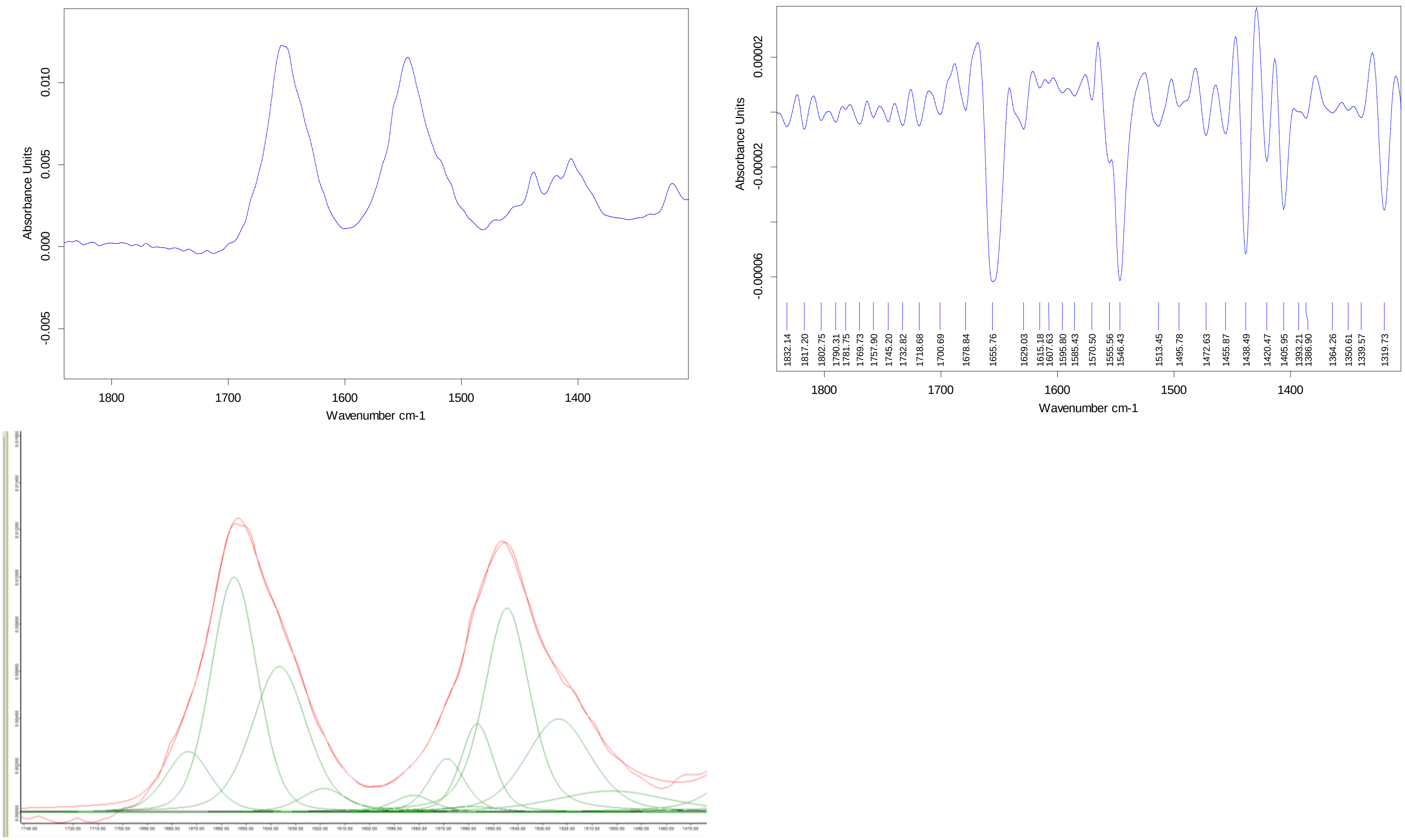

Fig. S35. The original (a), second derivative and deconvoluted ATR-FTIR spectra in Amide I and Amide II region of native BSA-ketoprofen (1:1) in PBS buffer (pH 7.4, 50 mM).

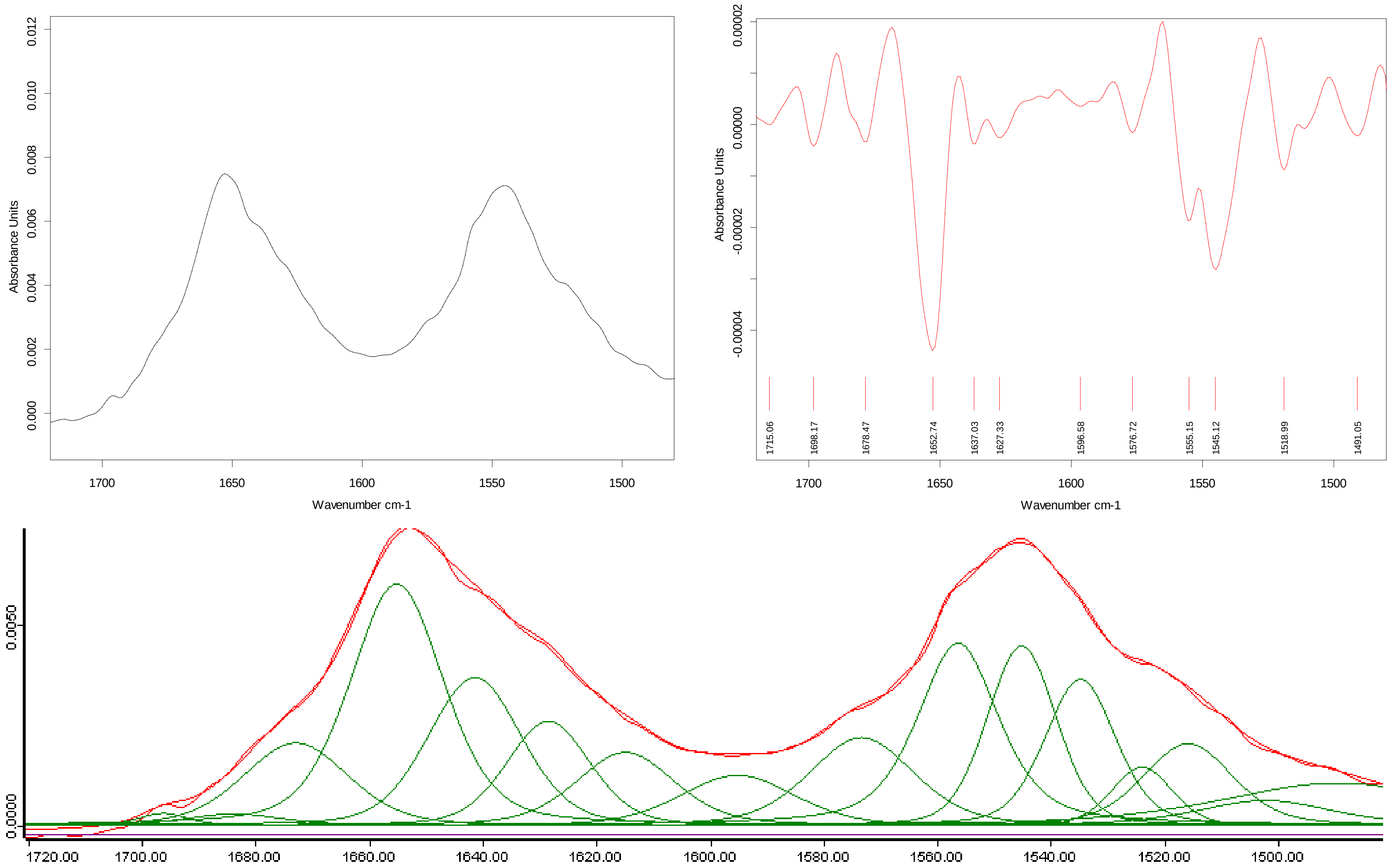

Fig. S36. The original (a), second derivative and deconvoluted ATR-FTIR spectra in Amide I and Amide II region of native BSA-[L-LeuOEt][KETO] (1:1) in PBS buffer (pH 7.4, 50 mM).

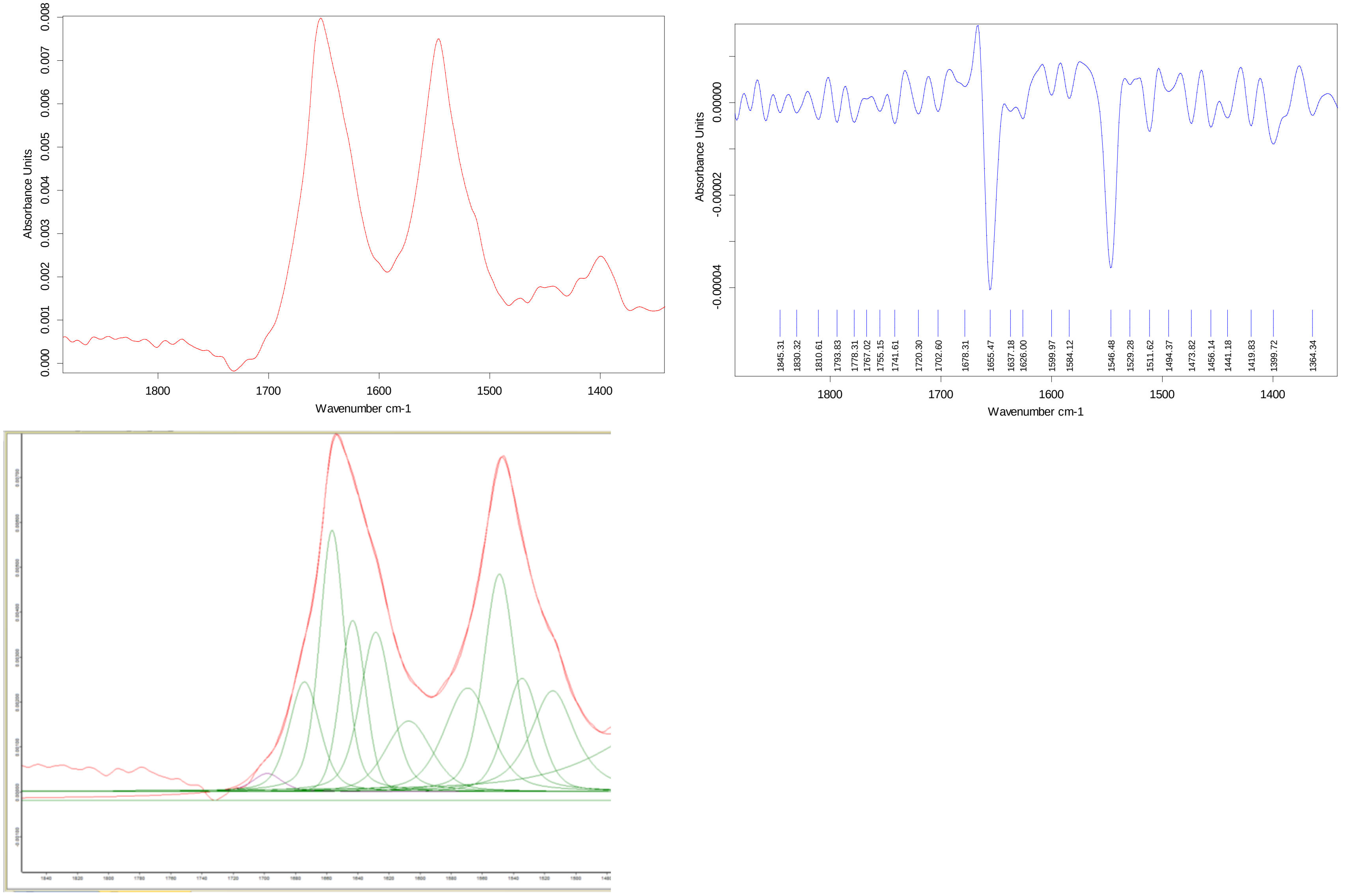

Fig. S37. The original (a), second derivative and deconvoluted ATR-FTIR spectra in Amide I and Amide II region of native BSA-[L-ValOEt][KETO] (1:1) in PBS buffer (pH 7.4, 50 mM).

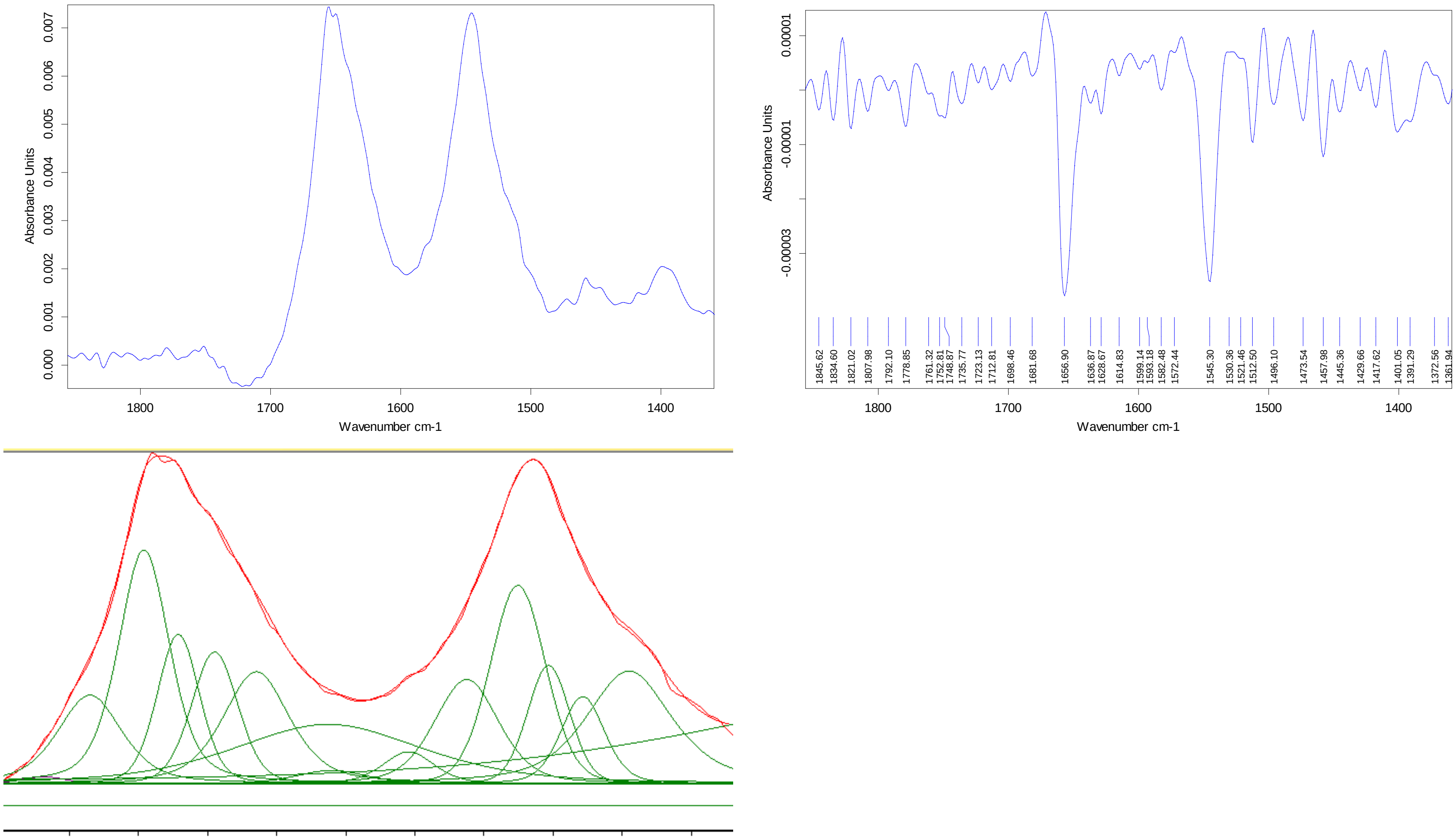

Fig. S38. The original (a), second derivative and deconvoluted ATR-FTIR spectra in Amide I and Amide II region of native BSA-[L-ValOiPr][KETO] (1:1) in PBS buffer (pH 7.4, 50 mM).

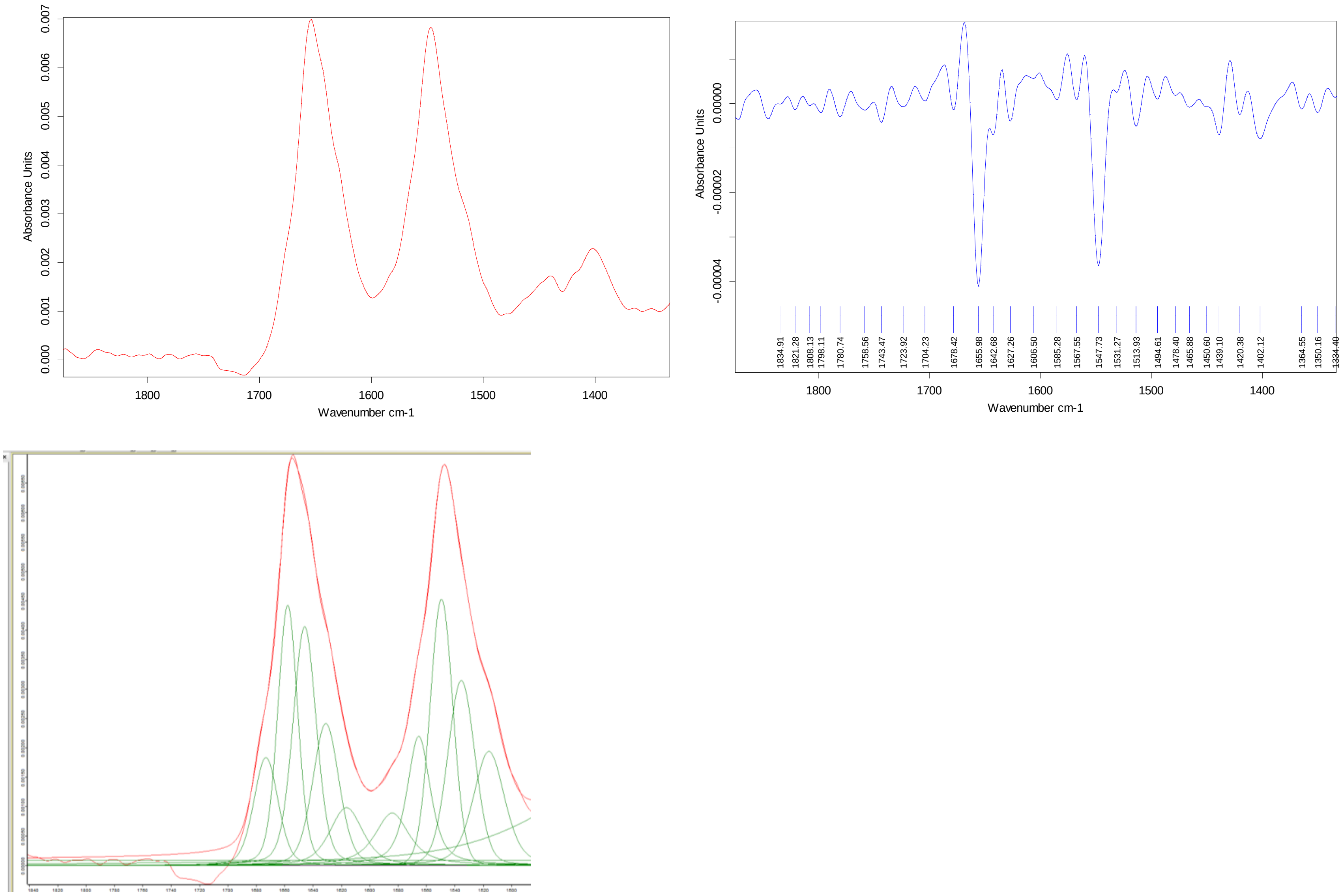

Fig. S39. The original (a), second derivative and deconvoluted ATR-FTIR spectra in Amide I and Amide II region of native BSA-[L-ValOPr][KETO] (1:1) in PBS buffer (pH 7.4, 50 mM).

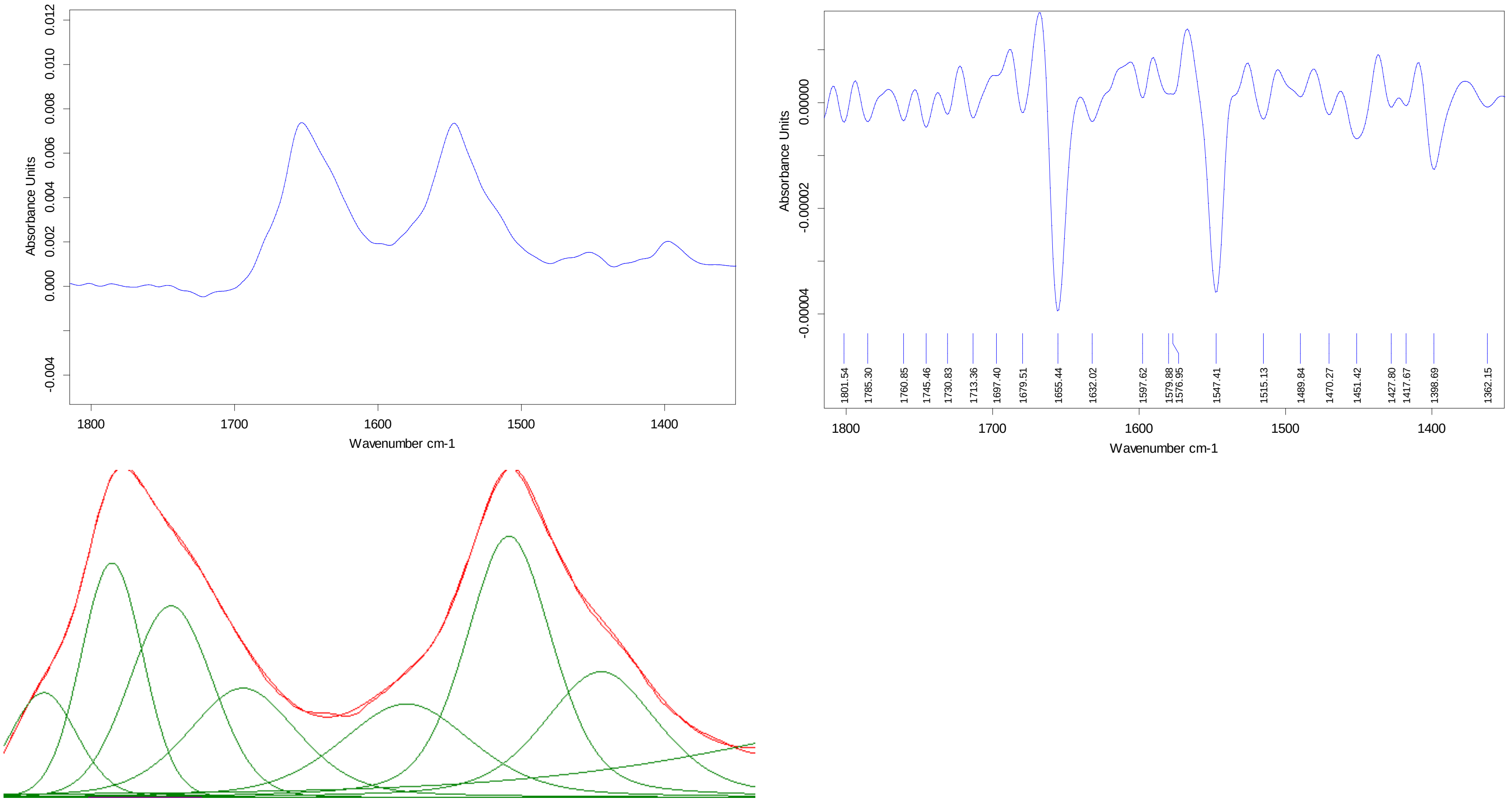

Fig. S40. The original (a), second derivative and deconvoluted ATR-FTIR spectra in Amide I and Amide II region of native BSA-[L-ValOBu][KETO] (1:1) in PBS buffer (pH 7.4, 50 mM).
